# Intratumoral Microbiota in Tumor: Current Understandings and Future Perspectives

**DOI:** 10.1002/mco2.70825

**Published:** 2026-06-23

**Authors:** Jiawei Chen, Yupan Bai, Lu Shen, Jiacheng Ying, Jie Xu, Jiayin Tang, Yujie Bao

**Affiliations:** ^1^ Department of Infectious Diseases, Shanghai Ninth People's Hospital Shanghai Jiao Tong University School of Medicine Shanghai China; ^2^ Department of Gastrointestinal Surgery Renji Hospital Shanghai Jiao Tong University School of Medicine Shanghai China

**Keywords:** immune modulation, intratumoral bacteria, tumor metastasis, tumor microenvironment, tumor precision therapy

## Abstract

Intratumoral bacteria are increasingly recognized as integral and dynamic components of the tumor microenvironment (TME), with profound implications for tumor initiation, progression, metastasis, immune remodeling, and therapeutic response. These microorganisms may originate from mucosal barrier breaches, hematogenous or lymphatic dissemination, local migration from adjacent tissues, or co‐migration with metastatic cells, and can be characterized by sequencing‐based, imaging, culture‐dependent, and multi‐omics approaches. Accumulating evidence indicates that intratumoral bacteria contribute to tumorigenesis by inducing genomic instability, DNA damage, epigenetic alterations, oncogenic signaling, and chronic inflammation. Beyond primary tumor growth, they participate in multiple steps of the metastatic cascade, including epithelial–mesenchymal transition, cytoskeletal remodeling, invasion, intravasation, extravasation, pre‐metastatic niche formation, angiogenesis, and distant colonization. Intratumoral bacteria also reshape immune and non‐immune compartments of the TME, exerting dual effects by promoting immune suppression or enhancing antitumor immunity depending on bacterial species, tumor context, and host immune status. In this review, we summarize the historical development, methodological advances, cancer‐type‐specific diversity, and mechanistic roles of intratumoral microbiota in tumor progression and metastasis. We further discuss their diagnostic and prognostic value, as well as their therapeutic implications for conventional anticancer treatments and microbiota‐targeted strategies, highlighting opportunities and challenges for microbiota‐guided precision oncology.

## Graphical abstract

Intratumoral bacteria are emerging as active regulators of cancer evolution rather than bystanders. This review outlines how tumor‐resident microbes drive tumor initiation and growth by inducing genomic instability, epigenetic reprogramming, oncogenic signaling, and chronic inflammation, while promoting metastatic spread via invasion, angiogenesis, extravasation, and premetastatic niche conditioning. We also highlight bidirectional immune modulation and translational opportunities for microbiome‐informed diagnosis and precision interventions.

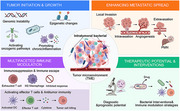

## 1 Introduction

With the rapid expansion of research on the intratumoral microbiome, accumulating evidence indicates that tumor‐resident microorganisms are not merely passive components of the tumor microenvironment (TME) but active participants in cancer pathogenesis [1, 2, 3, 4, 5]. The intratumoral microbiome comprises diverse bacteria, fungi, and viruses, exhibiting marked tissue specificity, compositional diversity, and functional heterogeneity across different cancer types [1, 2, 3, 6]. Among these, intratumoral bacteria have emerged as particularly influential players. Through metabolic reprogramming, immune modulation, and ectopic colonization, they engage in complex crosstalk with host cells and the TME, thereby profoundly shaping tumor progression and metastatic dissemination [6, 7, 8].

Given this emerging link between intratumoral bacteria and metastatic dissemination, understanding bacteria‐regulated metastatic processes is particularly critical, as metastasis represents the ultimate and most lethal manifestation of cancer and remains a major determinant of cancer‐associated mortality and therapeutic failure [9, 10, 11]. Population‐based survival data further highlight the prognostic disadvantage of advanced disease: in the American Cancer Society's Cancer Statistics, 2026 report, the 5‐year relative survival rate for cancers diagnosed during 2015–2021 reached 70% overall, but was only 35% for distant‐stage disease, despite improving from 17% in the mid‐1990s [12]. More recently, a large SEER‐based analysis of 1,030,937 patients with metastatic cancer reported a median survival of only 10 months, with 82.6% of deaths attributable to the diagnosed cancer itself. Therefore, deciphering bacteria‐regulated mechanisms that promote dissemination, survival in circulation, and distant colonization may provide clinically relevant insights into metastatic burden, treatment resistance, and therapeutic failure [13].

Early studies established the presence and basic functional relevance of bacteria within tumors [6]. However, advances in high‐throughput sequencing, spatial profiling, and multi‐omics technologies have substantially deepened our understanding of their roles. Intratumoral bacteria are now recognized to modulate key biological properties of cancer cells, including proliferation, migration, and therapeutic resistance [14, 15]. Moreover, through coordinated immune and metabolic pathways, these microbes reshape the TME to facilitate tumor initiation and metastatic progression [16]. For instance, *Fusobacterium nucleatum* (Fn) has been shown to enhance tumor cell invasiveness by activating oncogenic signaling cascades and to promote immune evasion by establishing an immunosuppressive microenvironment, thereby facilitating tumor escape and metastasis [17]. Beyond its role in tumorigenesis and metastasis, the tumor microbiome also demonstrates significant translational potential in cancer diagnosis, prognosis, and therapy. In the diagnostic and prognostic context, intratumoral microbial composition is increasingly recognized as a novel class of biomarkers. Distinct microbial signatures provide valuable insights for early tumor detection, molecular stratification, and outcome prediction [18, 19]. Therapeutically, strategies targeting intratumoral bacteria—including modulation of microbial communities, targeting of bacterial metabolites, and the use of engineered bacterial vectors [20, 21]—are emerging as promising avenues in oncology research. These innovative approaches hold the potential to overcome limitations of conventional therapies and provide novel solutions for suppressing tumor progression.

Despite substantial progress in this field, several critical challenges remain. First, most existing studies are limited by relatively small sample sizes, and the heterogeneity of microbial composition across different tumor types, disease stages, and patient populations has not yet been comprehensively characterized [22]. Second, the causal relationship between microbial colonization and tumor progression requires further validation through rigorously designed in vivo experiments and prospective clinical studies [21]. Third, the low microbial biomass within tumors, together with the potential for technical contamination, may compromise the accuracy and reliability of sequencing‐based analyses [23, 24, 25]. These limitations underscore the urgent need for standardized experimental workflows and robust bioinformatic pipelines.

To address these complex challenges, this review systematically synthesizes current evidence regarding the multilayered interactions among intratumoral microorganisms, tumor cells, and the immune microenvironment. We first outline the research foundations and methodological advances that have driven this field, followed by a discussion on the origins, colonization patterns, and compositional diversity of intratumoral bacteria. Subsequently, we elucidate the diverse mechanisms by which these resident bacteria drive tumor initiation and progression, their specific roles across different stages of the metastatic cascade, and their intricate crosstalk with the immune microenvironment. Finally, we highlight the emerging clinical translational value of the tumor microbiome, with a specific focus on microbiome‐informed strategies for future precision oncology.

## 2 Historical and Methodological Foundations of Intratumoral Microbiome Research

The evolution of intratumoral microbiome research has been intrinsically intertwined with underlying technological advancements. From early microscopic observations of microbes within tumors to the current high‐resolution mapping of complex microecosystems using multi‐omics, methodological leaps have fundamentally driven our conceptual shift regarding the tumor microenvironment. This section traces the pivotal historical milestones in this field and systematically reviews the state‐of‐the‐art technologies used to characterize intratumoral microbial communities, providing essential context for the mechanistic discussions that follow.

### 2.1 Milestone Historical Events of Intratumoral Microbiome

**FIGURE 1:**
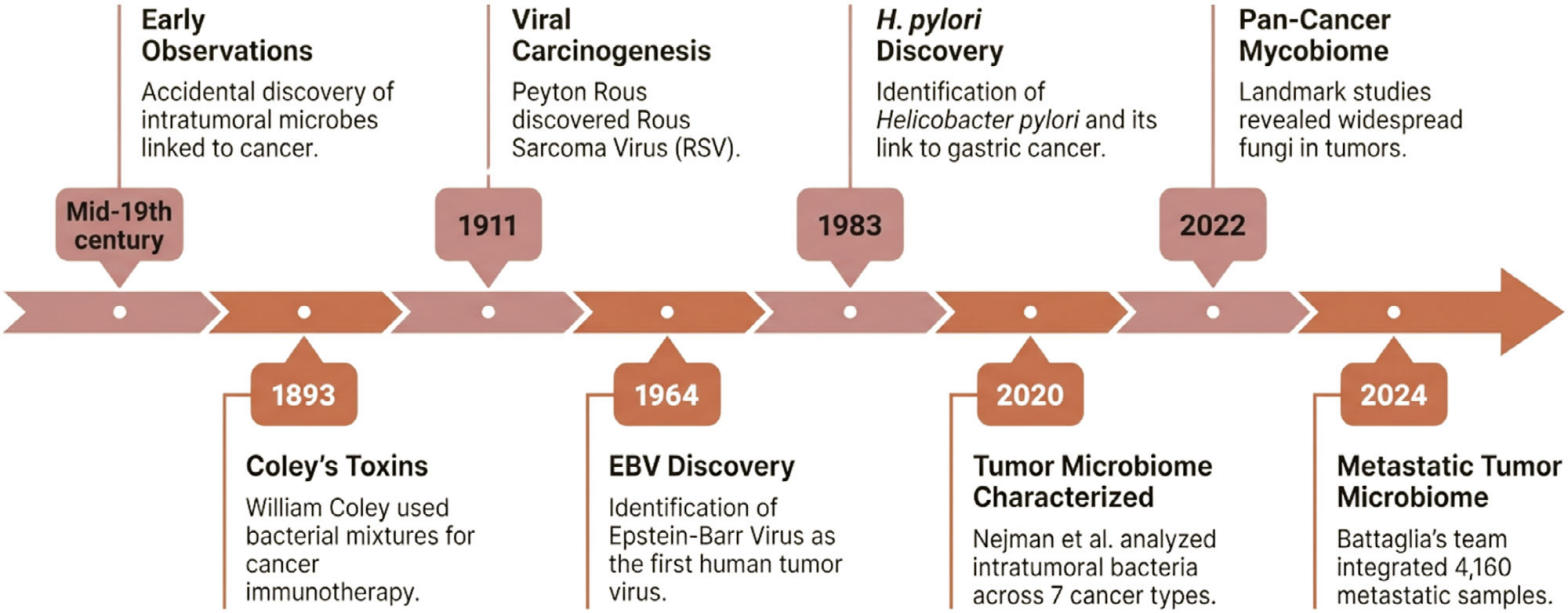
Schematic timeline summarizing seminal discoveries that shaped the concept of intratumoral microbiota. Milestones include early observations of microbes in tumor tissues (mid‐19th century), Coley's bacterial mixtures as early cancer immunotherapy (1893), the emergence of oncogenic virology with RSV (1911) and EBV (1964), the isolation of *H. pylori* and its link to gastric carcinogenesis (1983), and the high‐throughput sequencing era enabling systematic characterization of the tumor microbiome (2020). The framework was subsequently extended to fungi through pan‐cancer mycobiome profiling (2022), and further expanded to metastatic disease via a pan‐cancer metastatic microbiome atlas (2024). Recent advances in single‐cell and spatially resolved approaches provide mechanistic and localization‐aware insights into host–microbe interactions within tumors. EBV, Epstein–Barr virus; RSV, Rous sarcoma virus.

From the earliest observations in the mid‐19th century, when microbes were detected in tumor tissues using microscopy and culture‐based approaches, the concept of a cancer–microbe connection has evolved through several seminal milestones (Figure 1). In 1893, Coley's use of bacterial mixtures for cancer treatment provided an early proof‐of‐principle that microbial stimuli can modulate antitumor immunity [26, 27]. The virology era further reshaped this field, beginning with Rous's discovery of Rous sarcoma virus in 1911 through transmissibility experiments [28] and culminating in the identification of Epstein–Barr virus in 1964 using electron microscopy and serological techniques [29], establishing viruses as bona fide oncogenic agents.

A major turning point came in 1983, when Marshall and Warren isolated *Helicobacter pylori* through culture‐based methods and histopathological examination, providing direct evidence that chronic bacterial infection can drive carcinogenesis via sustained inflammation [30, 31]. With the advent of high‐throughput sequencing technologies, including 16S rRNA gene sequencing, metagenomics, and later transcriptomic approaches, tumor microbiome research accelerated rapidly, transitioning from tumor‐specific observations to systematic pan‐cancer mapping. A landmark study by Nejman et al., which integrated sequencing, imaging, and rigorous contamination controls (including negative controls and reagent background filtering), profiled 1526 tumors across seven cancer types and demonstrated that intratumoral bacteria are prevalent, tissue‐specific, metabolically active, and frequently localized intracellularly [1]. Subsequent studies expanded this framework beyond bacteria: pan‐cancer mycobiome analyses based on sequencing datasets revealed widespread fungal signals across tumors [2, 3], although questions regarding environmental and sampling contamination have remained an active area of methodological debate in low‐biomass tissues. Most recently, Battaglia and colleagues [32] integrated 4160 metastatic samples to generate a metastatic microbiome atlas, highlighting associations with immunotherapy outcomes while applying computational decontamination strategies to strengthen robustness. Together with advances in single‐cell and spatial transcriptomics [33, 34, 35], these milestones are propelling the field toward spatially resolved, mechanism‐driven models of how intratumoral microbiota shapes tumor initiation, progression, metastasis, and therapeutic response.

### 2.2 Methods Used to Characterize the Intratumoral Microbiome

Recent advances in high‐throughput sequencing and spatial omics have fundamentally reshaped intratumoral microbiome research, enabling a shift from the simple question of whether microbes are present toward a hierarchical framework that interrogates where they reside, whether they are viable, and whether they exert biological functions. Because tumor tissues typically harbor extremely low microbial biomass and are dominated by host nucleic acids, available detection strategies differ substantially in sensitivity, resolution, and interpretability. To systematically evaluate the methodological rigor and evidentiary strength of prior studies, we classify current approaches into three tiers: (i) genomics‐based methods that primarily provide correlative evidence, (ii) non‐genomic spatial localization techniques that establish histological evidence, and (iii) viability‐ and function‐oriented assays that support biological relevance (Table 1).

**TABLE 1:** Detection methods for intratumoral microbiota and corresponding levels of evidence.

Detection technologies	Detection materials	Main advantages	Main limitations	Level of evidence	Ref.
16S rRNA gene sequencing	Hypervariable regions of bacterial 16S rRNA	Cost‐effective; rapid; suitable for community profiling and comparative analyses	Limited taxonomic resolution; PCR bias; highly sensitive to contamination; unable to distinguish live vs. dead bacteria	Associative (presence‐level)	[36, 37]
Shotgun metagenomic sequencing (WMS)	Total DNA from tumor tissue	High taxonomic resolution; detects bacteria, viruses, fungi; enables functional inference	High cost; host DNA contamination; complex bioinformatics; low signal‐to‐noise ratio in low‐biomass samples	Associative–functional inference	[38]
Metatranscriptomics	Total RNA (bacterial mRNA)	Identifies transcriptionally active microbes; reflects metabolic activity	RNA instability; host RNA contamination; high cost; limited sensitivity	Putative activity‐level	[39, 40]
IS‐pro	16S–23S rRNA intergenic spacer regions	Fast; species‐level discrimination; low DNA input	Proprietary platform; limited functional information	Associative (presence‐level)	[41]
IHC	Microbial antigens (e.g., LPS, LTA)	Low cost; easy implementation; tissue context preserved	High false‐positive rate; cannot distinguish live bacteria from remnants	Spatial associative	[42]
FISH	rRNA‐targeted probes	Species‐specific; precise spatial localization; single‐cell resolution	Requires sufficient microbial abundance; limited functional insight	Spatial localization (structural evidence)	[43]
CLEM/TEM/EM	Tissue ultrastructural sections	Direct visualization; subcellular localization; high specificity	Expensive; technically demanding; low throughput	High‐confidence spatial evidence	[1, 44]
Microbial culture	Fresh tumor tissues	Gold standard for live bacteria; enables downstream functional assays	Low sensitivity; culture bias; contamination risk	Viability‐level (causal potential)	[45, 46]
Fluorescent metabolic labeling (e.g., D‐amino acids)	Actively synthesized bacterial cell wall	Directly labels metabolically active bacteria; high specificity	Requires fresh tissue; ex vivo only; may miss dormant bacteria	Direct viability evidence	[47]
Proteomics (LC‐MS)	Microbial‐derived proteins	Identifies functional microbial components; host–microbe interaction clues	Low abundance; annotation ambiguity; cannot confirm viability	Functional association	[48, 49]
Metabolomics	Microbial‐derived metabolites	Reveals microbial functional output; pathway‐level insights	Difficult source attribution; indirect evidence	Functional association	[50]

Abbreviations: 16S rRNA, 16S ribosomal RNA; 23S rRNA, 23S ribosomal RNA; CLEM, correlative light and electron microscopy; D‐amino acids, D‐AAs; DNA, deoxyribonucleic acid; EM, electron microscopy; FISH, fluorescence in situ hybridization; IHC, immunohistochemistry; IS‐pro, interspace profiling; LC‐MS, liquid chromatography‐mass spectrometry; LPS, lipopolysaccharide; LTA, lipoteichoic acid; mRNA, messenger RNA; PCR, polymerase chain reaction; RNA, ribonucleic acid; rRNA, ribosomal RNA; TEM, transmission electron microscopy; WMS, whole‐metagenome shotgun sequencing.

#### 2.2.1 Genomics‐Based Approaches

Next‐generation sequencing remains the core methodology for intratumoral microbiota research, including prokaryotic 16S ribosomal RNA (16S rRNA) gene sequencing [36, 37], whole‐metagenome shotgun sequencing (WMS) [38], metatranscriptomics [39, 40], and interspace profiling (IS‐pro) [41]. Together, these approaches enable taxonomic identification and functional inference of microbial communities within tumor tissues.

16S rRNA sequencing is a widely applied approach for profiling bacterial and archaeal communities [36, 37]. This method has been extensively used to characterize microbial composition, phylogenetic relationships, and community diversity across multiple tumor types, including breast cancer [51] and colorectal cancer (CRC) [52]. Because it targets specific hypervariable regions rather than the entire genome, 16S rRNA sequencing offers advantages of rapid detection, relatively low cost, and suitability for low‐input samples, and can achieve taxonomic classification at the species or strain level under optimized conditions [53, 54]. However, its relatively limited resolution often precludes discrimination between closely related taxa, and PCR amplification bias may lead to overestimation of microbial diversity [53].

Compared with 16S rRNA sequencing, shotgun metagenomic sequencing provides higher‐resolution microbial profiles by capturing the entire genomic content of a sample, enabling detection of bacteria, viruses, and fungi, as well as functional pathway analysis. However, because this approach requires sequencing all DNA present in the sample‐including host‐derived DNA from normal and tumor cells, it is more time consuming, technically complex, and costly [55].

Metatranscriptomics captures transcribed bacterial RNA and thereby enriches for metabolically active microorganisms. Despite this advantage, several limitations remain, including the short half‐life of RNA molecules, high sensitivity to host RNA contamination (particularly rRNA), and elevated experimental costs [39, 40, 56]. Integration of metatranscriptomics with single‐cell technologies may represent a promising future direction for dissecting tumor–microbe interactions at higher resolution. IS‐pro is a sequencing‐based approach similar to 16S rRNA sequencing that detects microbial DNA and enables rapid species‐level discrimination based on variability in intergenic spacer regions.

Overall, due to the extremely low microbial biomass in tumor tissues, all genomics‐based approaches are highly susceptible to contamination and are unable to reliably distinguish viable microorganisms from residual microbial DNA fragments. These limitations remain a major bottleneck in intratumoral microbiota research and underscore the need for multimodal validation strategies.

#### 2.2.2 Nongenomic Spatial Localization Approaches

To overcome the inability of sequencing‐based methods to localize microbial signals within tissue architecture, non‐genomic spatial techniques such as immunohistochemistry (IHC), fluorescence in situ hybridization (FISH), and advanced microscopy have been widely applied [1, 43, 44, 56, 57, 58].

IHC is a conventional detection method based on antigen–antibody specificity, typically targeting bacterial lipopolysaccharide (LPS) or lipoteichoic acid (LTA) [42]. This approach is cost‐effective and technically accessible; however, it is associated with a relatively high false‐positive rate. For example, even in the absence of viable bacteria, prolonged phagocytosis can result in persistent LPS/LTA‐positive signals, necessitating validation using complementary techniques.

FISH employs rRNA‐specific probes to identify microbial cells and enables visualization, sorting, and single‐cell quantification of low‐abundance microorganisms with high spatial precision [43]. Correlative light and electron microscopy (CLEM) and transmission electron microscopy (TEM) provide ultrastructural evidence of microbial localization within tumor cells or tissue compartments [1, 44]. Although spatial approaches substantially strengthen structural evidence by demonstrating in situ localization, they inherently detect stable, non‐dynamic microbial components—such as cell wall antigens, nucleic acids, or ultrastructural remnants—rather than indicators of microbial activity or metabolic state.

Consequently, these methods effectively address the question of “where microorganisms are located” and elevate evidence from mere association to histological localization, but they cannot determine whether detected microbes are viable or functionally active within the TME. Bridging this gap requires integration of spatial localization techniques with activity‐reflecting approaches, such as metatranscriptomics, metabolomics, metabolic labeling, or live imaging, to link structural presence with biological function.

#### 2.2.3 Viability‐ and Function‐Oriented Approaches

Verification of microbial viability and functional activity is critical for overcoming the limitations of genomics‐based approaches and establishing a multi‐layered evidence framework encompassing presence, activity, and function.

Microbial culture remains the gold standard for demonstrating the existence of viable intratumoral bacteria and enables isolation of live microorganisms from freshly resected tumor tissues. Pure cultures can subsequently be used for functional studies [45, 46]. However, conventional culture approaches suffer from limited sensitivity, strong culture ability bias, and high contamination risk. Recent advances, including reverse genomics approaches based on epitope capture [59] and organoid‐based co‐culture platforms [56, 60], have partially mitigated these limitations and enabled enrichment of otherwise difficult‐to‐culture microorganisms.

Notably, Straussman and colleagues recently applied fluorescent D‐alanine metabolic labeling to freshly resected brain tumor slices, directly visualizing peptidoglycan synthesis and providing compelling evidence for metabolically active bacteria within tumor tissues [47]. This approach relies on the incorporation of D‐alanine into bacterial cell wall peptidoglycan [61], thereby selectively labeling proliferating bacteria [62]. Despite its advantages for detecting viable bacteria, this strategy requires fresh tissue, is restricted to ex vivo settings, and may fail to detect dormant or slow‐growing bacteria.

Proteomics, primarily based on liquid chromatography‐mass spectrometry (LC‐MS) platforms, enables identification of microbial‐derived proteins differentially expressed between tumor and normal tissues, providing insights into potential functional interactions between microbes and host cells [48, 49]. However, low microbial protein abundance, high sequence homology, and annotation ambiguity limit its sensitivity and accuracy, and it cannot distinguish viable bacteria from residual proteins. Metabolomics enables systematic analysis of microbial‐derived metabolites within the TME, such as short‐chain fatty acids (SCFAs), bile acids, and indole derivatives [50], which have been implicated in immune modulation, inflammation, and signaling pathway regulation [56]. Nonetheless, metabolite origin attribution remains challenging, and even spatially resolved metabolomics cannot directly indicate microbial viability.

#### 2.2.4 Low Biomass and Host DNA Contamination: Shared Bottlenecks and Mitigation Strategies

Due to the inherent low‐biomass nature of tumor tissues, environmental colonizers, reagent‐derived contaminants, and exogenous DNA introduced during sample processing can all be amplified into “detectable signals” at the sequencing level, significantly confounding the interpretation of results. This issue is especially pronounced in low‐biomass materials such as urinary tract tissues, necrotic tumor regions, or formalin‐fixed paraffin‐embedded (FFPE) samples. Consequently, studies that rely solely on high‐throughput sequencing without rigorous contamination assessment and control often struggle with attributing microbial signals, ensuring cross‐cohort reproducibility, and establishing cancer‐type specificity [63, 64, 65]. Furthermore, established research has clearly indicated that reagent and laboratory contamination can critically affect sequence‐based microbiome analyses in low‐biomass samples, sometimes dominating the apparent microbial community composition and leading to systematic false‐positive conclusions [23].

To increase the reliability of these findings, many studies now incorporate multi‐tier negative controls at the experimental design stage (e.g., sampling blanks, DNA extraction blanks, and library preparation blanks), which are processed and sequenced alongside biological samples. This approach allows for the quantification of background contamination and identification of process‐specific “contamination fingerprints” [63]. In the bioinformatics analysis phase, efficient host‐read removal and contamination modeling have become essential components of intratumoral microbiota research workflows. Typically, high‐quality alignment algorithms are employed to filter human genome reference‐based reads, removing host‐derived sequences, while statistical methods using negative control abundance distributions are used to infer and denoise potential contamination signals, thus minimizing the impact of reagent or environmental contaminants on downstream analyses [64, 65].

Importantly, certain tissues, such as the urinary tract or mucosa adjacent to tumors, may harbor low‐abundance resident microbial communities, further complicating the distinction between “true intratumoral microbes” and “incidental signals/contamination” [63]. As a result, sequencing data alone often cannot provide robust mechanistic insights. Combining sequencing results with spatial localization evidence (e.g., FISH/IHC) and viability/functional evidence (e.g., metabolic labeling, culture, transcriptomics, or functional assays) has become a key strategy for answering critical questions: “Where is the microbe?”; “Is it viable/active?”; and “Does it have functional consequences?” This integrated approach not only elevates the confidence in evidence but also minimizes the risk of over‐interpretation [63]. Furthermore, methodological reviews in low‐biomass microbiome studies emphasize the importance of spatial imaging and in situ detection for establishing reliable localization. However, to substantiate the functionality of microbes, it is necessary to complement these approaches with activity measures and mechanistic validation [24].

Collectively, accurate characterization of the intratumoral microbiome remains technically challenging, and discrepancies in detection strategies can substantially influence data interpretation and the strength of biological conclusions. Future efforts should prioritize standardized workflows and tighter evidence loops that integrate contamination control, spatial mapping, and functional validation, thereby enabling a more reliable transition from microbial presence to mechanistic insight and translational applications.

## 3 Characterization of Intratumoral Bacteria

The presence of intratumoral bacteria has been confirmed in various solid tumors, yet their origins and colonization mechanisms remain important questions to be addressed. The unique physiological characteristics of tumor tissue create possibilities for microbial infiltration and survival. This article will discuss these aspects by first analyzing the potential sources and migration pathways of intratumoral bacteria, followed by an exploration of how the tumor microenvironment influences bacterial selection, adaptation, and long‐term colonization.

### 3.1 Origin and Migration of Intratumoral Bacteria

**FIGURE 2:**
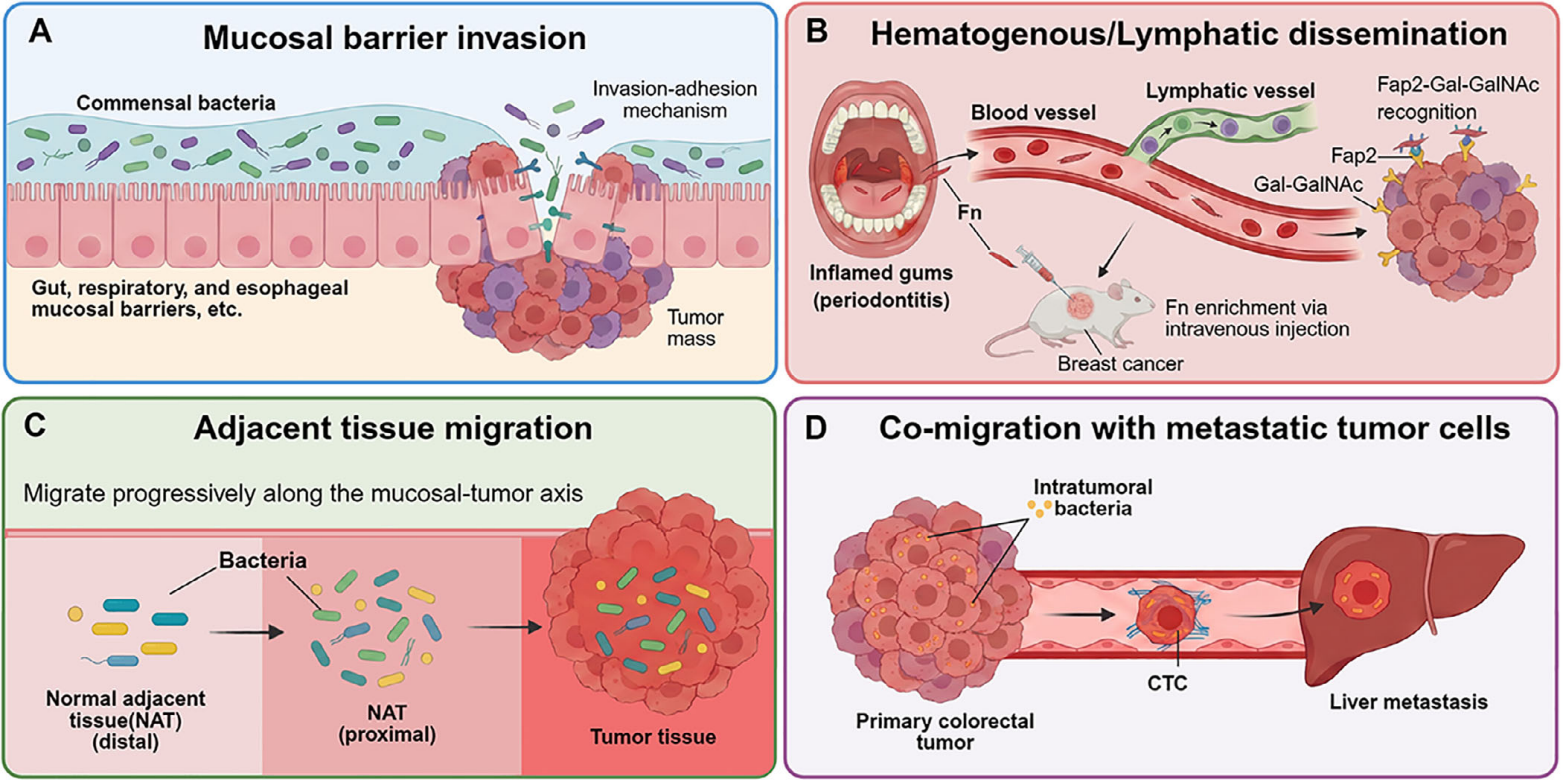
Origin of intratumoral bacteria. Multiple tumor types harbor heterogeneous bacterial colonization, yet the origins and mechanisms remain incompletely defined. Four major routes are proposed. **(A)** Mucosal barrier disruption: local tumorigenesis compromises epithelial integrity, permitting commensal microbiota (e.g., from the gut or respiratory tract) to invade via adhesion–invasion processes. **(B)** Hematogenous or lymphatic spread: microorganisms from primary infection sites enter circulation; for example, Fn, linked to periodontitis, disseminates through bacteremia and targets CRC cells via Fap2‐Gal‐GalNAc binding. **(C)** Adjacent tissue infiltration: similarities between microbiota in neighboring tissues and tumors suggest progressive mucosal‐tumor migration. **(D)** Co‐migration with metastasizing tumor cells: certain bacteria accompany CTCs to distant organs, as reported in CRC liver metastases and breast cancer, where intracellular bacteria remodel cytoskeletal dynamics to enhance colonization. Fn, *Fusobacterium nucleatum*; Fap2, Fusobacterium autotransporter protein 2; Gal‐GalNAc, galactose‐N‐acetylgalactosamine; CRC, colorectal cancer; CTCs, circulating tumor cells.

Despite the widespread detection of heterogeneous bacterial populations within tumors, their origins and mechanisms of colonization remain fundamental questions. Based on current evidence, four representative pathways may contribute to the origin and migration of intratumoral bacteria (Figure 2).

#### 3.1.1 Mucosal Barrier Breach

The mucosal surfaces of human cavitary organs harbor abundant commensal microorganisms, making barrier disruption a plausible route for bacterial entry into tumor tissues [66]. In the digestive tract, studies in colorectal neoplasia have shown increased positivity and relative abundance of enterotoxigenic *Bacteroides fragilis* (ETBF) and Fn in early lesions, and paired analyses of distal NAT, proximal NAT, and tumor tissue further support progressive migration of mucosa‐associated bacteria along the mucosal‐tumor axis [67, 68]. In the esophagus, paired tumor and tumor‐adjacent tissue studies in esophageal squamous cell carcinoma (ESCC) have demonstrated substantial overlap together with selective enrichment of specific taxa, suggesting that local esophageal mucosal communities may serve as a proximal source of intratumoral colonization after epithelial barrier disruption [69]. For the respiratory tract, evidence is more indirect, but comparisons of bronchoalveolar lavage fluid, lung tumor tissue, and matched lung controls, together with paired tumor‐normal lung analyses, support continuity between lower airway microbial communities and lung tumor‐associated microbial patterns [70]. Collectively, these findings support mucosal barrier breach as a plausible and evidence‐based route for intratumoral bacterial colonization, with the strongest support currently coming from intestinal/colorectal and esophageal settings.

#### 3.1.2 Hematogenous or Lymphatic Dissemination

The circulatory system serves as a hematogenous route for microorganisms to disseminate from an initial infection focus and seed tumor sites. Studies have confirmed that periodontitis‐associated Fn can enter the bloodstream during bacteremia and specifically recognize the receptor polysaccharide D‐galactose‐β(1‐3)‐N‐acetyl‐D‐galactosamine (Gal‐GalNAc) on the surface of tumor cells via its surface lectin Fap2, achieving targeted colonization in CRC [71]. Animal models further indicated that intravenous injection of Fap2^+^ Fn can lead to the enrichment of intratumor flora in breast cancer model mice, suggesting the possibility of hematogenous dissemination across cancer types [72].

#### 3.1.3 Local Migration From Adjacent Tissue

Tumor tissues and normal adjacent tissues (NAT) often share some of the flora characteristics [1, 73]. CRC studies have found that the bacterial composition in the distal NAT, proximal NAT, and tumor tissue of the same patient is highly similar, suggesting that microorganisms may migrate progressively along the mucosal‐tumor axis [73]. Notably, it has been suggested that the NAT flora may not be the primary source, but rather a “transit area” for microorganisms to infiltrate the tumor after breaking through the mucosal barrier [21].

#### 3.1.4 Co‐Migration With Metastatic Cells

Recent evidence suggests that specific intratumoral flora can spread accompanied with metastatic cancer cells to distant organs. For example, the persistence of Fn in liver metastases of CRC, which is identical to that of the primary site, suggests the synergistic survival of microbial‐cancer cell symbionts during metastasis [74]. In addition, Fu et al. found in a study of a spontaneous mouse breast cancer model that a specific bacteria group settled within tumor cells were able to accompany cancer cells across the circulatory system. Mechanistically, these intracellular bacteria promote metastatic colonization by remodeling the host cytoskeleton and enhancing the resistance of circulating tumor cells (CTCs) to blood flow shear forces [51].

### 3.2 Colonization of Intratumoral Bacteria

The processes by which microorganisms are “attracted to, delivered to, retained within, and expanded in” tumor tissues are not passive events. Rather, they are orchestrated by multiple interacting factors, including metabolic niche selection, vascular delivery windows, immune clearance pressure, and receptor‐adhesin compatibility. Together, these determinants shape tumor type‐specific microbial composition and spatial distribution [75, 76, 77].

First, metabolic reprogramming of tumor cells—most notably the Warburg effect [78]—creates a local microenvironment characterized by hypoxia, acidosis, and steep nutrient/metabolite gradients. These conditions confer a selective advantage to facultative and obligate anaerobes and provide the theoretical foundation for employing anaerobic bacteria as tumor‐targeting vectors or oncolytic agents [75, 77]. For example, *Clostridium novyi*‐NT spores selectively germinate and proliferate within severely hypoxic regions of solid tumors, indicating that the hypoxic core constitutes a critical ecological niche for strict anaerobic colonization [75]. Similarly, the anaerobic/microaerophilic probiotic *Bifidobacterium longum*, following systemic administration, has been selectively detected in mammary tumor tissues while remaining largely undetectable in most normal organs, supporting the concept of TME‐driven tissue selectivity [79].

Beyond oxygen deprivation, tumor metabolic rewiring generates elevated lactate levels and bile acid‐associated metabolic imbalances, which may confer competitive advantages to metabolically adaptable taxa‐particularly members of the phylum Proteobacteria and the family Enterobacteriaceae. In pancreatic ductal adenocarcinoma (PDAC), diverse bacterial communities often dominated by *Proteobacteria* are enriched in tumor tissues, where they correlate with immunosuppressive programs and tumor‐associated phenotypes [80, 81]. Notably, intratumoral *Gammaproteobacteria* in PDAC have been shown to express cytidine deaminase, thereby inactivating gemcitabine and demonstrating a clear functional adaptation within this tumor niche [82].

Although it remains to be conclusively established whether elevated lactate levels or bile acid dysregulation directly drive the dominance of specific Proteobacterial taxa (e.g., *Enterobacter* spp.) within tumors, mechanistic insights from intestinal inflammation models provide supportive evidence. Host‐derived lactate has been shown to promote *Enterobacteriaceae* expansion during colitis [83], and bile acids can reshape the colonic metabolic landscape to facilitate *Enterobacteriaceae* colonization and proliferation [84]. Collectively, these findings offer mechanistic parallels supporting the testable hypothesis that tumor metabolic landscapes selectively favor specific microbial phyla or strains.

Second, tumor growth is frequently accompanied by necrosis and the release of stress‐associated metabolites, generating chemotactic gradients that guide directional bacterial migration and promote enrichment within necrotic and hypoxic regions. Using *Salmonella enterica* serovar Typhimurium (*S. typhimurium*) as a model, intratumoral chemotaxis and migration have been shown to depend on specific chemoreceptors. Deletion of ribose and galactose chemoreceptors increases preferential localization within tumor quiescent zones and enhances coverage of deeper tumor regions, suggesting that chemical cues released by quiescent tumor cells may serve as critical navigational signals for bacterial homing [76]. These findings provide direct mechanistic insight into why distinct spatial compartments within the same tumor may harbor heterogeneous microbial distributions.

Third, although tumor‐associated angiogenesis increases overall perfusion, neovasculature is structurally abnormal, characterized by incomplete endothelial junctions and elevated permeability. When coupled with focal hemorrhage and altered interstitial pressure, these features facilitate the retention of blood‐borne microorganisms within the tumor microvascular bed and enhance their extravasation into the stroma, thereby creating a relatively permissive colonization window [33, 77]. This phenomenon is further supported by biodistribution studies of attenuated bacterial therapeutics. For example, the genetically modified strain *S. typhimurium* VNP20009 demonstrates preferential accumulation in tumors of tumor‐bearing animals, indicating that circulating bacteria can be effectively “captured” within the tumor vasculature and subsequently establish tissue colonization [85].

Finally, tumor‐specific receptor expression profiles may interact with microbial surface adhesins, further reinforcing tumor type‐ and stage‐specific microbial selection. A representative example is Fn, whose adhesin Fap2 binds to Gal‐GalNAc moieties expressed on the surface of tumor cells, thereby explaining its selective enrichment in CRC [71]. This receptor‐adhesin interaction has also been experimentally implicated in breast cancer colonization and metastatic progression, suggesting that alterations in the tumor glycosylation landscape directly influence microbial tissue tropism and spatial distribution [72].

Collectively, the causal determinants of intratumoral microbial colonization should be interpreted within an integrated framework encompassing metabolic selection pressures, chemotactic navigation, vascular delivery/retention dynamics, immune clearance gating, and molecular adhesion recognition. Future integration of spatial omics, in situ metabolic imaging, and traceable labeling systems is expected to enable dynamic characterization of the entire “arrival‐to‐colonization” process and facilitate the formulation of testable mechanistic models [81].

## 4 Diversity of Intratumoral Bacteria in Different Cancers

Nejman et al. conducted a comprehensive analysis across seven tumor types—breast, lung, ovarian, pancreatic, melanoma, bone, and brain cancers—and confirmed the presence of bacterial signals in all examined solid tumors. Notably, microbial compositional similarity was significantly higher among samples within the same cancer type than between distinct tumor types [1]. These findings support the widespread presence of intratumoral bacteria across diverse malignancies and suggest that their distribution is unlikely to be solely attributable to stochastic contamination, but rather reflects selective shaping by the TME.

Pan‐cancer comparisons further indicate that individual tumor types harbor relatively stable and distinguishable “microbial fingerprints.” Overall, while the phyla *Proteobacteria* and *Firmicutes* broadly predominate, systematic shifts in community structure—particularly variations in the *Proteobacteria*‐to‐*Firmicutes* ratio—shape distinct, cancer type‐specific microbial configurations [1]. This ecological and spatial differentiation becomes even more pronounced at the organ‐specific level. For example, CRC tissues are more frequently enriched in *Firmicutes* and *Bacteroidetes* [80], whereas PDAC more commonly exhibits signals dominated by *Gammaproteobacteria* within the phylum *Proteobacteria* [80]. These observations suggest that distinct tumor types may selectively accommodate specific microbial taxa.

Beyond compositional differences, available evidence suggests that the overall intratumoral microbial burden is not uniformly distributed across tumor types. Nejman et al. quantified bacterial DNA and reported marked cancer‐type‐dependent variation in bacterial DNA positivity, ranging from only 14.3% of melanoma samples to more than 60% of breast, pancreatic, and bone tumors. This finding indicates that the magnitude of detectable intratumoral bacterial signal differs substantially among malignancies [1]. Consistent with this view, pan‐cancer microbial analyses also reported cancer‐type‐level differences in bacterial load, with breast and bone cancers showing among the highest bacterial loads [2]. These findings suggest that intratumoral microbial burden is not evenly distributed across malignancies, but instead varies according to tumor type and tissue context.

Anatomical location may partly explain this macro‐level heterogeneity. In murine head and neck squamous cell carcinoma (HNSCC) models, Silver et al. showed that intratumoral bacteria accumulated in orthotopic tongue tumors but were absent or minimal in subcutaneous flank tumors [86]. The authors further distinguished the high‐bacterial‐burden oral cavity from low‐bacteria‐burden tumors at nonmucosal sites outside the digestive tract, supporting the idea that intratumoral bacterial burden is strongly influenced by anatomical context. Therefore, tumors arising in or adjacent to microbially enriched anatomical niches may exhibit more prominent microbial signals than tumors from relatively low‐biomass sites.

Importantly, such selectivity may be further refined along anatomical gradients within the same organ. Several studies indicate that right‐sided CRC is more likely to be enriched with oral‐associated anaerobes and pro‐inflammatory taxa, such as *Fusobacterium*, *Prevotella*, and *Peptostreptococcus*, whereas left‐sided CRC more commonly harbors canonical gut commensals, including *Bacteroides* and certain members of *Enterobacteriaceae*/*Escherichia*. This spatial microbial stratification parallels the distinct clinicopathological features and poorer prognosis typically associated with right‐sided CRC [52]. Collectively, these findings underscore that even within a single cancer type, local anatomical and microenvironmental heterogeneity may drive spatial compartmentalization and structural bias of intratumoral microbial communities.

Nevertheless, direct quantitative comparison of microbial load across malignancies remains technically challenging because low‐biomass tumor samples are susceptible to contamination, batch effects, sequencing depth, sample processing, and platform‐specific biases [87]. Thus, current evidence supports a multi‐level heterogeneity of intratumoral microbiota, in which microbial composition and burden vary across tumor types, anatomical niches, and local microenvironmental contexts.

As tumors progress to the metastatic stage, spatially dependent microbial differences remain observable. A large‐scale pan‐cancer analysis encompassing 4160 metastatic tumor biopsies from 26 tissue origins revealed that microbial signals were detectable in the majority of metastatic lesions [32]. Beyond simply cataloging taxa, this metastatic atlas highlighted “metastatic niche‐specific heterogeneity”: microbial load and community diversity varied substantially across metastatic sites and cancer types, with colorectal‐origin metastases exhibiting among the highest diversity, whereas certain head and neck‐derived metastases showed low‐diversity communities dominated by a limited number of taxa. Importantly, the composition of the metastatic microbiome was more strongly associated with the anatomical site of the metastatic biopsy than with the primary tissue of origin, suggesting that organ‐specific ecological constraints may continue to select and shape microbial communities after tumor dissemination. These constraints may include oxygen tension/hypoxia, nutrient and metabolite availability, stromal architecture, and local immune filtering. Meanwhile, tumor‐intrinsic genomic features, such as MSI/MSS status, may further influence microbial clustering patterns in metastatic lesions [32].

Notably, this atlas predominantly profiled metastatic biopsies and therefore could not systematically resolve paired primary‐metastasis differences within the same patient, which remains an important gap for clarifying whether site‐specific metastatic microbiomes arise from (i) “carryover” of microbes from primary tumors, (ii) de novo selection at distant sites, or (iii) both processes in parallel. Nevertheless, these cross‐origin and cross‐site patterns suggest that the local metastatic microenvironment continues to shape intratumoral microbial architecture. Future integration of paired primary‐metastasis sampling, spatial profiling, and route‐of‐dissemination models will be essential to disentangle persistence versus niche selection and to define how site‐specific microbial ecosystems contribute to metastatic outgrowth and therapy response.

Beyond spatial heterogeneity, intratumoral microbial features exhibit reproducible associations with clinical phenotypes. In CRC, elevated abundance of Fn correlates with larger tumor size, deeper invasion, lymph node metastasis, and advanced tumor, node, metastasis (TNM) stage [88]. Similarly, in ESCC and breast cancer, high Fn levels have been associated with increased proliferative activity, enhanced metastatic potential, and unfavorable prognosis [88]. In PDAC, Riquelme et al. demonstrated that tumor microbial diversity is closely linked to immune infiltration patterns and survival outcomes; long‐term survivors exhibited higher relative abundance of taxa such as *Pseudoxanthomonas*, *Streptomyces*, *Saccharopolyspora*, and *Bacillus*, suggesting that intratumoral microbial composition may actively modulate the antitumor immune milieu [89].

Beyond correlative observations, recent studies have provided causal evidence implicating intratumoral bacteria in tumor biology through experimental strategies including microbial depletion/reconstitution, strain supplementation or reintroduction, and mechanistic validation. At the level of the immune microenvironment, PDAC models have shown that microbial depletion or reconstruction can reshape immunosuppressive programs and alter tumor progression [80]. In the metastatic cascade, breast cancer models have demonstrated that supplementation or reintroduction of tumor‐resident intracellular bacteria *Staphylococcus xylosus* enhances metastatic colonization [51]. At the level of oncogenic signaling, in CRC, Fn promotes tumor progression via its adhesin FadA, which activates host signaling pathways through modulation of E‐cadherin/β‐catenin signaling [90], and enhances chemoresistance by regulating autophagy [91]. Regarding therapeutic response, intratumoral *Gammaproteobacteria* in PDAC metabolically inactivate gemcitabine, thereby conferring drug resistance and altering treatment efficacy [82].

Overall, the current body of evidence has evolved from macro‐level descriptions of presence and compositional features to increasingly refined insights into clinical correlations, disease stage‐specific differences, and mechanistic causal validation at the experimental level. Collectively, these findings converge on a central concept: intratumoral bacteria represent biologically active components of the tumor ecosystem, exerting non‐negligible and context‐dependent influences on tumor initiation, progression, metastatic dissemination, and therapeutic responsiveness. To systematically delineate the characteristics and evidentiary strength of intratumoral bacterial signatures, we summarize recent findings across various human cancers (Table S1). This comprehensive overview integrates key parameters, including tumor types, microbial compositional shifts, detection methodologies, clinical cohort sizes, and proposed mechanisms.

## 5 Effects of the Intratumoral Microbiome on Tumor Initiation and Progression

Traditional tumor biology has long regarded microorganisms detected in tumor tissues as incidental “bystanders” or artifacts of experimental contamination, thereby underestimating their biological relevance within the TME. However, with rapid advances in metagenomic sequencing, spatial transcriptomics, and multimodal in situ detection technologies, increasing evidence indicates that microorganisms are widely present across diverse solid tumors and can constitute a relatively stable, functionally engaged “intratumoral ecosystem.” These microbes are not merely passive colonizers; rather, they participate in tumor initiation, growth, and metastatic dissemination through multilayered host–microbe interactions, including induction of genomic instability, reprogramming of host epigenetic states, activation of oncogenic signaling, modulation of immune responses, and metabolic rewiring [6, 7, 92].

### 5.1 Direct Induction of Genomic Instability and DNA Damage

Disruption of genomic integrity is a pivotal early event that drives normal cells toward malignant transformation, and intratumoral microorganisms are increasingly recognized as important contributors to this process. Multiple bacterial species can directly damage host DNA by secreting genotoxins or by triggering oxidative stress. A prototypical example is *Escherichia coli* harboring the polyketide synthase genomic island, which produces the genotoxin colibactin. Colibactin induces DNA double‐strand breaks (DSBs) and generates a characteristic mutational signature consistent with its DNA‐alkylating activity on adenine residues [93]. This mechanism has been strongly linked to early colorectal carcinogenesis [94, 95, 96].

Beyond CRC, intratumoral Fn has also been reported to induce DSBs in a Ku70/p53‐dependent manner, thereby promoting oral squamous cell carcinoma (OSCC) progression [97]. In addition, ETBF produces fragilysin (BFT), and *Campylobacter* spp. produce cytolethal distending toxin (CDT); both can induce oxidative DNA damage and activate DNA damage response programs, thereby increasing the burden on host DNA repair systems. Part of these effects has been linked to upregulation of spermine oxidase and consequent reactive oxygen species (ROS) bursts [98, 99, 100].

Notably, emerging evidence suggests that the impact of tumor‐associated microbes may not be limited to transient toxin exposure. Instead, genotoxin‐producing bacteria that persist and co‐exist with epithelial or tumor cells within locally protected microniches may impose sustained DNA damage pressure and leave recognizable mutational imprints, thereby promoting progressive accumulation of somatic mutations over time [93, 101].

### 5.2 Epigenetic Regulation

Beyond altering the host DNA sequence, intratumoral microorganisms can remodel tumor transcriptional programs through multilayered epigenetic regulation, thereby influencing tumor initiation and progression. With the increasing application of integrated multi‐omics approaches—including metagenomics, transcriptomics, and epigenomics—systematic associations between intratumoral microbial composition and host epigenetic states are being reported with growing frequency. For instance, integrative analyses in hepatocellular carcinoma (HCC) have linked intratumoral microbial features to host DNA methylation patterns and transcriptional alterations, suggesting that tumor‐associated microorganisms may participate in early tumorigenesis by reshaping epigenetic regulatory networks [102].

In CRC, intratumoral Fn has been associated with CpG island hypermethylation and CpG island methylator phenotype (CIMP) positivity, implicating microbe‐linked hypermethylation events at tumor suppressor–related loci [103, 104]. Moreover, in lung adenocarcinoma, tumor‐associated microbial communities have been reported to be enriched for bacteria harboring methionine biosynthesis pathways (e.g., members of *Rhizobiales*), raising the possibility that increased methionine and methyl‐donor availability may support tumor methylation capacity and epigenetic reprogramming [105].

In addition to DNA methylation and histone modifications, microbial effects may extend to post‐transcriptional epigenetic regulation. Notably, Fn has been implicated in dysregulation of the N6‐methyladenosine (m6A) machinery in CRC, including methyltransferase‐like 3 (METTL3)‐associated pathways and downstream changes in transcript stability and oncogenic programs, thereby expanding the scope of microbe‐driven epigenetic remodeling beyond canonical DNA‐ and histone‐centered mechanisms [106].

### 5.3 Activation and Maintenance of Oncogenic Signaling Pathways

Beyond inducing genetic damage and chronic inflammatory stress, intratumoral microorganisms can directly modulate multiple oncogenic signaling pathways that govern tumor proliferation, survival, and dissemination. Such regulation often converges on key nodal circuits, thereby reinforcing malignant phenotypes and helping tumor cells maintain signaling homeostasis.

#### 5.3.1 Wnt/β‐Catenin Signaling

The Wnt/β‐catenin pathway is aberrantly activated in many solid tumors, and diverse microorganisms can regulate this axis by perturbing adhesion molecules and β‐catenin turnover. In the context of *H. pylori*, CagA has been reported to interfere with β‐catenin degradation and promote its nuclear accumulation [107, 108]. In CRC, Fn employs its adhesin FadA to bind E‐cadherin, facilitating β‐catenin release and nuclear translocation. Similarly, ETBF‐derived BFT can activate β‐catenin signaling via E‐cadherin cleavage [90]. Collectively, these mechanisms can amplify Wnt signaling and promote tumor growth and progression.

#### 5.3.2 PI3K‐AKT Signaling

The PI3K‐AKT pathway is a central hub regulating cellular growth, survival, and metabolism, and is frequently constitutively activated across cancers [109, 110, 111]. In lung cancer, integrative analyses combining lower airway microbiome profiling with host transcriptomics have shown that enrichment of oral commensals (e.g., *Streptococcus* and *Veillonella*) is significantly associated with upregulation of PI3K signaling. Consistently, exposure of airway epithelial cells to relevant microbial communities in vitro recapitulates a similar pathway activation pattern, providing more direct support for a “microbial exposure‐oncogenic signaling activation” link [112]. In oral cancer, *Porphyromonas gingivalis* can elicit TLR‐associated inflammatory responses and upregulate cytokines such as IL‐6, IL‐8, and TNF‐α; in parallel, its virulence factors and host responses may cooperatively engage PI3K/AKT signaling (often accompanied by ERK/MAPK programs), thereby enhancing tumor cell survival, migration, and invasion [113].

#### 5.3.3 Mapk/Erk Signaling

The mitogen‐activated protein kinase/extracellular signal‐regulated kinase (MAPK/ERK) pathway converts extracellular cues into pro‐proliferative and anti‐apoptotic outputs [114, 115]. In CRC, Fn can activate TLR4/MYD88 signaling and induce miR‐21, thereby relieving repression of the RAS–ERK cascade and enhancing tumor cell proliferation and invasiveness [91]. In addition, intracellular invasion by *P. gingivalis* has been reported to activate MAPK/ERK signaling through gingipains and related virulence determinants, potentially supporting pro‐tumorigenic phenotypes [116, 117].

#### 5.3.4 STING Signaling

The cyclic GMP‐AMP synthase‐stimulator of interferon (IFN) genes (cGAS‐STING) pathway senses aberrant cytosolic double‐stranded DNA (dsDNA)—originating from the genome, mitochondria, or microorganisms—generates cGAMP, and activates STING to initiate innate immune responses [118]. In the context of tumor immunity, STING activation can enhance antigen presentation and promote antitumor immune priming. Notably, in CD47‐based immunotherapy models, systemic or local administration of *Bifidobacterium* promotes intratumoral accumulation and activates STING in an IFN‐dependent manner, converting nonresponders into responders and enhancing therapeutic efficacy [119].

### 5.4 Chronic Inflammation

Chronic inflammation is a hallmark of tumor initiation and progression, and intratumoral microorganisms can serve as persistent triggers by continuously engaging innate immune sensing pathways within the TME [120]. Microbial components and metabolites are recognized by pattern‐recognition receptors (PRRs), particularly Toll‐like receptors (TLRs), thereby initiating an NF‐κB‐centered inflammatory cascade. This program drives the production of pro‐inflammatory cytokines (e.g., IL‐6, IL‐8, TNF‐α, and IL‐17) and chemokines, ultimately creating a local milieu that supports proliferation, survival, and resistance to apoptosis [121]. For example, Fn activates NF‐κB via the TLR4/MYD88 axis, upregulates inflammatory mediators, and induces miR‐21 to suppress RASA1, thereby reinforcing RAS signaling and promoting tumor growth. Infection is also associated with increased Th17‐related cytokines (e.g., IL‐17 and IL‐21), further amplifying inflammatory circuits [122]. Similarly, ETBF‐secreted BFT cleaves E‐cadherin and concomitantly activates both β‐catenin and NF‐κB signaling, thereby driving colonic inflammation and carcinogenesis [123, 124].

Importantly, microbe‐triggered inflammation is not a static process. Sustained inflammatory signaling can compromise epithelial barrier integrity, facilitate translocation of microbial products, and establish self‐reinforcing feedback loops. Moreover, inflammation driven by microorganisms intersects tightly with multiple oncogenic mechanisms. On the one hand, ROS produced by immune cells during inflammatory responses can directly induce DNA damage and promote genomic instability [125]. On the other hand, persistent activation of inflammatory cytokine axes‐such as IL‐6/JAK/STAT3‐can couple with tumor cell survival programs, strengthening anti‐apoptotic capacity and adaptive growth. In addition, bacteria such as *P. gingivalis* can, within an inflammatory context, further activate PI3K/AKT or JAK/STAT signaling [113, 126] and cooperate with NF‐κB to suppress apoptosis and promote epithelial–mesenchymal transition (EMT), thereby translating inflammatory signals into more invasive tumor phenotypes.

## 6 Role of the Intratumoral Microbiota in Tumor Metastasis

**FIGURE 3:**
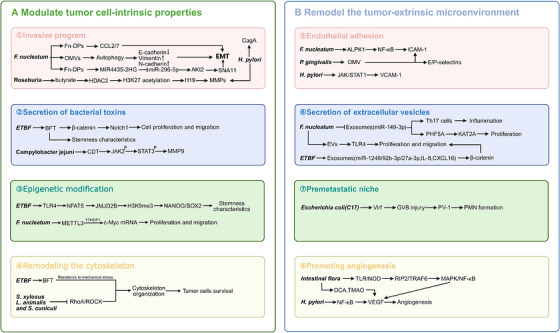
Mechanisms by which tumor‐resident bacteria promote tumor metastasis. **(A)** Tumor‐resident bacteria promote tumor metastasis by modulating the intrinsic properties of tumor cells. (1) Invasive program: Bacterial infection activates signaling cascades (e.g., SNAI1 and Akt2) that drive EMT and upregulate MMPs (e.g., MMP2 and MMP9), facilitating extracellular matrix degradation and enhancing tumor cell invasiveness. (2) Secretion of bacterial toxins: Bacterial toxins (e.g., BFT from ETBF, CDT from *Campylobacter jejuni*) activate oncogenic pathways (such as Notch1, β‐catenin, and JAK2/STAT3), conferring stemness characteristics and promoting proliferation and migration. (3) Epigenetic modification: Intratumoral bacteria upregulate epigenetic regulators (e.g., JMJD2B and METTL3) to alter histone modifications (H3K9me3) or mRNA methylation (YTHDF1), thereby promoting the expression of pluripotency and oncogenic factors (NANOG/SOX2, c‐Myc). (4) Remodeling the cytoskeleton: Specific bacteria (e.g., ETBF, *S. xylosus*, *L. animalis*, and *S. cuniculi*) reshape cytoskeletal structures via the RhoA‐ROCK pathway, enhancing tumor cell resistance to mechanical stress and facilitating survival during dissemination. **(B)** Tumor‐resident bacteria promote tumor metastasis by remodeling the tumor‐extrinsic microenvironment. (1) Endothelial adhesion: Intratumoral bacteria (e.g., *F. nucleatum*, *P. gingivalis*, and *H. pylori*) induce the expression of endothelial adhesion molecules (such as ICAM‐1, VCAM‐1, and E‐/P‐selectins) via pathways like ALPK1/NF‐κB and JAK/STAT1, facilitating tumor cell adherence and vascular extravasation. (2) Secretion of extracellular vesicles: Bacterial infection stimulates the release of EVs and exosomes enriched with specific microRNAs (e.g., miR‐1246, miR‐92b‐3p) that mediate inflammation, Th17 cell activation, and enhanced proliferation and migration via TLR4 signaling. (3) PMN: Specific strains (e.g., *E. coli* C17) secrete VirF that disrupt the GVB through PV‐1 alteration, driving the formation of a PMN. (4) Promoting angiogenesis: Bacterial metabolites (e.g., DCA and TMAO) and intestinal flora components activate TLR/NOD and RIP2/TRAF6 signaling, culminating in MAPK/NF‐κB and VEGF pathway activation to stimulate angiogenesis. β‐catenin, beta‐catenin; Akt, AKT serine/threonine kinase; ALPK1, alpha kinase 1; BFT, *Bacteroides fragilis* toxin; CDT, cytolethal distending toxin; c‐Myc, MYC proto‐oncogene; DCA, deoxycholic acid; *E. coli C17*, *Escherichia coli strain C17*; EMT, epithelial‐mesenchymal transition; H3K9me3, histone H3 lysine 9 trimethylation; ICAM‐1, intercellular adhesion molecule 1; JAK, Janus kinase; JMJD2B, Jumonji domain‐containing protein 2B; MAPK, mitogen‐activated protein kinase; METTL3, methyltransferase‐like 3; MMPs, matrix metalloproteinases; NOD, nucleotide‐binding oligomerization domain‐containing protein; ETBF, enterotoxigenic *Bacteroides fragilis*; E‐/P‐selectins, E‐selectin and P‐selectin; EVs, extracellular vesicles; GVB, gut vascular barrier; NANOG, Nanog homeobox; NF‐κB, nuclear factor kappa‐B; Notch1, Notch receptor 1; PMN, pre‐metastatic niche; PV‐1, plasmalemma vesicle‐associated protein 1; RIP2, receptor‐interacting serine/threonine kinase 2; STAT, signal transducer and activator of transcription; SNAI1, Snail family transcriptional repressor 1; SOX2, SRY‐box transcription factor 2; TLR, Toll‐like receptor; TMAO, trimethylamine N‐oxide; TRAF6, TNF receptor‐associated factor 6; VCAM‐1, vascular cell adhesion molecule 1; VEGF, vascular endothelial growth factor; VirF, virulence factor F; YTHDF1, YTH N6‐methyladenosine RNA binding protein 1.

**FIGURE 4:**
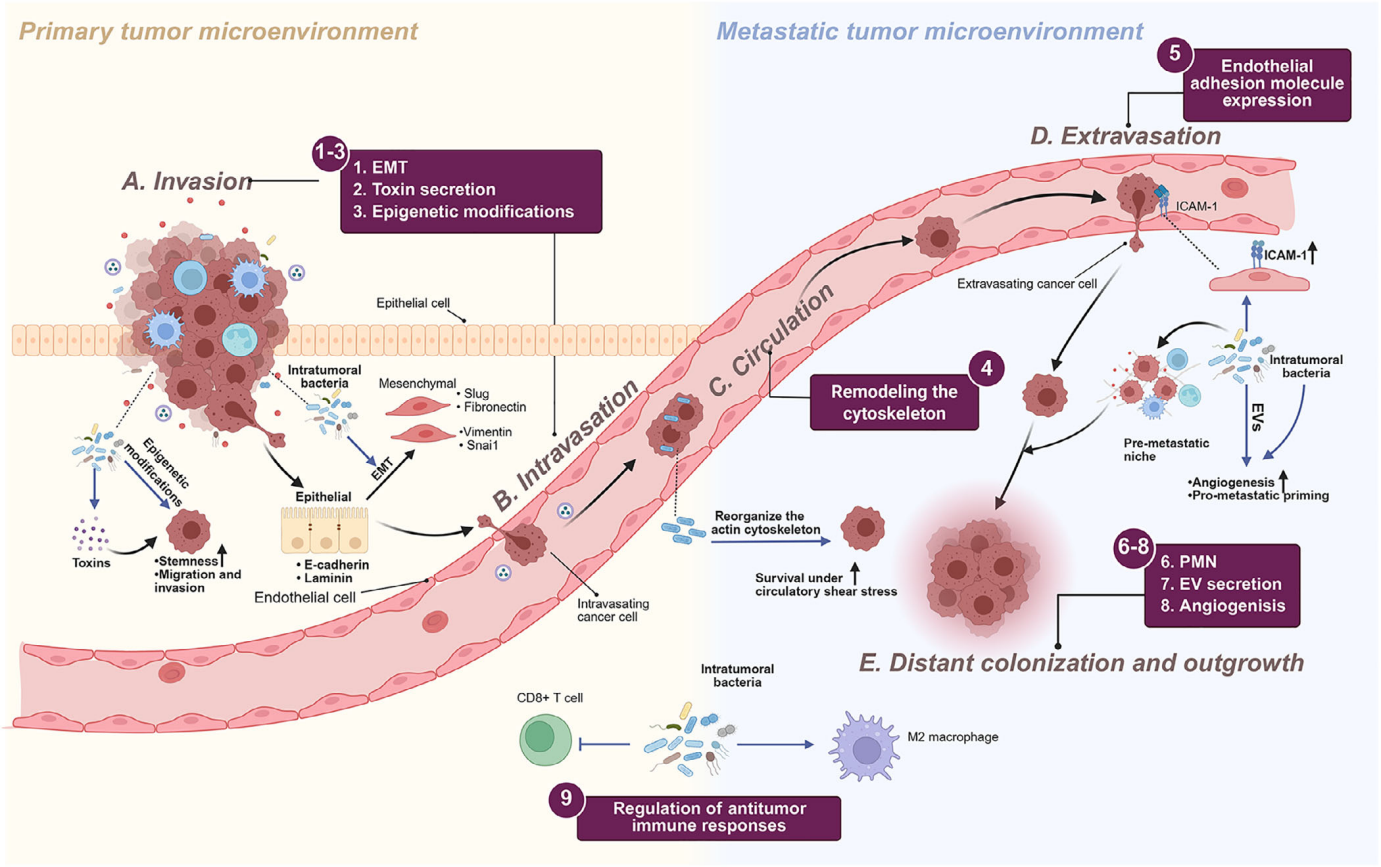
Spatiotemporal roles of intratumoral bacteria throughout the tumor metastatic cascade. Intratumoral bacteria accompany tumor cells and actively participate in multiple critical steps of metastasis. (a and b) Local invasion and intravasation (Mechanisms 1–3): At the primary tumor site, bacteria drive the EMT program by upregulating mesenchymal markers (e.g., Slug, Fibronectin, Vimentin, and Snai1) and suppressing epithelial markers (e.g., E‐cadherin and laminin). Concurrently, bacteria‐derived toxin secretion and subsequent epigenetic modifications collectively enhance tumor cell stemness, migration, and invasive capacities. (c) Circulation (Mechanism 4): During hematogenous transit, intracellular bacteria remodel the actin cytoskeleton of CTCs, conferring crucial resistance to circulatory shear stress and promoting intravascular survival. (d) Extravasation (Mechanism 5): Bacteria facilitate the arrest and transendothelial migration of tumor cells by inducing the expression of endothelial adhesion molecules, such as ICAM‐1, on the distant vascular endothelium. (e) Distant colonization and outgrowth (Mechanisms 6–8): At secondary organs, bacteria drive the establishment of a PMN via the secretion of EVs, which prime the local microenvironment and stimulate angiogenesis. Furthermore, intratumoral bacteria actively regulate the local immune microenvironment by suppressing cytotoxic CD8+ T cells and promoting immunosuppressive M2 macrophage polarization, thereby establishing a hospitable niche that supports distant metastatic outgrowth (Mechanisms 9). CD8, cluster of differentiation 8; CTCs, circulating tumor cells; E‐cadherin, epithelial cadherin; EMT, epithelial‐mesenchymal transition; EVs, extracellular vesicles; ICAM‐1, intercellular adhesion molecule 1; M2, M2‐polarized macrophages (alternatively activated macrophages); PMN, pre‐metastatic niche; Snai1, Snail family transcriptional repressor 1.

The metastatic cascade comprises four key stages: EMT‐driven initiation of migration, matrix metalloproteinase (MMP)‐mediated invasion, survival of CTCs during dissemination, and distal colonization facilitated by premetastatic microenvironmental reprogramming [16, 127, 128]. Across these steps, immune evasion is a prerequisite for metastatic success: disseminating tumor cells must escape cytotoxic surveillance by dampening antigen presentation and effector T‐cell trafficking, engaging inhibitory checkpoint programs, and fostering immunosuppressive niches that support CTC persistence and outgrowth at distant sites. Emerging evidence further indicates that, as integral components of the TME, intratumoral microbial communities not only contribute to the establishment of local immunosuppressive ecosystems but also actively drive metastatic progression primarily through two complementary molecular dimensions: (i) reprogramming tumor cell‐intrinsic properties and (ii) remodeling tumor cell‐extrinsic microenvironmental cues (Figure 3) [1, 6, 7, 16]. In this section, we systematically reorganize current evidence by mapping these microbiota‐driven mechanisms onto the macroscopic temporal and spatial framework of the metastatic cascade, comprehensively elucidating their specific spatiotemporal roles during local invasion, intravasation, survival in circulation, and extravasation with distal colonization (Figure 4).

### 6.1 Local Invasion and Intravasation

#### 6.1.1 EMT‐Driven Tumor Invasion and Metastatic Competence

Tumor cells acquire invasive and metastatic potential through coordinated molecular reprogramming that enhances motility, extracellular matrix (ECM) degradation, and transendothelial migration. A central event in this process is EMT, during which epithelial tumor cells lose cell–cell adhesion and polarity while gaining mesenchymal and stem cell‐like properties, enabling detachment from the primary tumor and invasion into surrounding tissues and vasculature [129, 130]. Accumulating evidence indicates that IM actively participate in this invasive reprogramming by inducing EMT and by upregulating invasion‐associated effector molecules.

Multiple tumor‐associated bacteria have been shown to directly trigger EMT through diverse signaling pathways. The virulence protein Fn‐Dps derived from Fn induces EMT by upregulating the chemokines CCL2 and CCL7, thereby promoting invasion and metastasis of CRC cells in vivo [131]. Kong et al. demonstrated that Fn activates the TLR4/Keap1/NRF2 axis, leading to increased expression of CYP2J2 and its downstream metabolite 12,13‐EpOME, which in turn drives EMT and metastatic progression in CRC [132]. In OSCC, *Clostridium perfringens* drives tumor metastasis by activating the lncRNA MIR4435‐2HG/miR‐296‐5p/Akt2/SNAI1 axis, which orchestrates the EMT program through the specific downregulation of E‐cadherin and concurrent upregulation of mesenchymal markers (N‐cadherin and vimentin) [133]. Similarly, in laryngeal squamous cell carcinoma, *C. perfringens* promoted EMT by suppressing the expression of TGFBR2. In oral cancer, Fn‐secreted outer membrane vesicles (OMVs) further enhanced cancer cell migration and invasion [134]. In addition, *H. pylori* induces EMT‐like changes in gastric epithelial cells, via the oncoprotein CagA, accompanied by enhanced migration, invasion and tumor spheroid formation, as well as increased expression of CD44, a gastric cancer stem cell marker [135].

Beyond transcriptional reprogramming associated with EMT, intratumoral bacteria also enhance tumor invasiveness by upregulating proteins directly involved in ECM degradation and cell–cell or cell–endothelium interactions. MMPs are key mediators of ECM remodeling and basement membrane degradation and play essential roles in tumor invasion, EMT progression, immune regulation, and angiogenesis [136]. Ou et al. reported that infection of CRC cells with Fn significantly upregulated MMP7 expression [137]. In gastric cancer, *H. pylori* markedly increased the enzymatic activity of MMP‐2 and MMP‐9 by approximately threefold and 19‐fold, respectively, while concomitant macrophage infiltration synergized with MMP‐9 to promote basement membrane degradation [138]. Moreover, butyric acid produced by butyrate‐producing bacteria within the tumor microbiota (e.g., *Rochesteria* spp.) can inhibit histone deacetylase 2 (HDAC2) expression, reduce its binding to the H19 promoter, increase H3K27 acetylation, and upregulate H19 expression, thereby inducing MMP15 and other MMPs and enhancing tumor cell migration and invasion [139].

Collectively, these studies indicate that intratumoral microbiota enhance tumor cell invasive and metastatic competence by two steps: initiating EMT‐driven phenotypic reprogramming and reinforcing invasion through upregulation of MMPs and adhesion molecules.

#### 6.1.2 Toxin Secretion Promoting Metastasis

Certain bacteria produce specific toxins that promote tumor cell migration. While *Bacteroid fragilis* is normally found in the mammary gland, colon, and oral cavity and contributes to the maintenance of immune homeostasis, ETBF significantly enhances the metastatic ability of breast cancer cells. BFT secreted by ETBF alters the morphology and function of normal mammary epithelium and breast cancer cells, such as enhancing membrane vesiculation, migratory invasiveness, and increasing stem cell‐like properties [140]. ETBF‐infected tumor cells have enhanced migratory and invasive abilities, increased β‐catenin expression, and activated Notch1 pathway through toxin‐induced mechanisms, highlighting the role of ETBF in promoting migration and secondary colonization of breast cancer cells [140]. In studies of CRC patients, the CDT produced by *Campylobacter jejuni* has been found to promote tumor metastasis through activation of the JAK2‐STAT3‐MMP9 signaling pathway, and inhibition of JAK2 phosphorylation or MMP9 expression significantly inhibited CDT‐induced CRC cell migration, invasion, and metastasis [141].

#### 6.1.3 Modification of Epigenetic Inheritance

In a mouse model of CRC, ETBF has been shown to enhance the stemness of CRC cells both in vitro and in vivo, as evidenced by the upregulation of core stemness genes such as NANOG and SOX2 [142]. The underlying mechanism involves epigenetic reprogramming: ETBF infection activates the TLR4/NFAT5 signaling axis, leading to increased expression of the demethylase JMJD2B. JMJD2B, in turn, specifically catalyzes the demethylation of histone H3K9me3 at the NANOG promoter, thereby relieving transcriptional repression and sustaining enhanced stemness and self‐renewal capacity of the cancer cells. Clinical correlation analyses further support this mechanism, as the abundance of ETBF in human CRC samples shows a significant positive correlation with the expression levels of NFAT5, JMJD2B, and NANOG [142]. Beyond ETBF, other intratumoral bacteria can also drive tumor metastasis through epigenetic mechanisms. For example, research by Guo et al. demonstrated that Fn can invade various tumor cell types, including ESCC cells, and significantly upregulate the expression of METTL3 [143]. Mechanistically, intracellular Fn infection promotes the transcription of METTL3. The encoded m^6^A methyltransferase then, in a YTHDF1‐dependent manner, specifically catalyzes methylation in the 3′‐UTR region of the proto‐oncogene c‐Myc mRNA, thereby enhancing its stability and protein expression, ultimately driving ESCC proliferation and metastasis [143]. This study not only reveals the carcinogenic role of Fn in ESCC and its epigenetic regulatory mechanism but also identifies a potential novel molecular target for the treatment of this disease.

### 6.2 Survival in Circulation

Cytoskeletal remodeling is one of the key mechanisms by which intratumoral bacteria enhance the survival of CTCs in the bloodstream. A study has shown that intratumoral bacteria can directly suppress the RhoA/ROCK pathway and drive actin cytoskeletal remodeling in CTCs, characterized by reduced stress‐fiber intensity, cortical actin reorganization, and global actin network rewiring. These changes, in turn, reshape cell morphology and adhesion properties and enhance mechanical stability [51]. Functionally, such remodeling confers greater mechanical fitness to CTCs, enabling them to better withstand fluid shear stress and deformation forces in the bloodstream, thereby markedly improving survival during circulation and facilitating subsequent hematogenous dissemination and metastatic seeding at distant organs [51].

Importantly, this pro‐metastatic effect appears to be relatively independent: experiments in germ‐free mice exclude confounding contributions from the intestinal microbiota, while immunodeficient models further support that the phenotype does not strictly rely on adaptive or innate immune regulation, collectively indicating that intracellular bacteria can promote metastasis largely by directly reprogramming the mechanical phenotype of tumor cells. Consistent with this concept, Parida et al. reported that enterotoxin‐producing *Pseudomonas fragilis* can similarly induce cytoskeletal remodeling in breast cancer cells via bacterial toxins [140]. Together, these findings suggest that an “intratumoral bacteria‐RhoA/ROCK signaling‐cytoskeletal remodeling‐shear‐stress tolerance” axis may represent one recurring mode of action among metastasis‐associated bacteria, although its generalizability across bacterial species remains to be further established [72, 74].

### 6.3 Extravasation and Distal Colonization Regulation

#### 6.3.1 Endothelial Adhesion and Extravasation

In addition to ECM remodeling, tumor‐associated bacteria facilitate metastatic dissemination by increasing the expression of adhesion molecules that promote tumor cell extravasation. Zhang et al. demonstrated that Fn enhances the adhesion of CRC cells to endothelial cells by inducing ICAM1 expression, thereby promoting extravasation and metastasis [144]. Mechanistically, Fn activated the specific pattern recognition receptor ALPK1, which triggered NF‐κB signaling and subsequent ICAM1 upregulation. Consequently, the abundance of Fn in CRC tumor tissues was positively correlated with ALPK1 and ICAM1 expression levels, and high expression of either molecule was significantly associated with shorter overall survival in CRC patients [144].

#### 6.3.2 Extracellular Vesicles (EVs)

Recent studies have shown that specific bacteria colonizing the tumor can drive the metastatic process by regulating the secretion of pro‐metastatic EVs by cancer cells, which can promote tumor metastasis in multiple dimensions by delivering microRNAs and functional proteins to distal normal tissues. Key mechanisms include remodeling the metastatic microenvironment [145]. Involvement of intratumoral bacteria in EV‐mediated metastasis include the following: (1) ETBF activates the PHF5A‐dependent KAT2A RNA variable splicing pathway in CRC cells by inhibiting the release of miR‐149‐3p in the exocytosis body, which in turn significantly enhances the proliferation of tumor cells [146]. (2) Fn infection induces CRC cells to secrete a population of exosomes enriched with miR‐1246/92b‐3p/27a‐3p and CXCL16/RhoA/IL‐8, which deliver pro‐metastatic signals to the uninfected cells through the paracrine pathway, ultimately leading to the recipient cells acquiring an invasive phenotype [147]. (3) *Clostridium difficile*‐derived EVs enhance the sustained activation of TLR4 in tumor cells through TLR4 that can significantly upregulate the release of immune‐suppressive exosomes, and the molecules carried by these vesicles, such as PD‐L1, TGF‐β, and others, can assist the tumor cells to achieve immune escape and promote the metastatic foci formation by negatively modulating the T‐cell function [148]. Together, these studies reveal the pivotal role of the “microbe‐exosome‐host” interaction network in tumor metastasis, suggesting that targeting the EVs secretion pathway regulated by pathogenic bacteria may become a breakthrough therapeutic strategy.

#### 6.3.3 Premetastatic Niche (PMN) Establishment

Studies have shown that the intratumoral microbiota of CRC can provide microenvironmental support for CRC metastasis by disrupting the integrity of the gut vascular barrier (GVB), which leads to the migration of the microbiota to the liver via the portal vein system, and the formation of PMN [149]. Notably, this mechanism was further elucidated in a study by Bertocchi et al. Their team confirmed through mechanistic studies that the CRC‐associated *Escherichia coli* C17 strain can directly promote the PMN through the Type III Secretion System (TTSS)‐dependent pathway by utilizing the virulence effector virulence factor F (VirF) directly destroys the structural integrity of GVB, which promotes bacterial translocation to the liver parenchyma, ultimately driving the maturation of PMN and significantly enhancing the ability of metastasis formation [150]. The above findings systematically reveal the cascading pathological mechanism of “intratumoral flora‐GVB destruction‐hepatic PMN formation,” suggesting that maintaining the homeostatic state of GVB and prevention of gut microbiota translocation to the PMN may be a novel mechanism to block CRC metastasis.

#### 6.3.4 Angiogenesis and Outgrowth

Angiogenesis is a fundamental biological process supporting tumor growth and metastatic outgrowth. Emerging evidence indicates that specific microorganisms, both within the TME and from associated mucosal niches, can directly or indirectly modulate this process. A prominent example is *H. pylori*, which directly promotes angiogenesis in gastric carcinogenesis. *H. pylori* activates host MEK/ERK and NF‐κB signaling pathways, leading to enhanced transcription of vascular endothelial growth factor A (VEGF‐A) and other pro‐angiogenic factors. In vivo studies corroborate that chronic colonization induces sustained upregulation of VEGF‐A and angiopoietin‐2, driving neovascularization during early tumorigenesis [151, 152, 153]. Beyond direct local effects, distant microbiota can also shape a pro‐angiogenic environment. For instance, ETBF in the gut induces the expression of the key angiogenic cytokine IL‐8 in CRC cells via STAT3 signaling, thereby fostering a favorable microenvironment for vascular growth [154]. Furthermore, gut dysbiosis triggered by factors such as a high‐fat diet can lead to the accumulation of metabolites like deoxycholic acid (DCA), which activates the VEGFR2 pathway and promotes angiogenic mimicry, accelerating tumor progression [153, 155].

Collectively, these findings underscore that specific tumor‐associated or mucosa‐associated microbes, through well‐defined pro‐angiogenic signaling pathways (e.g., VEGF, STAT3, and NF‐κB), play a crucial role in promoting tumor angiogenesis, thereby facilitating both local growth and distant metastatic outgrowth.

### 6.4 Intratumoral Bacteria Induced Immune Remodeling in the Metastatic Cascade

Immune remodeling is not an isolated step in the metastatic cascade but rather a critical regulatory layer that operates throughout the entire metastatic process. As an integral component of the TME, intratumoral bacteria can promote the establishment of an immunosuppressive milieu and thereby facilitate metastasis. In a mouse gastric cancer model, intratumoral Fn was shown to be internalized by tumor cells and to trigger an IL‐17‐NF‐κB‐associated inflammatory program, which drives the recruitment of tumor‐associated neutrophils (TANs) and their polarization toward a PD‐L1–high immunosuppressive phenotype. These remodeled neutrophils suppress CD8^+^ T‐cell effector function via the PD‐1/PD‐L1 axis, ultimately promoting metastatic progression [156]. Similarly, in a mouse liver tumor model, *Enterococcus faecalis* and *Streptococcus anginosus* enriched in intrahepatic metastatic hepatocellular carcinoma (IM‐HCC) enhanced immunosuppressive features, characterized by increased myeloid‐derived suppressor cells (MDSCs) and reduced CD8^+^ T‐cell activity and IFN‐γ production, thereby promoting invasion and metastasis [157]. During the circulation phase, neutrophils can form complexes with CTCs, improving their survival in the bloodstream and increasing metastatic potential [158, 159]; neutrophil extracellular traps (NETs) induced by microbe‐associated components have also been implicated in metastasis [160]. In addition, intratumoral bacteria may influence metastatic outcomes through immunomodulatory metabolism; for example, *S. anginosus*‐mediated conversion of arginine to ornithine has been associated with impaired CD8^+^ T‐cell function [161]. Collectively, current evidence suggests that intratumoral bacteria can shape a metastasis‐permissive immune ecosystem through immune‐cell reprogramming, immune interactions during circulation, and immunometabolic regulation; the underlying mechanisms will be discussed in the next section.

## 7 Mechanisms by Which Intratumoral Bacteria Regulate Tumor Progression via Crosstalk With the Immune Microenvironment

**FIGURE 5:**
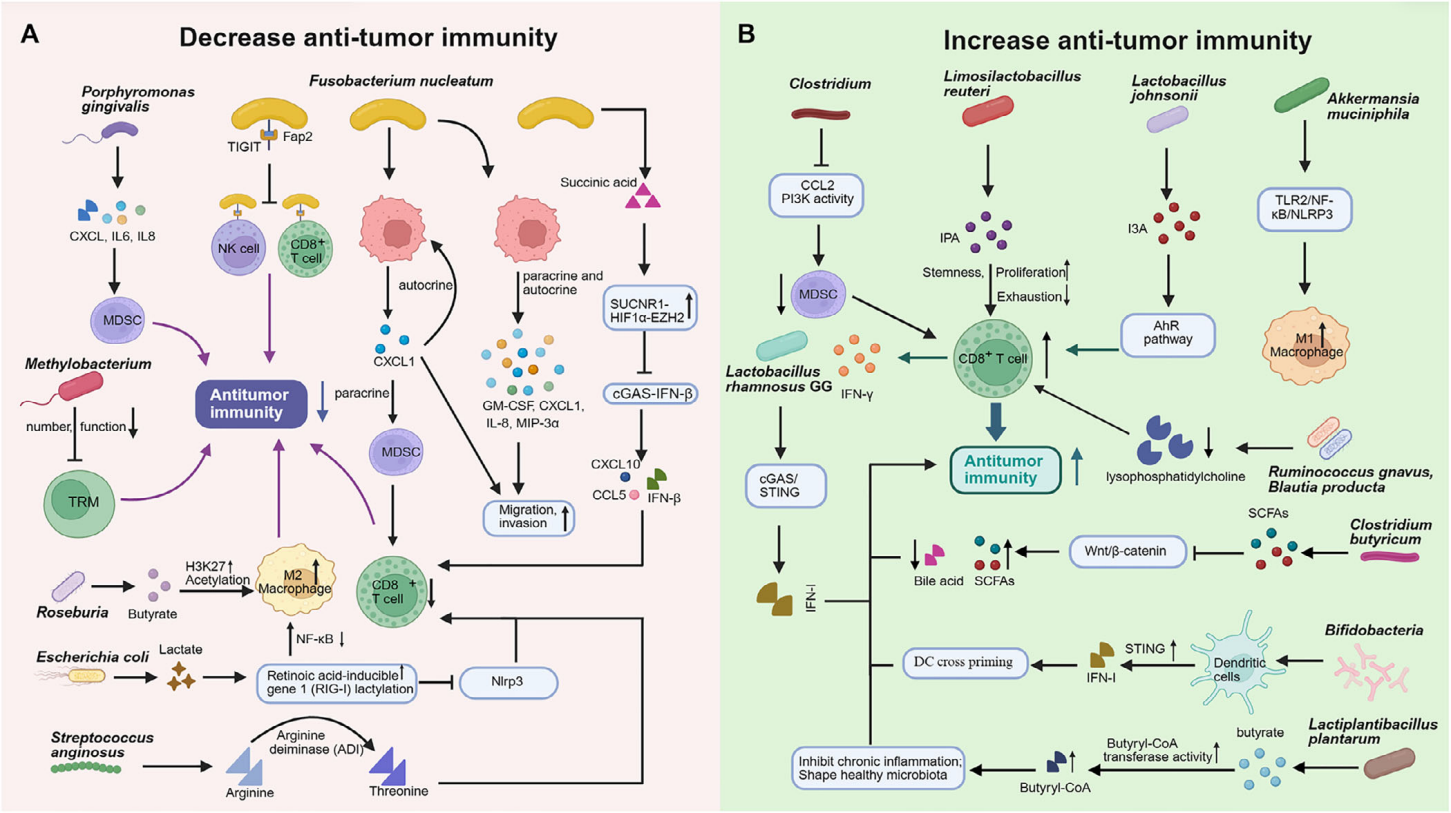
Bidirectional immunoregulatory effects of intratumoral bacteria on tumors. **(A)** Decreasing anti‐tumor immunity: Intratumoral bacteria (e.g., Fn, *Porphyromonas gingivalis*, *Streptococcus anginosus*, *Methylobacterium*, *Roseburia*, and *Escherichia coli*) suppress anti‐tumor immunity by establishing an immunosuppressive microenvironment. They achieve this through several key mechanisms: (1) Recruiting and polarizing immunosuppressive cells: Secreting chemokines (e.g., CXCL1, IL‐6, and IL‐8) to recruit MDSCs, and utilizing bacterial metabolites (e.g., *Roseburia*‐derived butyrate promoting H3K27 acetylation and *E. coli*‐derived lactate inducing RIG‐I lactylation) to drive M2 macrophage polarization. (2) Directly exhausting cytotoxic lymphocytes: Engaging inhibitory immune checkpoints (e.g., Fn surface protein Fap2 binding to TIGIT on NK and CD8+ T cells), impairing TRMs (e.g., *Methylobacterium*), and depleting essential amino acids (e.g., *S. anginosus* ADI depleting arginine) to directly blunt CD8+ T cell and NK cell cytotoxicity. (3) Reprogramming inflammatory and metabolic cascades: For instance, Fn‐derived succinate activates the SUCNR1‐HIF1α‐EZH2 axis, which alters cGAS‐IFN‐β signaling and downstream chemokines (CXCL10/CCL5) to further exclude CD8+ T cells and promote tumor migration and invasion. **(B)** Increasing anti‐tumor immunity: Conversely, specific beneficial intratumoral bacteria (e.g., *Clostridium*, *Lactobacillus* species, *A. muciniphila*, *Bifidobacteria*, and *C. butyricum*) actively enhance anti‐tumor responses. These microbes orchestrate immune activation through distinct pathways: (1) Secreting specific immune‐modulatory metabolites (e.g., IPA, I3A, SCFAs/butyrate, and lysophosphatidylcholine) that directly boost CD8+ T cell stemness and proliferation via the AhR pathway. (2) Activating the cGAS/STING and TLR2/NF‐κB pathways to stimulate DC cross‐priming, IFN‐I production, and M1 macrophage polarization, and (3) mitigating local immunosuppressive networks by inhibiting CCL2/PI3K activity (thereby reducing MDSC accumulation) and suppressing Wnt/β‐catenin signaling. The overall effect of intratumoral bacteria is therefore bidirectional and context‐dependent, delicately balancing immune evasion and immune activation within the TME. ADI, arginine deiminase; AhR, aryl hydrocarbon receptor; CCL, C‐C motif chemokine ligand (CCL2, CCL5); cGAS, cyclic GMP‐AMP synthase; CXCL, C‐X‐C motif chemokine ligand (CXCL1, CXCL10); DCs, dendritic cells; EMT, epithelial‐mesenchymal transition; EZH2, enhancer of zeste homolog 2; Fn, *Fusobacterium nucleatum*; Fap2, *Fusobacterium* autotransporter protein 2; H3K27, histone H3 lysine 27; HIF1α, hypoxia‐inducible factor 1‐alpha; I3A, indole‐3‐aldehyde; IFN‐β, interferon‐beta; IFN‐I, type I interferon; IL, interleukin (IL‐6, IL‐8); IPA, indole‐3‐propionic acid; MDSCs, myeloid‐derived suppressor cells; NF‐κB, nuclear factor kappa‐B; NK cells, natural killer cells; PI3K, phosphoinositide 3‐kinase; RIG‐I, retinoic acid‐inducible gene I; SCFAs, short‐chain fatty acids; STING, stimulator of interferon genes; SUCNR1, succinate receptor 1; TIGIT, T cell immunoreceptor with Ig and ITIM domains; TLR2, Toll‐like receptor 2; TME, tumor microenvironment; TRMs, tissue‐resident memory T cells; Wnt, wingless‐type MMTV integration site family; β‐catenin, beta‐catenin.

Intratumoral bacteria can reshape tumor progression not only by acting on malignant cells but also by engaging in extensive bidirectional crosstalk with the immune microenvironment. As spatially organized constituents of the tumor ecosystem, these microbes and their effectors/metabolites influence immune‐cell recruitment, differentiation, and functional polarization, thereby tilting the balance between immune surveillance and immune escape. Mechanistically, intratumoral bacteria regulate antitumor immunity through three interconnected layers: (i) direct modulation of immune effector and suppressor cell compartments, (ii) metabolic rewiring that alters immune activation thresholds and cytokine/chemokine networks, and (iii) broader stromal interactions that secondarily condition immune responses. These immune‐centric mechanisms are summarized in Figure 5, highlighting both immunosuppressive and immune‐activating pathways through which intratumoral bacteria shape tumor immunity and, consequently, tumor progression.

### 7.1 Immune Parenchymal Components: Immune Cells Modulated by Intratumoral Bacteria

#### 7.1.1 Immune Suppression Induced by Intratumoral Bacteria

Available evidence indicates that intratumoral bacteria are broadly distributed within and around malignant cells as well as multiple immune cell populations. This spatial organization enables them to both directly influence tumor cell proliferation and metastatic traits and, in parallel, drive profound remodeling of the immune microenvironment [1]. Overall, a growing body of work suggests that the intratumoral microbiome can markedly attenuate antitumor immunity through cytokine‐ and metabolism‐dependent mechanisms (Figure 5A).

Fn represents a prototypical example of multidimensional immunosuppression. Mechanistically, Fn exploits its surface protein Fap2 to bind TIGIT, an inhibitory immune checkpoint receptor, thereby blunting natural killer cell cytotoxicity and impairing CD8^+^ T‐cell activation to facilitate immune evasion [162]. Udayasuryan et al. further reported that Fn‐infected PDAC cells secrete a pro‐tumorigenic cytokine mixture—such as granulocyte‐macrophage colony‐stimulating factor (GM‐CSF), CXCL1, IL‐8, and macrophage inflammatory protein‐3α (MIP‐3α)—through autocrine and paracrine signaling, thereby enhancing metastasis‐associated phenotypes [163]. In addition, Fn‐induced CXCL1 can recruit MDSCs via a defined signaling axis and is accompanied by reduced infiltration of antitumor CD8^+^ T cells, further amplifying immunosuppressive effects [132].

Comparable immunosuppressive patterns have been observed in other tumor types. In gastric cancer, increased abundance of *Methylobacterium* has been associated with shortened overall survival, characterized by exhaustion and functional impairment of CD8^+^ tissue‐resident memory T cells (T_RM cells) within the TME [164]. In OSCC, *P. gingivalis* can recruit MDSCs via the CXCL2‐IL‐6/IL‐8 axis and promote CD8^+^ T‐cell exhaustion [165].

#### 7.1.2 Immune Activation and Tumor Immunity Enhancement

With continued advances in this field, increasing evidence suggests that intratumoral bacteria not only affect tumor cells directly but can also promote the establishment and maintenance of antitumor immunity by directly or indirectly modulating the composition and functional states of immune parenchymal cells [1]. In specific contexts, selected intratumoral microbes may enhance effector‐cell recruitment, improve immune‐cell activation, or engage PRR‐associated signaling, thereby shifting the tumor immune microenvironment from a “low‐reactive” to a more “high‐reactive” state (Figure 5B).

For example, in PDAC, enrichment of taxa such as *Saccharopolyspora* and *Streptomyces* correlates with increased infiltration of granzyme B (GzmB)‐positive CD8^+^ T cells and prolonged survival [89]. In ESCC, *Streptococcus* spp. have been linked to chemokines such as CXCL9 and CXCL10, suggesting a role in promoting effector T‐cell recruitment and local immune surveillance [166]. Similarly, in melanoma, *Lachnoclostridium* has been associated with upregulation of comparable chemokine programs and enrichment of effector immune cells [167]. Collectively, these findings support the concept that certain intratumoral bacteria can enhance immune activity within tumors by shaping infiltration patterns and functional states of effector cells.

Notably, the immunological impact of intratumoral microorganisms is highly context dependent. *Clostridium butyricum* may enhance benefit from anti‐PD‐1 therapy by alleviating IL‐6‐mediated immunosuppression [168]. Conversely, Fn—typically regarded as pro‐tumorigenic—has been associated with improved immunotherapy outcomes in subsets of microsatellite‐stable (MSS) CRC cohorts under specific therapeutic contexts [169]. In addition, beneficial taxa such as Bifidobacterium can enhance antitumor immunity through activation of the STING pathway [119], and cohort‐based RNA‐seq analysis has further confirmed that intratumoral microbial features correlate with ICI response and TME traits, with intratumoral injection of selected strains synergizing with anti‐PD‐1 therapy in vivo [170].

Overall, these studies indicate that intratumoral bacteria can either attenuate or potentiate immune effector functions depending on tumor type, baseline immune context, and treatment setting; thus, both the direction and magnitude of immune modulation should be interpreted within a tumor‐ and therapy‐specific framework.

#### 7.1.3 Metabolic Modulation and Immune Crosstalk

Microbial metabolites are increasingly recognized as key mediators linking intratumoral bacteria to immune phenotypes, exerting clearly bidirectional effects on antitumor immunity [15]. To facilitate mechanistic integration across studies, Table 2 summarizes representative intratumoral bacterial metabolites/effectors, their host receptors/sensors/targets, and the reported impacts on TME and metastatic progression. On the one hand, specific metabolic axes can shape immunosuppressive ecosystems. For example, *Roseburia*‐derived butyrate promotes M2‐like macrophage polarization and lung metastasis by inhibiting HDAC2 and upregulating H19 [139]. In CRC, Fn‐derived succinate activates the SUCNR1‐HIF1α‐EZH2 axis, suppresses the cGAS‐IFN‐β pathway, and downregulates Th1‐type chemokines (CCL5/CXCL10), thereby limiting CD8^+^ T‐cell recruitment and attenuating responses to PD‐1 blockade [171]. In addition, lactate produced by *E. coli* can induce lactylation of RIG‐I in macrophages, inhibiting NF‐κB signaling and NLRP3 inflammasome activation while promoting regulatory T‐cell (Treg) differentiation [172]. In gastric cancer, *S. anginosus* converts arginine into ornithine via arginine deiminase (ADI), which has been linked to impaired CD8^+^ T‐cell function and immune evasion [161].

**TABLE 2:** Intratumoral bacterial metabolite/effector‐host target axes in the TME and metastasis.

Microbial metabolite/effector	Source	Host receptors/sensors/targets	Impact on TME	Impact on metastasis	References
SCFAs: acetate/propionate/butyrate	Anaerobic gut bacteria fermenting dietary fiber/indigestible carbohydrates	GPCRs (e.g., GPR109A); HDACs (class I/II, incl. HDAC3); ACSS2 (acetate‐utilization enzyme).	promote Treg and anti‐inflammatory tone; or enhance CD8+ activation via HDAC inhibition (e.g., ID2/CCL4 upregulation)	butyrate can enhance CD8+ cytotoxicity and suppress metastasis; low‐dose butyrate may drive M2 polarization; acetate may promote EMT/metastasis in some cancers (e.g., RCC/GBM)	[139, 176, 177, 178, 179, 180, 181]
Bile acids (e.g., primary: CA/TCA; secondary: DCA/LCA and their conjugates such as TDCA)	Microbiota‐mediated conversion of primary bile acids to secondary bile acids	TGR5; VEGFR2 (angiogenesis target); Wnt/β‐catenin (downstream node); CCR1 (CAF–CCL3 axis); CXCR6 (CXCL16–CXCR6 axis)	Downregulate CXCL16 to reduce NKT recruitment; induce CAF‐derived CCL3 to recruit MDSCs; overall immunosuppressive tendency (context specific)	Molecule and tumor specific: DCA promotes EMT/vasculogenic mimicry/metastasis; TDCA promotes CRC lymph‐node metastasis; LCA is bidirectional; TCA promotes CRC liver metastasis	[153, 176, 182, 183, 184]
Inosine	Inosine‐producing commensals (e.g., *Bifidobacterium pseudolongum*)	A2AR	Promotes Th1 differentiation; increases CD8+ infiltration and IFN‐gamma; serves as a metabolic fuel for effector T cells	Synergizes with PD‐L1 blockade to enhance immunity, indirectly restraining metastasis	[176, 185, 186]
IAA	Microbiota *Parabacteroides distasonis*; *Lactobacillus* spp. (pro‐tumor context)	AhR	*P. distasonis*‐derived IAA: enhances ferroptosis susceptibility and rewires metabolism; *Lactobacillus*‐derived IAA: can induce IL‐10 in TAMs and suppress immunity	*P. distasonis*‐derived IAA inhibits proliferation/metastasis; *Lactobacillus*‐derived IAA may promote pancreatic cancer progression	[173, 187, 188]
I3A	*Lactobacillus reuteri*	AhR (on CD8+ T cells)	Boosts CD8+ IFN‐gamma production; improves anti‐tumor immunity and ICI responsiveness	Predominantly indirect anti‐metastatic effects via immune activation	[173]
ILA	*Lactobacillus plantarum*	H3K27ac at the IL‐12a enhancer (epigenetic target)	Enhances DC IL‐12a production and activates CD8+ T cells	Indirectly suppresses metastasis through immune activation	[189]
Lactate	microbiota fermentation (gut; LAB such as *Lactobacillus/Bifidobacterium*) + tumor glycolysis.	HCAR1/GPR81; MCT1 (SLC16A1); HDACs; histone lactylation (Kla) machinery	Establishes an acidic, pro‐tumor niche and activates pseudohypoxia signaling	In lung adenocarcinoma, increased MCT1 is linked to enhanced metastatic potential	[190, 191, 192, 193, 194]
Ornithine	oral/intratumoral *Streptococcus* (e.g., *S. anginosus*)	Arginine availability/arginine metabolism axis (pathway‐level); downstream ornithine‐driven polyamine biosynthesis (e.g., ODC1 node)	Suppresses CD8+ T‐cell differentiation and infiltration and activates the MAPK pathway and induce chronic inflammation.	Promotes gastric cancer growth and metastasis	[161]
TMAO	Choline‐derived, microbiota‐dependent (microbe‐TMA‐TMAO axis; Clostridiales‐associated genera, e.g., *Blautia/Ruminococcus*)	PERK; caspase‐3; GSDME	Induces GSDME‐dependent pyroptosis and pro‐inflammatory cytokines; increases CD8+ infiltration and IFN‐gamma/TNF‐alpha; synergizes with anti‐PD‐1	Suppresses growth of metastatic lesions in TNBC primarily via immune enhancement	[195]
c‐di‐AMP	*Akkermansia muciniphila* (microbiota‐derived STING agonist)	STING	Activates the STING–type I IFN axis; promotes DC–NK crosstalk and anti‐tumor immunity	Indirect anti‐metastatic effects via immune activation	[196]
The virulence factor/surface protein of Fn	Fn (intratumoral enrichment; intracellular in tumor and immune cells)	Fap2–TIGIT; FadA–E‐cadherin–beta‐catenin; TLR4–MYD88–NF‐kappaB; Dps–PD‐L1 upregulation	Immunosuppressive ITME: inhibits NK/CD8+ function, recruits MDSCs, promotes T‐cell exhaustion, increases PD‐L1	Promotes EMT/invasion and a pre‐metastatic niche; associated with chemo/ICI resistance in some contexts	[6, 72, 92, 162]
Colibactin(genotoxin)	pks^+^ *Escherichia coli*(gut microbiota; can colonize tumor tissue/intratumoral niche)	Tumor‐cell DNA	Drives genomic instability and chronic damage stress, facilitating tumor initiation	Enhances malignant evolution and metastatic risk via mutation accumulation	[93, 94, 96, 197]
BFT	ETBF	E‐cadherin; β‐catenin; MAPK/NF‐κB; SMO (spermine oxidase); NOD1–NOTCH1.	Couples inflammation with immunosuppression, establishing a pro‐tumor inflammatory niche	Promotes barrier disruption, EMT, and invasion, facilitating metastasis	[98, 124, 198, 199]
CDT	*Campylobacter* spp.	DNA damage response (DSBs; ATM/ATR–CHK signaling); cell‐cycle checkpoint arrest	Promotes DNA damage‐associated remodeling and inflammatory signaling	Increases progression/metastatic risk via genomic instability and malignant traits	[99]
Gingipains	*P. gingivalis*	TLR signaling; MAPK/ERK; JAK/STAT; CXCL2–IL‐6/IL‐8 inflammatory axis	Recruits MDSCs and drives CD8+ exhaustion; reinforces inflammatory‐immunosuppressive loops	Enhances invasion and metastatic potential via multi‐pathway cooperation	[117, 200, 201, 202]
CagA (and related virulence factors)	*H. pylori*	Wnt/β‐catenin (CagA‐linked); inflammation‐associated programs; epigenetic remodeling programs	Sustains chronic inflammation and immune imbalance; amplifies oncogenic signaling	Primarily indirect effects on metastasis through malignant progression	[135, 203, 204]
LPS; peptidoglycan	Multiple bacterial taxa	PRRs including TLRs and NOD‐like receptors (NLR/NOD family).	Triggers NF‐kappaB‐centered inflammation that can evolve toward immunosuppression (M2/MDSC/Treg increase)	Facilitates metastasis via coupled inflammation‐immunosuppression, angiogenesis, and EMT	[205, 206]

Abbreviations: A2AR, adenosine A2A receptor; ACSS2, acyl‐CoA synthetase short‐chain family member 2; ADI, arginine deiminase; AhR, aryl hydrocarbon receptor; ATM, ataxia telangiectasia mutated; ATR, ATM and Rad3‐related; BFT, *B. fragilis* toxin (fragilysin); CA, cholic acid; CAF, cancer‐associated fibroblast; CCL4, C–C motif chemokine ligand 4; CCR1, C–C chemokine receptor 1; c‐di‐AMP, cyclic di‐AMP; CDT, cytolethal distending toxin; CHK, checkpoint kinase; CXCL16, C–X–C motif chemokine ligand 16; CXCR6, C–X–C chemokine receptor 6; DC, dendritic cell; DCA, deoxycholic acid; DSBs, DNA double‐strand breaks; EMT, epithelial–mesenchymal transition; ERK, extracellular signal‐regulated kinase; ETBF, enterotoxigenic *Bacteroides fragilis*; FadA, *F. nucleatum* adhesin FadA; Fap2, *Fusobacterium nucleatum* adhesin Fap2; GBM, glioblastoma; GPCRs, G protein–coupled receptors; GPR109A, G protein–coupled receptor 109A; GSDME, gasdermin E; H3K27ac, histone H3 lysine 27 acetylation; HCAR1, hydroxycarboxylic acid receptor 1 (GPR81); HDAC3, histone deacetylase 3; HDACs, histone deacetylases; I3A, indole‐3‐aldehyde; IAA, indole‐3‐acetic acid; ID2, inhibitor of DNA binding 2; IFN, interferon; IFN‐I, type I interferon; IL‐12a, interleukin 12 subunit alpha; ILA, indole‐3‐lactic acid; ITME, intratumoral microenvironment; JAK/STAT, Janus kinase/signal transducer and activator of transcription; Kla, histone lysine lactylation; LCA, lithocholic acid; LPS, lipopolysaccharide; MAPK, mitogen‐activated protein kinase; MCT1, monocarboxylate transporter 1 (SLC16A1); MDSC(s), myeloid‐derived suppressor cell(s); MYD88, myeloid differentiation primary response 88; NF‐κB, nuclear factor kappa B; NK, natural killer cell; NKT, natural killer T cell; NLR, NOD‐like receptor; LAB, lactic acid bacteria; NOD1, nucleotide‐binding oligomerization domain‐containing protein 1; NOTCH1, Notch homolog 1; ODC1, ornithine decarboxylase 1; PD‐L1, programmed death‐ligand 1; PERK, protein kinase R‐like endoplasmic reticulum kinase (EIF2AK3); PRRs, pattern‐recognition receptors; RCC, renal cell carcinoma; SCFAs, short‐chain fatty acids; SLC16A1, solute carrier family 16 member 1; SMO, spermine oxidase; STING, stimulator of interferon genes (TMEM173); TAM(s), tumor‐associated macrophage(s); TCA, taurocholic acid; TDCA, taurodeoxycholic acid; TGR5, G protein–coupled bile acid receptor 1 (GPBAR1); TIGIT, T‐cell immunoreceptor with Ig and ITIM domains; TLR4, Toll‐like receptor 4; TLRs, Toll‐like receptors; TMA, trimethylamine; TMAO, trimethylamine N‐oxide; TME, tumor microenvironment; Treg, regulatory T cell; VEGFR2, vascular endothelial growth factor receptor 2.

Conversely, in specific tumor types and therapeutic contexts, certain microbial metabolites can enhance effector immunity and improve treatment responsiveness. For example, *Lactobacillus reuteri* catabolizes tryptophan into indole‐3‐aldehyde (I3A), which acts as an aryl hydrocarbon receptor (AhR) agonist in CD8^+^ T cells to enhance IFN‐γ production and increase sensitivity to immune checkpoint inhibitors (ICIs) [173]. *Akkermansia muciniphila* also exhibits immune‐activating potential: its EVs can reprogram macrophages toward an M1‐like phenotype and activate CD8^+^ T cells [174]; moreover, its outer membrane protein Amuc_1434 can reduce PD‐L1 levels on CRC cells, thereby enhancing cytotoxic immune responses. In addition, studies suggest that under certain conditions, *A. muciniphila* may enter the circulation and be detected within tumor tissues; one proposed mechanism is that it reshapes tumor metabolism via a “microbiota–metabolism axis,” suppressing key pathways such as glycolysis, glutamine metabolism, and nucleotide biosynthesis, thereby restricting energy supply and biosynthetic capacity to inhibit tumor cell proliferation [175].

### 7.2 Nonimmune Parenchymal Components: Tumor Microenvironment Modulated by Intratumoral Bacteria

Although the preceding sections primarily focus on immune‐mediated mechanisms, increasing evidence indicates that the effects of intratumoral bacteria on tumor progression extend well beyond immune regulation and involve direct interactions with nonimmune components of the TME. In situ analyses and imaging‐based studies further suggest that intratumoral bacteria are not uniformly distributed. Rather, bacterial signals are frequently detected intracellularly within tumor and immune cells [1, 51], while additional enrichment has been observed in stromal regions [207] and hypoxic microdomains [208]. This nonrandom spatial organization provides a structural basis for bacteria‐stroma interactions and supports the concept that nonimmune TME elements—particularly cancer‐associated fibroblasts (CAFs), the ECM, and vascular structures—serve as critical substrates through which bacteria remodel the stromal niche and promote malignant progression.

Among these nonimmune targets, CAFs appear to represent a central node of bacteria‐driven stromal reprogramming. In CRC, Fn preferentially localizes within stromal regions and directly interacts with CAFs, driving their conversion toward inflammatory and tumor‐promoting phenotypes characterized by increased secretion of IL‐6, CXCL1, and IL‐8 [207]. This shift in CAF state can enhance tumor cell proliferation and local expansion even in the absence of direct immune‐cell involvement. Mechanistically, Fn activates a TLR2‐YAP‐CTGF signaling axis in CAFs, thereby promoting ECM remodeling and growth‐supportive stromal functions [209]. Similar stromal effects have been reported in gastric cancer, where *H. pylori* alters fibroblast and CAF behavior. Exposed fibroblasts show increased Serpin E1 secretion and activation of the p38 MAPK–VEGFA pathway, contributing to angiogenesis and tumor growth [210]. In addition, *H. pylori*‐derived EVs carrying microRNAs such as miR‐124‐3p can enhance fibroblast migration, further supporting bidirectional communication between bacteria and stromal cells [211].

Intratumoral bacteria also influence tumor progression by remodeling the ECM and its associated receptor networks. In CRC, *Peptostreptococcus anaerobius* promotes carcinogenesis through its surface protein PCWBR2, which binds integrin α2/β1 on tumor cells and activates the PI3K‐Akt‐NF‐κB signaling cascade [212]. In OSCC, *P. gingivalis* has been shown to induce inflammatory mediators and MMPs, thereby disrupting ECM homeostasis and creating a permissive microenvironment for tumor expansion and invasion [202]. Beyond induction of host MMP programs, bacterial enzymes themselves may directly participate in matrix remodeling: *P. gingivalis* secretes gingipains that can cleave key ECM components such as fibronectin and tenascin‐C [213]. These findings indicate that intratumoral bacteria can alter both the biochemical and mechanical properties of the stromal scaffold, thereby facilitating invasion and dissemination.

Collectively, these observations indicate that the contribution of intratumoral bacteria to tumor progression is not limited to immune crosstalk. By reshaping CAF phenotypes, modulating ECM‐integrin signaling, promoting matrix degradation, and activating vascular‐associated pathways, intratumoral bacteria can remodel the non‐immune stromal niche in ways that support tumor growth, local invasion, and metastatic progression.

## 8 Diagnostic and Prognostic Value of Intratumoral Bacteria in Tumor Initiation and Progression

Recent studies indicate that intratumoral bacteria not only participate in tumor initiation, progression, and metastasis, but also indicate potential clinical utility in cancer screening, risk stratification, and prognostic evaluation (Table 3). By systematically comparing multiple solid tumors with matched normal tissues, Nejman et al. first revealed that different tumor types harbor relatively tumor‐specific intratumoral microbial lineage signatures, establishing the presence of stable and distinctive microbial ecosystems within the TME [1]. This discovery laid an important foundation for tumor classification and biomarker discovery based on intratumoral microbial features.

**TABLE 3:** Intratumor bacteria as diagnostic and prognostic markers in cancer patients.

Diagnosis/prognosis marker	Intratumor bacterial	Tumor type	Clinical role	Method	Human cohort sample	Performance metrics	Ref.
Diagnosis	*Fusobacterium, Peptostreptococcus, Campylobacter*	CRC	Distinguish between normal and tumor tissue	NGS, 16S rRNA genes sequencing	29 CRC patients (Japan)	NA	[19]
Diagnosis, prognosis	*Micrococcus, Microbacterium* Streptococcaceae family microbes	HPV‐independent endocervical adenocarcinoma	Distinguish between normal and tumor tissue and different tumor subtypes, shortened OS, and RFS	16S ribosomal DNA sequencing	45 HPVI ECA patients	Tumor vs. adjacent non‐tumor: 6‐genus model, training AUC = 0.764, validation AUC = 0.694; subtype (GEA vs CCC): 6‐genus model, AUC = 0.944	[214]
Diagnosis, prognosis	*Pseudomonas, Rhodococcus* *Faecalibacterium*, *Bacteroides*	Pancreatic cancer	Differentiating PDAC from nonmalignant lesions, reflecting progress	NGS	Feces: 193 (156 pancreatic tumor + 37 healthy); tissue: 362 fresh pancreatic tissues (tumor + paired NAT)	NA	[215]
Diagnosis	*Oral pathogens, Porphyromonas gingivalis, Aggregatibacter actinomycetemcomitans*, Phylum Fusobacteria and its genus Leptotrichia	Pancreatic cancer	Predicting breast cancer risk	16S rRNA gene sequencing	Cases 361; controls 371	Risk association (not AUC): *P. gingivalis* OR = 1.60; *A. actinomycetemcomitans* OR = 2.20; Fusobacteria phylum OR = 0.94; *Leptotrichia* OR = 0.87 (all *p* < 0.05)	[216]
Prognosis	*OCS1*: (*Fusobacterium/oral pathogens*) *OCS2*: (*Firmicutes/Bacteroidetes, saccharolytic*) *OCS3*: (*Escherichia/Pseudescherichia/Shigella*)	CRC	Stratify subgroups with significant prognostic differences	16S rRNA gene sequencing	Cases 361; controls 371	NA	[18]
Prognosis	*Proteobacteria, Actinobacteria*	HCC	Predicting prognosis after surgery	FISH, 16S rRNA gene sequencing	91 HCC patients after hepatectomy	Tumor vs. paratumor classification accuracy 73.2%; hepatotype B vs. A: OS HR = 0.296 (*p* = 0.005), RFS HR = 0.504 (*p* = 0.025)	[217]
Prognosis	*Microbiome*	Breast, lung cancer or CRC	Reduced microbiome richness is significantly associated with shorter survival	16S rRNA gene sequencing	79 metastatic cases (LN 16, lung 30, liver 33)	NA	[218]
Prognosis	*Corynebacterium, Staphylococcus*	Nasopharyngeal carcinoma	OS (risk ratio, disease‐free survival, and survival without distant metastases) was significantly lower in patients with high bacterial loads	16S rRNA sequencing, quantitative polymerase chain reaction	802 NPC (training cohort; two hospitals)	High vs. low risk: DFS HR = 2.90; DMFS HR = 3.18; OS HR = 3.41; *p* < 0.001	[219]
Prognosis	*Bacteroides, Alloprevotella, Parvimonas, and Dialister*	Nasopharyngeal carcinoma	The four‐bacterial signature serves as an independent prognostic indicator for nasopharyngeal carcinoma and can effectively stratify patients by prognostic risk; the abundance of risk bacteria in the signature is negatively correlated with patients' disease‐free survival, distant metastasis‐free survival and overall survival.	16S rRNA sequencing, RNA‐seq	491 NPC + 36 normal tissues	High‐risk vs. low‐risk: DFS HR = 2.80 (95% CI 1.51–5.18; DMFS HR = 4.00 (95% CI 1.77–9.01); OS HR = 3.45 (95% CI 1.77–6.72); *p* < 0.001	[220]
Prognosis	*Pseudomonadaceae*	PLCs	Increased relative abundance of bacteria positively correlates with better prognosis	MiSeq sequencing	28 PLC FFPE tissue samples	NA	[221]
Prognosis	*Pseudoxanthomonas‐Streptomyces‐Saccharopolyspora‐Bacillus clausii*	PDAC	Specific microbiomes are associated with better prognosis (long‐term survival)	IHC, 16S rRNA sequencing	28 PLC FFPE tissue samples	Prognostic model AUC = 97.51–99.17 for predicting long‐term survival	[89]

Abbreviations: AUC, area under the receiver operating characteristic curve; CCC, clear cell carcinoma; CPS II, Cancer Prevention Study II; CRC, colorectal cancer; CSBJ, Computational and Structural Biotechnology Journal; DFS, disease‐free survival; DMFS, distant metastasis‐free survival; EAD, esophageal adenocarcinoma; EBV, Epstein–Barr virus; ECA, endocervical adenocarcinoma; ESCC, esophageal squamous cell carcinoma; FFPE, formalin‐fixed paraffin‐embedded; FISH, fluorescence in situ hybridization; GEA, gastric‐type endocervical adenocarcinoma; HCC, hepatocellular carcinoma; HPV, human papillomavirus; HPVI ECA, human papillomavirus‐independent endocervical adenocarcinoma; HR, hazard ratio; IHC, immunohistochemistry; LN, lymph node; MiSeq, Illumina MiSeq sequencing platform; NA, not available/not reported; NAT, adjacent normal tissue; NGS, next‐generation sequencing; NPC, nasopharyngeal carcinoma; OR, odds ratio; OS, overall survival; PDAC, pancreatic ductal adenocarcinoma; PFS, progression‐free survival; PLC, primary liver cancer; PLCO, Prostate, Lung, Colorectal and Ovarian Cancer Screening Trial; qPCR, quantitative polymerase chain reaction; RFS, relapse‐free survival; RNA‐seq, RNA sequencing; SHIVA, SHIVA clinical trial; TCGA, The Cancer Genome Atlas; TNM, Tumor‐Node‐Metastasis staging; WES, whole‐exome sequencing; WGS, whole‐genome sequencing.

### 8.1 Diagnostic Value

Across multiple tumor types, intratumoral bacteria have been shown to correlate closely with the presence of tumor tissues and key biological features, with CRC representing one of the most extensively studied models. Early studies reported that Fn is significantly enriched in CRC tissues, whereas *Bacteroides* species predominate in normal mucosa, providing initial support for distinguishing tumor from normal tissue based on microbial characteristics [19]. Building on this, multiple human cohort studies based on tumor tissues further demonstrated that intratumoral Fn abundance is closely associated with increased tumor invasiveness, more advanced stage, and higher metastatic risk in CRC [14, 222, 223].

However, from a clinical diagnostic perspective, the current evidence primarily supports Fn as a biomarker related to tumor biological behavior and risk stratification, rather than as a validated staging‐discriminative diagnostic indicator. Specifically, although many studies report higher Fn abundance in stage III‐IV CRC or metastatic CRC, these differences between stage I‐II and stage III‐IV are largely reflected as statistical associations, while quantitative performance metrics required for clinical staging discrimination—such as sensitivity, specificity, positive/negative predictive values, or ROC curves—are generally lacking [14, 222, 223]. Therefore, intratumoral Fn cannot yet be considered a reliable stand‐alone diagnostic tool to distinguish early‐stage from advanced‐stage CRC.

Regarding comparisons with conventional serum tumor markers, no prospective studies have yet performed head‐to‐head performance evaluations of intratumoral Fn versus carcinoembryonic antigen (CEA) or carbohydrate antigen 19‐9 (CA19‐9) within the same patient cohort under a unified analytical model. Existing evidence suggests that CEA and CA19‐9 mainly reflect systemic tumor burden and late‐stage disease, whereas intratumoral Fn more likely captures local ecological niche remodeling, immune microenvironment alterations, and metastatic potential [14, 222]. Given this distinction, a more appropriate positioning at present is to regard intratumoral Fn as a complementary biomarker that can be integrated with conventional clinical, pathological, and molecular indicators within multiparameter models, rather than as a single marker that can replace CEA or CA19‐9 for diagnosis or staging.

Beyond CRC, diagnostic associations of intratumoral bacteria have also been reported in other cancers. For example, in pancreatic cancer, enrichment of oral‐associated bacteria such as *Aggregatibacter actinomycetemcomitans* and *P. gingivalis* in tumor tissues has been linked to increased pancreatic cancer risk, whereas certain *Clostridium* species may be associated with reduced risk [216]. In addition, intratumoral bacterial features may also indicate metastatic risk. For instance, Fn levels are higher in CRC liver metastases than in the corresponding primary lesions [74], and tumor‐associated microbial signals detected in peripheral blood of patients with advanced CRC are closely related to metastatic risk and overall survival [224, 225]. Collectively, these studies support the potential value of intratumoral bacteria in cancer diagnosis and risk assessment, although clinical translation still depends on standardized detection methods and systematic evaluation.

### 8.2 Prognostic Value

Intratumoral microorganisms also show substantial clinical relevance for prognostic assessment. In a study including 423 CRC patients, Mouradov et al. stratified tumors into three molecular subtypes (OCS1‐3) based on carcinogenesis‐related microbial community features, and these subtypes exhibited significant survival differences, providing new perspectives for patient stratification and individualized therapy [18]. In HCC, researchers proposed a “hepatotype” classification system using microbial clustering analysis and demonstrated that it could independently predict postoperative prognosis [217]. Similarly, three microbial subtypes (MS1‐3) identified in gastric cancer were closely associated with differential responses to immunotherapy and long‐term survival, suggesting that intratumoral microbial ecology may participate in regulating treatment sensitivity [226].

At the level of specific taxa, multiple tumor‐tissue‐based studies consistently report that high Fn abundance is significantly associated with tumor progression, metastatic propensity, and unfavorable overall survival in CRC, pancreatic cancer, ESCC, and cervical cancer [88, 132, 227, 228]. Notably, studies published in Gut and Clinical Cancer Research showed that even after adjusting for TNM stage and other clinicopathological factors, intratumoral Fn remains an independent adverse prognostic factor, further supporting its potential value in risk stratification [14, 223]. In contrast, in primary liver cancer, increased relative abundance of *Pseudomonas* has been associated with better survival outcomes, indicating that the prognostic significance of intratumoral bacteria is strongly context dependent across tumor types [221].

In addition, intratumoral microbial diversity may influence patient outcomes. In PDAC, long‐term survivors (> 5 years) exhibit significantly higher intratumoral microbial α‐diversity than short‐term survivors, and some potentially protective bacteria (e.g., *C. butyricum*) may improve prognosis through metabolic regulation [89]. However, diversity metrics are highly sensitive to sequencing depth, sample processing, and analytical pipelines, and their prognostic implications are not consistent across tumor types. Thus, at present, they are better suited as auxiliary indicators for stratification and mechanistic studies rather than as independent clinical predictive tools.

Overall, existing evidence suggests that the value of intratumoral bacteria in cancer diagnosis and prognostic evaluation is better suited to risk stratification and integrated model construction, rather than replacing established clinical indicators. Future studies should, under unified frameworks for tissue‐based detection and quality control, combine large‐scale prospective human cohorts with multicenter validation to systematically assess the true clinical benefit of intratumoral microbial features across different tumor types and clinical scenarios.

## 9 The Efficacy of Intratumoral Microbiota in Cancer Treatment

The bacteria within the TME not only influence tumor initiation and progression but also, as accumulating evidence suggests, play a critical role in shaping cancer treatment outcomes. The efficacy of various therapeutic modalities—ranging from chemotherapy and radiotherapy to immunotherapy—can be modulated by the intratumoral bacteria. These microorganisms may either enhance or attenuate treatment responses through mechanisms such as drug metabolism, immune modulation, or alteration of tumor cell sensitivity.

### 9.1 Effects of Intratumoral Microbiota on Chemotherapy Efficacy

An expanding body of evidence indicates that intratumoral microorganisms represent critical determinants of chemotherapy efficacy. Their impact can manifest as promotion of chemoresistance or, in specific contexts, enhancement of therapeutic responsiveness. Distinct from tumor cell‐intrinsic genetic or epigenetic mechanisms, intratumoral microbes can influence treatment outcomes by participating in drug metabolism, reshaping DNA damage responses and cellular stress‐adaptation programs, and modulating chemotherapy‐associated immune effects, thereby providing an additional framework to explain inter‐patient variability in chemotherapy responses [229, 230, 231].

Among resistance mechanisms, microbe‐mediated inactivation of chemotherapeutic agents was one of the earliest clearly established paradigms. Geller et al. reported that *γ‐proteobacteria* enriched in PDAC can express cytidine deaminase, converting gemcitabine into inactive metabolites and thereby markedly attenuating its antitumor activity. In human tumor specimens, widespread intratumoral bacterial signals have been associated with gemcitabine resistance [232]. Similar concepts have been extended to CRC, where intratumoral *Escherichia coli* and oral‐associated pathogens, including *Aggregatibacter actinomycetemcomitans* and *P. gingivalis*, have been implicated in local drug metabolism and enzyme regulation, potentially exacerbating chemoresistance [233, 234, 235].

Beyond direct drug metabolism, intratumoral microbes can systemically blunt chemotherapy‐induced tumor cell death by activating cytoprotective pathways. In CRC, Fn induces autophagy by activating the TLR4‐MYD88 axis and downregulating miR‐18a and miR‐4802, thereby increasing tolerance to 5‐fluorouracil (5‐FU) and oxaliplatin. Additional studies further suggest that Fn can dampen cytotoxic responses to multiple chemotherapeutics by suppressing ferroptosis and blocking pyroptotic pathways [91, 147, 236, 237]. Importantly, intratumoral microbiota do not uniformly impair chemotherapy efficacy; in certain settings, specific microbes can enhance chemotherapy responses through immunomodulatory mechanisms. Daillère et al. showed that *Enterococcus hirae* [238] and *Barnesiella intestinihominis* [239] can promote infiltration of CD8^+^ T cells and γδ T cells and strengthen IFN‐γ‐dependent antitumor immunity, thereby significantly improving cyclophosphamide efficacy. In germ‐free or antibiotic‐treated models, these immune effects were markedly attenuated, resulting in reduced chemotherapy responsiveness and functional chemoresistance.

Overall, intratumoral microorganisms shape chemotherapy outcomes through multiple, non‐mutually exclusive mechanisms, including metabolic drug inactivation, modulation of DNA damage and stress‐response pathways, reprogramming of cell death programs, and regulation of chemotherapy‐associated antitumor immunity. Given the substantial heterogeneity in both tumor types and their intratumoral microbial communities, future efforts to precisely identify resistance‐associated versus sensitization‐associated taxa—and to implement tailored microbiome interventions—may offer new opportunities to overcome chemoresistance and optimize individualized treatment strategies.

### 9.2 Effects of Intratumoral Microbiota on Radiotherapy Efficacy

Radiotherapy is a cornerstone modality for many solid tumors, and its efficacy depends not only on the intrinsic radiosensitivity of tumor cells but also on the TME [240]. Emerging evidence suggests that tumor‐associated microorganisms may promote radioresistance and increase post‐treatment relapse risk by reshaping tumor metabolic states, modulating radiation‐related stress and damage‐response processes, and engaging inflammatory and immune networks. For example, tumor‐resident microbes can enhance chemoradiation resistance via metabolic rewiring [190], and persistence of specific bacteria following neoadjuvant chemoradiotherapy has been associated with increased recurrence risk [241]. Although studies directly interrogating intratumoral microbiota in radiotherapy remain relatively limited, convergent clinical associations and mechanistic clues support a reproducible link between tumor‐associated microbes and radiotherapy outcomes, warranting more systematic causal validation and mechanistic dissection.

At the mechanistic level, microbe‐driven metabolic reprogramming has been proposed to compromise radiotherapy efficacy. One study reported that oral vancomycin–associated microbial perturbations increased systemic and intratumoral butyrate levels; as a HDAC inhibitor, butyrate may alter chromatin states and DNA damage repair processes, thereby reducing tumor cell sensitivity to ionizing radiation and weakening antitumor effects [242]. In cervical cancer, intratumoral *Lactobacillus iners*, a potent producer of L‐lactate, may induce radio‐ and chemoradiation resistance through lactate‐driven metabolic rewiring and associated signaling alterations [190], supporting a potential “microbiota‐metabolism‐radiotherapy response” axis in specific tumor contexts.

From a clinical perspective, associations between tumor microbial features and responses to chemoradiation have been reported in selected cohorts. Metagenomic profiling of locally advanced rectal cancer tissues showed that multiple core taxa (including *Streptococcus equinus* and *Blautia producta*) were significantly associated with resistance to neoadjuvant chemoradiotherapy, suggesting that tumor‐associated microbial signatures may serve as candidate biomarkers for treatment stratification [243]. In addition, a colon cancer study found that increased abundance of a specific operational taxonomic unit (OTU_104) correlated with tumor recurrence, implying that residual tumor‐associated microbes after treatment may contribute to maintenance of a relapse‐permissive microenvironment [244]. Notably, the directionality of microbe‐radiotherapy associations may vary by tumor type. For example, in OSCC, Fn positivity has been associated with lower recurrence and longer metastasis‐free survival in one study [245], underscoring strong tumor‐context dependence.

Beyond direct modulation of radiosensitivity, microorganisms may indirectly influence radiotherapy outcomes by shaping radiation‐associated immune effects. Evidence suggests that antibiotic interventions or altered microbial composition can modify radiotherapy‐induced antitumor immunity. For instance, vancomycin has been reported to enhance radiotherapy‐driven antitumor immune responses and suppress tumor growth, whereas dysbiosis induced by broad‐spectrum antibiotics may impair radiotherapy efficacy [245, 246]. Moreover, microbiota‐derived tryptophan metabolites (e.g., I3A and kynurenic acid) have been shown to confer long‐term radioprotection in vivo, further highlighting the complex, bidirectional regulatory potential of microbial metabolites in radiotherapy responses [247].

Overall, current evidence supports the concept that tumor‐associated microorganisms may influence radiotherapy outcomes through multiple pathways, including metabolic rewiring, modulation of DNA damage responses and immune effector programs, and maintenance of recurrence‐associated microenvironments. Although this area remains at an early stage, continued mechanistic elucidation of intratumoral and tumor‐associated microbial functions may advance both predictive biomarker development for radiotherapy response and the identification of actionable targets to sensitize tumors or mitigate resistance.

### 9.3 Effects of Intratumoral Microbiota on the Efficacy of Cancer Immunotherapy

With the rapid clinical expansion of ICIs and adoptive cell therapies, inter‐patient heterogeneity in therapeutic response has become a central challenge in oncology practice. Beyond tumor mutational burden and host immune status, an increasing number of studies indicate that the composition and functional properties of the intratumoral microbiome are important determinants of immunotherapy efficacy and resistance, and that these effects are strongly context dependent across tumor types [248].

Early work primarily highlighted microbiome‐level regulation of immunotherapy responsiveness. Vétizou et al. demonstrated that the presence of *B. fragilis* is closely linked to the efficacy of CTLA‐4 blockade; oral supplementation with *B. fragilis* or fecal microbiota transplantation (FMT) from responders markedly restored responsiveness to anti‐CTLA‐4 therapy in germ‐free or nonresponder mice [249]. Similarly, Sivan et al. showed that transferring the microbiota of PD‐L1‐responsive JAX mice into TAC mice substantially enhanced anti‐PD‐L1 efficacy against melanoma, and further identified *Bifidobacterium* as a key functional taxon [250]. Subsequent studies across multiple tumor models have confirmed that microbiome interventions can broadly augment immunotherapy efficacy, providing experimental support for FMT‐ or probiotic‐based adjuvant strategies [186, 251, 252, 253, 254].

As mechanistic understanding has deepened, intratumoral microbes have been shown to directly shape immunotherapy‐relevant immunosuppressive or immune‐activated states. Multiple studies report that bacteria‐enriched tumor regions are frequently accompanied by elevated expression of immune checkpoint molecules such as PD‐1, PD‐L1, and CTLA‐4, suggesting a potential role for intratumoral bacteria in immune evasion [255]. For example, in CRC, *Clostridium* is significantly enriched in patients who are insensitive to ICIs [255]. In ESCC, Fn enhances PD‐L1 transcription via its Dps protein and suppresses T‐cell function, thereby attenuating responses to anti‐PD‐1 therapy [256]. In addition, tumor metabolism‐associated microbial signatures correlate closely with T‐cell exclusion phenotypes and immunotherapy responsiveness, further supporting a critical role for the intratumoral microbiome–metabolism–immunity axis in determining ICI efficacy [257]. Moreover, a cohort‐based RNA‐seq analysis further linked intratumoral microbial features to ICI response and TME traits, and intratumoral delivery of selected taxa synergized with anti–PD‐1 therapy in vivo.

Conversely, specific beneficial microbes and their metabolites can potentiate immunotherapy responses. In melanoma models, *L. reuteri* produces I3A, activates the AhR, enhances IFN‐γ production by CD8^+^ T cells, and markedly improves ICI efficacy [173]. Likewise, intratumoral *Bifidobacterium* activates the STING pathway, converting immunotherapy‐refractory tumors into responders and significantly enhancing anti‐CD47 efficacy in CRC and lymphoma models [119]. Moreover, enrichment of *Streptococcus* in ESCC is associated with increased CD8^+^ T‐cell infiltration and concordant improvement in anti‐PD‐1 treatment outcomes [166].

From a clinical association perspective, intratumoral microbial features are closely linked to immunotherapy outcomes. In metastatic non‐small cell lung cancer (NSCLC), higher intratumoral *Fusobacterium* abundance has been associated with significantly worse overall survival and progression‐free survival following ICI therapy [32]. In gastric cancer, Epstein–Barr virus (EBV)‐positive tumors exhibit increased immune infiltration and higher expression of immune checkpoint‐related genes, suggesting that intratumoral microbial contexts may contribute to determining responses to anti‐PD‐L1 antibodies (e.g., avelumab) [258]. Notably, the directionality of microbiome effects is not uniform across tumor types, further underscoring the strong context dependence of intratumoral microbial influences on immunotherapy [259, 260].

Despite growing evidence across multiple levels, key questions remain unresolved, including tissue‐specific effects of microbial metabolites, the dual roles of SCFAs such as butyrate, the potential contributions of non‐bacterial microorganisms in immunotherapy, and the long‐term impact of antibiotic exposure prior to treatment. Addressing these issues will be essential for clarifying causal pathways and identifying actionable therapeutic targets. Notably, recent work also suggests that targeting the PD‐L2‐RGMb interaction can overcome microbiome‐related resistance to PD‐1 pathway inhibitors, offering a new conceptual strategy for intercepting microbiota‐driven immune tolerance [252, 261, 262].

Overall, current evidence supports that intratumoral microbiota plays a pivotal role in immunotherapy efficacy by regulating immune checkpoint expression, shaping immune‐cell infiltration patterns, remodeling metabolism‐immunity axes, and determining states of sensitivity versus resistance. As our understanding of intratumoral microbial functions and spatial organization continues to deepen, direct targeting of intratumoral microbes and their immunoregulatory networks may represent a promising direction for optimizing immunotherapy and overcoming resistance.

## 10 Applications of Intratumoral Microbiota in Cancer Therapy

**FIGURE 6:**
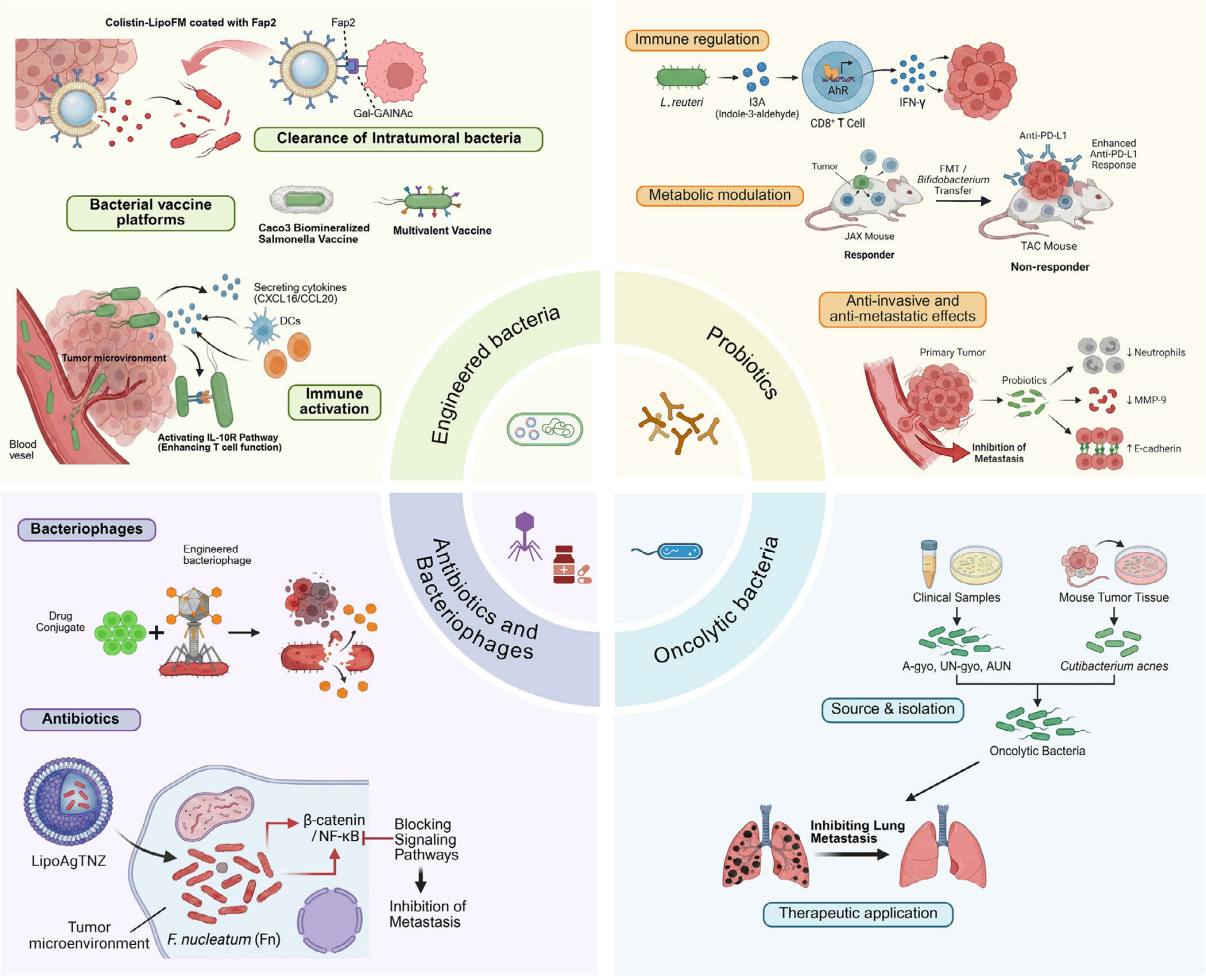
Emerging therapeutic strategies targeting the intratumoral microbiota for cancer treatment. Current microbiome‐targeted interventions can be broadly categorized into four distinct modalities. (1) Engineered bacteria and vaccines: Advanced delivery systems, such as Fap2‐coated liposomes (Colistin‐LipoFM), specifically recognize tumor‐expressed Gal‐GalNAc to precisely eradicate intratumoral bacteria. Concurrently, biomineralized bacterial vaccines (e.g., CaCO_3_‐mineralized Salmonella) and multivalent vaccines promote the recruitment and activation of APCs and DCs, which secrete chemokines (CXCL16/CCL20) and enhance T‐cell anti‐tumor functions via the IL‐10R pathway. (2) Probiotics and microbiota modulation: Specific probiotics exert profound immunomodulatory and anti‐metastatic effects. For instance, *Lactobacillus reuteri* secretes I3A to activate the AhR pathway in CD8+ T cells, stimulating IFN‐γ production. Furthermore, FMT or the transfer of specific commensals (e.g., *Bifidobacterium*) from immunotherapy‐responsive hosts to non‐responders significantly enhances anti‐PD‐L1 efficacy. Probiotics also actively impede metastasis by reducing neutrophil infiltration, downregulating MMP‐9, and restoring E‐cadherin expression. (3) Antibiotics and bacteriophages: Engineered bacteriophage‐drug conjugates enable targeted ablation of both specific intratumoral bacteria and adjacent cancer cells. Additionally, specialized nanocarriers are deployed into the TME to selectively clear pro‐tumoral pathogens like Fn. This targeted clearance disrupts essential oncogenic cascades, such as the β‐catenin and NF‐κB signaling pathways, thereby inhibiting tumor metastasis. (4) Oncolytic bacteria: Specific bacterial strains isolated from clinical samples or murine tumor tissues (e.g., *Cutibacterium acnes*) exhibit intrinsic oncolytic properties. These bacteria can selectively colonize tumor tissues, directly driving tumor cell destruction and effectively suppressing distant dissemination, such as lung metastasis. AhR, aryl hydrocarbon receptor; APCs, antigen‐presenting cells; CaCO_3_, calcium carbonate; CCL20, C‐C motif chemokine ligand 20; CXCL16, C‐X‐C motif chemokine ligand 16; DCs, dendritic cells; E‐cadherin, epithelial cadherin; Fap2, *Fusobacterium* autotransporter protein 2; FMT, fecal microbiota transplantation; Fn, *Fusobacterium nucleatum*; Gal‐GalNAc, galactose‐N‐acetylgalactosamine; I3A, indole‐3‐aldehyde; IFN‐γ, interferon‐gamma; IL‐10R, interleukin‐10 receptor; LipoAgTNZ, liposomal AgTNZ formulation; LipoFM, Fap2‐coated liposome formulation; MMP‐9, matrix metalloproteinase 9; NF‐κB, nuclear factor kappa‐B; anti‐PD‐L1, anti‐programmed death‐ligand 1; TME, tumor microenvironment; β‐catenin, beta‐catenin.

In recent years, therapeutic strategies centered on the intratumoral microbiota have emerged as a major frontier in oncology research. Although this field remains predominantly preclinical and rigorous randomized controlled clinical evidence is still limited, a broad range of animal studies and early translational investigations have consistently suggested that targeting or harnessing tumor‐resident microorganisms may offer unique advantages in enhancing antitumor efficacy, improving immunotherapy responsiveness, and overcoming treatment tolerance/resistance [222]. With advances in nanotechnology, synthetic biology, and materials science, natural bacterial strains can now be genetically engineered or structurally/chemically functionalized to enable tumor‐targeted delivery, remodeling of the tumor immune microenvironment, and vaccine‐like applications [14]. Moreover, certain naturally occurring intratumoral bacteria intrinsically display direct oncolytic activity and immune‐stimulatory capacity, providing valuable resources for developing novel biotherapeutic modalities [223]. In parallel, interventions such as antibiotics, bacteriophages, and probiotics can reshape intratumoral ecological states and thereby influence tumor growth, therapeutic response, and metastasis‐related processes. These emerging therapeutic strategies can be conceptually categorized into four distinct modalities (Figure 6). Based on these treatment modalities and their key mechanistic axes, we summarize current strategies, associated cancer types, and developmental stages (Table 4) to clarify translational progress and potential application pathways.

**TABLE 4:** Different cancer treatment strategies based on intratumoral bacteria.

Treatment means	Strategy	Model	Involving bacteria	Target cancer type	Effect	Phase of trial/identifier	Ref.
Engineered bacteria as carrier	By fusing the Fn cytoplasmic membrane with antibiotic‐loaded liposomes	Mice	Fn	Breast cancer	Significantly reversed the drug resistance of chemotherapy drugs and improved the curative effect of chemotherapy	/	[263]
	Designed a biomimetic nanovehicle for on‐site antibiotic delivery	Mice	Fn	Breast cancer	Improve the therapeutic effect of PD‐L1 blockers, inhibit tumor growth, and extend survival ITME	/	[264]
Bacteria‐derived nanomedicine (minicells)	Bacterial minicell drug delivery (EGFR‐targeted doxorubicin)	Human	Bacterially derived minicells (“EDV nanocells”) loaded with doxorubicin	Recurrent glioblastoma	Inhibit tumor growth; preliminary clinical activity with targeted delivery	Early‐phase/first‐in‐human	[265]
Engineered bacteria as immune enhancers	Release chemokines within tumors to attract adaptive immune cells into the tumor environment	Mice	Probiotic *Escherichia coli*	B‐cell lymphoma, CRC, breast cancers	Improve anti‐tumor immunity	/	[266]
	Associated ZIF‐8 metal—organic frameworks enclosing the eukaryotic murine Il2 expression plasmid are attached to the exterior of VNP20009	Mice	Attenuated *Salmonella typhimurium* strain	Melanoma, orthotopic hepatocellular carcinoma, and pulmonary metastasis models	Reactivate TIME, suppress tumor growth	/	[267]
	Exploiting delayed IL‐10 receptor signaling to evade phagocytosis and restore anti‐tumor immunity	Mice	*Salmonella enterica* (designer bacterium 1: DB1)	Solid tumors	Eliminate tumors, prevent recurrence, and inhibit metastasis of multiple tumor types	/	[268]
	Intratumoral synthetic biotic producing STING agonist; ± PD‐L1 blockade	Human	Human (first‐in‐human) (engineered)—SYNB1891	Advanced solid tumors/lymphoma	Improve anti‐tumor immunity (STING pathway activation; IFN‐stimulated gene induction); potential to improve response to PD‐(L)1 blockade	Phase 1; NCT04167137	[269]
	Systemic tumor‐targeting attenuated bacteria	Human	Attenuated *Salmonella typhimurium* (VNP20009)	Metastatic melanoma/RCC (advanced solid tumors)	Tumor‐targeting feasible; limited clinical efficacy	Phase 1	[270]
Engineered bacteria as vaccine	Utilizing *Salmonella* (Sal) engineered with CaCO_3_ biomineralization to enable localized cancer vaccine synthesis and modulate the ITME	Mice	*Salmonella typhimurium* strain	Melanoma tumor	Stimulate immune response, inhibit the growth of primary and metastatic tumors, and combine treatment with ICIs to improve efficacy	/	[271]
	Develop a polyvalent vaccine that encapsulates insoluble and soluble bacterial antigens to selectively target and eliminate harmful bacteria within tumors	Mice	Fn, *Streptococcus sanguis*, *Enterococcus faecalis*, *Staphylococcus xylosus*	Breast tumor	Stimulates strong antimicrobial immune response and inhibits tumor metastasis	/	[272]
	Oral *Salmonella*‐vector DNA vaccine targeting tumor vasculature (VEGFR2)	Human	Attenuated *Salmonella typhi* Ty21a vector—VXM01	Advanced pancreatic cancer	Improve anti‐tumor immunity (VEGFR2‐specific responses) and inhibit tumor growth via anti‐angiogenic mechanism	Phase 1; NCT01486329	[273]
	Live‐attenuated *Listeria* vaccine expressing mesothelin; often used in prime–boost with GVAX ± checkpoint blockade	Human	Attenuated *Listeria monocytogenes*—CRS‐207	Metastatic PDAC	Improve anti‐tumor immunity and support vaccine‐priming/boosting strategy	Multiple Phase 2 programs; e.g., NCT03006302	[274, 275]
Oncolytic bacteria	Iintratumoral injection of C. novyi‐NT	Human	*C. novyi‐NT*	Treatment‐refractory solid tumor	Partial tumor lysis		[276]
	Isolating intratumoral anti‐tumor bacteria	Mice	A‐gyo, UN‐gyo, AUN	CRC, sarcoma, metastatic lung cancer, and extensive drug‐resistant breast cancer	Enhance immune response and prolong survival	/	[223]
	Using tumor‐isolated *Cutibacterium acnes*	Mice	*Cutibacterium acnes*	Colon carcinoma	Activate immune cells and destroy tumors	/	[80, 277]
Antibiotics	Use of multiple antibiotics to eliminate intra‐tumor bacterial flora in pancreatic cancer models	Mice	Microbiota	Pancreatic cancer	Enhance immune response and immunotherapy effectiveness	/	[80]
	Engineered a liposome‐encapsulated silver‐tinidazole nanocomposite (termed LipoAgTNZ) to eliminate tumor‐associated bacteria	Mice	Fn	CRC	Stimulate anti‐tumor immunity	/	[278]
	Eradication/depletion of tumor‐associated Fn	Human	Metronidazole (targets anaerobes incl. Fn)	Stage II/III CRC (postoperative)	Reduce intratumoral Fn; potentially improve chemotherapy efficacy/ITME	Phase 2/NCT04264676	NA
Bacteriophages	Irinotecan‐loaded dextran nanoparticles were chemically linked to azide‐modified phage	Mice	Fn	CRC	Augments their responses to chemotherapy	/	[279]
	λ‐Bacteriophage particle vaccine expressing tumor antigen	Human	Engineered λ‐bacteriophage vaccine (SNS‐301/PAN‐301‐1)	Prostate cancer (biochemical relapse; per trial)	Improve anti‐tumor immunity (tumor‐antigen–directed response)	Phase 1/NCT03120832	NA

Abbreviations: C. novyi‐NT, Clostridium novyi‐NT; CRC, colorectal cancer; CRS‐207, live‐attenuated Listeria monocytogenes vaccine strain expressing mesothelin; DB1, engineered Salmonella strain DB1; EDV, EnGeneIC Dream Vector; EGFR, epidermal growth factor receptor; Fn, *Fusobacterium nucleatum*; GVAX, granulocyte‐macrophage colony‐stimulating factor‐secreting tumor cell vaccine; ICI(s), immune checkpoint inhibitor(s); IFN, interferon; IFN‐I, type I interferon; IL‐10R, interleukin 10 receptor; IL‐12, interleukin 12; ITME, intratumoral microenvironment; LipoAgTNZ, liposome‐encapsulated silver‐tinidazole nanocomposite; NA, not available; NCT, ClinicalTrials.gov identifier; PAN‐301‐1, PAN‐301‐1 clinical study identifier; PD‐(L)1, programmed cell death protein 1/programmed death‐ligand 1 axis; PDAC, pancreatic ductal adenocarcinoma; PD‐L1, programmed death‐ligand 1; RCC, renal cell carcinoma; SNS‐301, engineered λ‐bacteriophage vaccine (SNS‐301); STING, stimulator of interferon genes; TIME, tumor immune microenvironment; VEGFR2, vascular endothelial growth factor receptor 2; VNP20009, attenuated Salmonella typhimurium strain VNP20009; VXM01, oral Salmonella Ty21a‐based VEGFR2 DNA vaccine.

### 10.1 Engineered Bacteria

Engineered bacteria, as “smart biological carriers,” have demonstrated substantial promise in tumor‐targeted therapy. A central advantage lies in the intrinsic ability of certain bacteria to colonize tumor tissues and to exhibit relative tolerance to immune clearance, providing a biological foundation for precision delivery systems [20]. For example, Chen and colleagues exploited the specific binding of the Fn membrane protein Fap2 to Gal‐GalNAc moieties on tumor cells to construct Fn‐membrane‐coated nanovehicles (Colistin‐LipoFM). This platform enabled targeted elimination of tumor‐colonizing bacteria, suppressed lung metastasis, restored chemosensitivity, and achieved complete tumor regression in subsets of mouse models [280].

Beyond bacterial depletion, engineered strains can be designed to remodel antitumor immunity through directed secretion of immunomodulatory factors. Savage and colleagues engineered bacteria to secrete CXCL16/CCL20, markedly enhancing recruitment of dendritic cells and CD8^+^ T cells to tumors, increasing tumor‐infiltrating lymphocyte density, and thereby improving immunotherapeutic efficacy [266]. More recently, an engineered *Salmonella* DB1 strain was reported to enhance the function of tumor‐resident CD8^+^ T cells via activation of IL‐10 receptor (IL‐10R) signaling, achieving tumor clearance, recurrence suppression, and blockade of metastasis across multiple tumor models [268].

In vaccine development, Guo and colleagues developed a calcium carbonate (CaCO_3_) biomineralized *Salmonella* vaccine capable of activating both local and systemic antitumor immunity and further improving efficacy when combined with immune checkpoint blockade [271]. Kang and colleagues designed a polyvalent vaccine carrying soluble and insoluble bacterial antigens to selectively eliminate multiple pro‐metastatic bacteria [272].

Notably, clinical translation of engineered bacteria still faces major challenges. Synthetic scaffolds and exogenous genes may trigger excessive immunogenicity or unintended inflammatory responses. In addition, horizontal gene transfer, genetic stability, and prevention of nonspecific colonization in healthy tissues represent substantial safety concerns. Achieving precise targeting of both primary tumors and metastatic lesions—while avoiding normal organs—remains a key optimization problem. These considerations highlight that, alongside therapeutic potential, biosafety and controllability must be rigorously evaluated before broader clinical deployment.

### 10.2 Probiotics

Probiotics have evolved from classical modulators of gut ecology into biologic tools that can directly shape antitumor immunity and enhance therapeutic efficacy. Multiple studies indicate that specific probiotic taxa can strengthen antitumor immune responses and improve immunotherapy outcomes. In a landmark study, Sivan et al. transferred the microbiota from JAX mice (highly responsive to anti‐PD‐L1 therapy) into TAC mice (poor responders), thereby markedly enhancing anti‐PD‐L1 efficacy against melanoma and identifying *Bifidobacterium* as a key functional taxon‐providing causal evidence that defined probiotic bacteria can determine immunotherapy responsiveness [250]. Subsequent independent studies across multiple models reached concordant conclusions, systematically supporting that microbiome interventions can broadly potentiate immunotherapy and establishing a conceptual basis for “probiotic–immunotherapy synergy” [186, 251]. On this basis, combining immunotherapy with live bacterial supplementation is considered a more targeted and controllable strategy, enabling precise modulation of the tumor immune microenvironment using one or more functionally defined microbes [254]. Mechanistically, probiotics can modulate antitumor immunity via microbial metabolites. For instance, *L. reuteri* produces I3A, which activates AhR signaling and enhances IFN‐γ production by CD8^+^ T cells, thereby improving ICI efficacy. In contrast, Fn‐derived succinate can suppress the cGAS‐IFN‐β pathway, limit CD8^+^ T‐cell infiltration, and weaken antitumor immunity, highlighting the functional antagonism between beneficial and pro‐tumor taxa along the metabolism‐immunity axis [171, 173]. In addition, probiotics may inhibit invasion and metastasis by suppressing bone marrow‐derived neutrophil production, downregulating MMP‐9 expression, and upregulating E‐cadherin, with demonstrable anti‐metastatic effects across multiple models [281, 282]. Clinically, probiotic‐based combination strategies have also shown encouraging signals. In metastatic renal cell carcinoma, *C. butyricum* CBM588 in combination with immunotherapy or targeted therapy improved treatment responses, suggesting potential clinical value for strain‐specific precision interventions [254, 283].

With continued advances in synthetic biology and materials science, probiotic applications are further expanding toward engineered and functionalized platforms. Engineered *E. coli* Nissle 1917 (EcN) can locally release target molecules recognizable by chimeric antigen receptor T (CAR‐T) cells, enabling spatially restricted CAR‐T activation and infiltration in solid tumors, thereby enhancing efficacy while reducing systemic toxicity [284, 285, 286]. Moreover, functionalizing probiotics with a gallium‐polyphenol network can enhance tumor enrichment, stability, and antitumor activity while preserving bacterial viability, providing a new engineering route toward multifunctional probiotic therapeutic platforms [287].

Overall, through immune potentiation, metabolic modulation, anti‐metastatic effects, and engineered delivery, probiotics are becoming an important nexus linking tumor‐associated ecology, immunotherapy, and precision oncology. Nevertheless, their effects remain strongly strain‐specific and tumor‐context dependent, warranting validation in larger, more finely stratified human cohorts [15].

### 10.3 Oncolytic Bacteria

Oncolytic bacteria refer to functional bacterial strains that possess intrinsic tumoricidal activity, and recent years have witnessed notable advances in both mechanistic understanding and therapeutic development. Goto and colleagues isolated and identified bacterial strains such as A‐gyo, UN‐gyo, and AUN from clinical specimens, demonstrating multifaceted antitumor effects. These bacteria not only activated multiple immune cell populations and promoted their infiltration into tumor tissues, but also directly induced tumor cell apoptosis and inhibited tumor growth [223]. In a murine model of melanoma lung metastasis, these oncolytic bacteria markedly suppressed metastatic lesion formation, suggesting that specific tumor‐resident bacteria may, under certain conditions, attenuate tumor invasiveness and metastatic potential.

In addition, Chintalapati et al. isolated *Cutibacterium acnes* from mouse tumors and showed that it exerts tumor‐suppressive effects by activating diverse immune cell subsets and inducing tumor cell apoptosis. This strain also secretes multiple degradative enzymes that disrupt tumor tissue structure, thereby further promoting tumor cell death. Compared with conventional anticancer drugs and ICIs, such oncolytic bacterial approaches may offer potential advantages in cost and safety, providing an additional therapeutic option for cancer treatment [277].

### 10.4 Antibiotics and Bacteriophages

Precision antimicrobial interventions targeting the tumor microbiome are increasingly emerging as an important branch of cancer therapy. Under the “driver‐passenger” framework, selective elimination of pro‐tumor microorganisms—such as Fn and toxigenic *B. fragilis*—may help reverse immunosuppressive microenvironments and suppress metastatic progression [288]. Current strategies largely fall into two categories: antibiotic‐based approaches and bacteriophage‐based therapies.

Antibiotics can rapidly deplete target taxa through either broad‐spectrum or targeted regimens. For example, Wang and colleagues developed the liposomal antibiotic LipoAgTNZ, which eradicates Fn, reverses immunosuppression, and blocks pro‐metastatic signaling pathways such as β‐catenin and NF‐κB, thereby inhibiting tumor metastasis [278]. Notably, Smruti and colleagues employed an antibiotic combination strategy—rather than phage therapy—in a pancreatic cancer model, showing that depletion of intratumoral microbes enhanced the efficacy of anti‐PD‐1 therapy [80]. However, broad‐spectrum antibiotics may simultaneously disrupt beneficial microbial communities, weaken antitumor immunity, and compromise immunotherapy responses; thus, their application requires careful risk‐benefit assessment.

In contrast, bacteriophage therapy has attracted attention because of its high specificity. For instance, Zheng et al. engineered bacteriophages targeting Fn and covalently conjugated them to irinotecan, thereby significantly augmenting chemotherapy efficacy in CRC models [279]. Although phage‐based strategies may, in principle, reduce collateral disruption of the broader microbiome, clinical translation remains challenged by multiple hurdles, including individualized phage preparation, quality control, long‐term safety evaluation, and regulatory approval. At present, these constraints limit the feasibility of broadly replacing antibiotics with bacteriophage therapy in routine clinical settings [289].

## 11 Other Components of the Intratumoral Microbiota: Fungi and Viruses

While intratumoral bacteria represent the most extensively characterized component of the tumor microbiota, emerging evidence indicates that fungi and viruses also contribute to tumor biology.

### 11.1 Fungal Components

Recent pan‐cancer mycobiome analyses have revealed fungal DNA signals across multiple tumor types, including gastrointestinal, breast, and lung cancers [2, 3]. Fungal signatures not only display tumor‐type specificity and correlate with immune infiltration and survival outcomes [2, 3] but, crucially, also engage in complex cross‐kingdom synergistic interactions with intratumoral bacteria to drive tumor progression [2, 290, 291, 292]. For instance, a recent study demonstrated that in CRC, *Candida albicans* directly binds to Fn via a Flo9‐RadD protein interaction. This physical cross‐kingdom “bridging” significantly enhances the mucosal colonization and oncogenic capacity of Fn, while disrupting this axis with L‐arginine effectively abrogates their synergistic effect. However, given the low biomass of fungal material in tumor tissues and susceptibility to environmental contamination, rigorous validation strategies remain essential to confirm biological relevance.

### 11.2 Viral Components

Oncogenic viruses constitute a well‐established category of tumor‐associated microorganisms. EBV, human papillomavirus (HPV), hepatitis B virus (HBV), and hepatitis C virus (HCV) are causally linked to specific malignancies [293]. Beyond classical viral oncogenesis, accumulating evidence suggests that intratumoral viral signals (including oncolytic virotherapy contexts) may influence immune checkpoint responses and the tumor microenvironment more broadly [294, 295]. Viral antigens can shape antitumor T‐cell repertoires, while persistent viral infection may sustain chronic inflammatory circuits that promote tumor progression [293].

Collectively, these findings suggest that intratumoral microbiota should be viewed as a multi‐kingdom ecosystem. Nevertheless, compared with bacterial research, mechanistic and quantitative insights into tumor‐associated fungi and viruses remain relatively limited. Future studies integrating multi‐omics, spatial profiling, and functional validation will be necessary to clarify their causal roles in tumor progression and metastasis.

## 12 Conclusion and Prospects

Intratumoral microbiome has been redefined from incidental bystanders to functional components of the tumor ecosystem. Across diverse cancer types, these microbes‐particularly intratumoral bacteria‐exhibit striking organ specificity, spatial heterogeneity, and functional diversity, and in specific contexts, they may connect with oral‐gut microbial reservoirs. Throughout the full tumor life cycle—from initiation and local progression to metastatic dissemination and therapeutic response—intratumoral bacteria profoundly influence tumor fate through multifaceted mechanisms [63, 292].

During early tumorigenesis, bacteria can promote DNA damage and mutational accumulation by producing genotoxins, inducing oxidative stress, and persisting within locally protected micro‐niches, while simultaneously accelerating malignant transformation through epigenetic remodeling and oncogenic signaling modulation. During tumor progression, they support tumor growth by sustaining chronic inflammatory signaling, remodeling the stromal environment, and modulating vascular function. Notably, despite the diversity of microbial taxa, their mechanisms of action converge on a limited set of host core programs, including biased DNA damage response, stress adaptation, immune checkpoint activation, and metabolic reprogramming. This “convergence within diversity” provides a theoretical foundation for developing broad‐spectrum intervention strategies [7, 63].

In the context of metastasis, intratumoral bacteria promote dissemination through two complementary routes: first, by enhancing tumor cell‐intrinsic metastatic capacity, including induction of EMT‐like programs, release of pro‐metastatic factors, transcriptional and epigenetic reprogramming, and increased tolerance of CTCs to fluid shear stress; second, by remodeling the extrinsic microenvironment through modulation of inflammatory‐immune ecology, conditioning of distant pre‐metastatic niches, and promotion of angiogenesis, thereby creating permissive “soil” for tumor cell colonization. This spatiotemporally dynamic, multi‐layered regulation positions targeting of microorganisms as a new entry point for metastasis intervention [32, 51].

Clinically, intratumoral bacteria hold dual significance. As biomarkers, microbial features capture functional states of the microenvironment not reflected by tumor genomics alone, complementing existing diagnostic and prognostic systems. As therapeutic modulators, they profoundly influence the efficacy of chemotherapy, radiotherapy, and immunotherapy through enzymatic drug metabolism, modulation of stress response programs, and remodeling of the immune landscape. The strong context dependence of these effects—whereby the same bacterial species may yield opposite clinical outcomes in different immune‐metabolic contexts—underscores that future research must dissect microbial function with spatiotemporal precision, rather than remaining at the level of static associations [7, 32].

Despite substantial progress, the field faces several critical challenges. First, the low‐biomass nature of intratumoral microbial signals necessitates stringent contamination control and standardized analytical workflows. Recent large‐scale sequencing studies have revealed a cautionary phenomenon: in the absence of rigorous host read depletion and robust decontamination procedures, “microbial signals” in low‐biomass tissues are highly susceptible to interference from reagent background, batch effects, and computational artifacts, yielding misleading associations [63, 64, 65]. Future studies must therefore establish rigorous methodological standards—from blank controls and batch effect correction to orthogonal validation. Second, most reported microbe‐tumor associations have not yet crossed the threshold of causal inference, necessitating a complete validation pipeline encompassing prospective cohorts, depletion/reconstitution models, strain‐level functional screening, and spatially informed perturbation experiments [7, 51]. Third, current work remains largely bacteria‐centric, yet the roles of fungi, viruses, and their cross‐kingdom interactions in tumors remain underexplored; our understanding of the tumor microecology may represent only the tip of the iceberg [63].

Addressing these challenges requires a fundamental shift from descriptive observation to hypothesis‐driven inquiry, which can be organized around four key directions [15, 63, 156, 170, 286]. First, it should be determined whether the spatial localization of intratumoral microorganisms—such as tumor‐cell‐proximal, stromal, immune, or hypoxic niches—predicts distinct biological programs and clinical outcomes, including immune exclusion, ECM remodeling, angiogenesis, and treatment response. Second, future work should test whether defined microbe‐derived metabolites engage specific host receptors or sensors to reset immune and stress‐response thresholds, thereby altering tumor growth dynamics and sensitivity to chemotherapy, radiotherapy, or immune checkpoint blockade. Third, it should be evaluated whether phylogenetically distinct microorganisms converge on a limited set of shared host stress programs—such as biased DNA damage responses, autophagy dependence, or antigen‐presentation constraints—which, if validated, could reveal cross‐context therapeutic vulnerabilities. Fourth, prospective studies should assess whether composite microbial signatures integrating taxonomic composition, functional genes, metabolomic profiles, and spatial organization can improve patient stratification beyond existing biomarkers and guide treatment optimization. In translational terms, these hypotheses support two complementary strategies: on the one hand, targeting pathogenic microbial functions or the microenvironments they shape—via antibiotics, bacteriophages, or metabolic pathway interception—to suppress tumor progression or therapeutic resistance; on the other hand, exploiting tumor‐tropic bacteria as programmable live drug vectors for localized delivery of immunomodulators, cytotoxic agents, or vaccine antigens, thereby reducing systemic toxicity [7, 20, 63].

Ultimately, with the standardization of low‐biomass workflows, the advancement of spatially resolved mechanistic studies, and the progression of prospective clinical validation, intratumoral microbiome research is moving from descriptive associations toward testable and targetable mechanistic frameworks. This evolution will not only deepen our understanding of tumor biology but also promise to provide microbiome‐informed solutions for cancer diagnosis, prognosis, and therapeutic optimization.

## Author Contributions

Jiawei Chen and Yupan Bai contributed equally to data collection, data curation, and formal analysis. Jiawei Chen performed the original draft writing. Yupan Bai performed the figure preparation. Lu Shen assisted with Tables 1 and 2 preparation. Jiacheng Ying assisted with manuscript revision of Sections 9 and 10. Jiayin Tang and Jie Xu were responsible for project supervision. Jiayin Tang was responsible for administration and funding acquisition. Yujie Bao was responsible for conceptualization, supervision, funding acquisition, and manuscript review and editing. All authors have read and approved the final manuscript.

## Funding

This work was supported by Shanghai Magnolia Talent Plan Pujiang Project (Award No. 24PJD053) and National Natural Science Foundation of China (Award Nos. 82002486 and 82273279).

## Ethics Statement

The authors have nothing to report.

## Conflicts of Interest

The authors declare no conflicts of interests.

## Supporting information




**Supplementary Table 1**. Intratumoral bacterial landscape across human cancers.

## Data Availability

No datasets were generated or analyzed during the current study.

## References

[1] D. Nejman , I. Livyatan , G. Fuks , et al., “The Human Tumor Microbiome Is Composed of Tumor Type‐Specific Intracellular Bacteria,” Science 368, no. 6494 (2020): 973–980. 32467386 10.1126/science.aay9189 PMC7757858

[2] L. Narunsky‐Haziza , G. D. Sepich‐Poore , I. Livyatan , et al., “Pan‐Cancer Analyses Reveal Cancer‐Type‐Specific Fungal Ecologies and Bacteriome Interactions,” Cell 185, no. 20 (2022): 3789–3806.e17. 36179670 10.1016/j.cell.2022.09.005 PMC9567272

[3] A. B. Dohlman , J. Klug , M. Mesko , et al., “A Pan‐Cancer Mycobiome Analysis Reveals Fungal Involvement in Gastrointestinal and Lung Tumors,” Cell 185, no. 20 (2022): 3807–3822.e12. 36179671 10.1016/j.cell.2022.09.015 PMC9564002

[4] Y. He , Q. Zhang , X. Yu , S. Zhang , and W. Guo , “Overview of Microbial Profiles in human Hepatocellular Carcinoma and Adjacent Nontumor Tissues,” Journal of Translational Medicine 21, no. 1 (2023): 68. 36732743 10.1186/s12967-023-03938-6 PMC9893660

[5] Z. Shi , H. Ren , C. Lin , et al., “Tissue‐Resident Microbiota Impacts Colorectal Cancer Progression and Prognosis,” Nature Communications 17, no. 1 (2025): 346. 10.1038/s41467-025-67047-2 PMC12789584 41354681

[6] Y. Cao , H. Xia , X. Tan , et al., “Intratumoural Microbiota: A New Frontier in Cancer Development and Therapy,” Signal Transduction and Targeted Therapy 9, no. 1 (2024): 15. 38195689 10.1038/s41392-023-01693-0 PMC10776793

[7] L. Yang , A. Li , Y. Wang , and Y. Zhang , “Intratumoral Microbiota: Roles in Cancer Initiation, Development and Therapeutic Efficacy,” Signal Transduction and Targeted Therapy 8, no. 1 (2023): 35. 36646684 10.1038/s41392-022-01304-4 PMC9842669

[8] C. Xue , X. Gu , Q. Shi , et al., “The Interaction Between Intratumoral Bacteria and Metabolic Distortion in Hepatocellular Carcinoma,” Journal of Translational Medicine 22, no. 1 (2024): 237. 38439045 10.1186/s12967-024-05036-7 PMC10910819

[9] A. W. Lambert , Y. Zhang , and R. A. Weinberg , “Cell‐Intrinsic and Microenvironmental Determinants of Metastatic Colonization,” Nature Cell Biology 26, no. 5 (2024): 687–697. 38714854 10.1038/s41556-024-01409-8

[10] K. Ganesh and J. Massagué , “Targeting Metastatic Cancer,” Nature Medicine 27, no. 1 (2021): 34–44. 10.1038/s41591-020-01195-4 PMC7895475 33442008

[11] S. Gerstberger , Q. Jiang , and K. Ganesh , “Metastasis,” Cell 186, no. 8 (2023): 1564–1579. 37059065 10.1016/j.cell.2023.03.003 PMC10511214

[12] R. L. Siegel , T. B. Kratzer , N. S. Wagle , H. Sung , and A. Jemal , “Cancer Statistics, 2026,” CA: A Cancer Journal for Clinicians 76, no. 1 (2026): e70043. 41528114 10.3322/caac.70043 PMC12798275

[13] K. Mani , D. Deng , C. Lin , M. Wang , M. L. Hsu , and N. G. Zaorsky , “Causes of Death Among People Living With Metastatic Cancer,” Nature Communications 15, no. 1 (2024): 1519. 10.1038/s41467-024-45307-x PMC10876661 38374318

[14] C. Xue , Q. Chu , Q. Zheng , et al., “Current Understanding of the Intratumoral Microbiome in Various Tumors,” Cell Reports Medicine 4, no. 1 (2023): 100884. 36652905 10.1016/j.xcrm.2022.100884 PMC9873978

[15] H. Zhang , L. Fu , X. Leiliang , et al., “Beyond the Gut: The Intratumoral Microbiome's Influence on Tumorigenesis and Treatment Response,” Cancer Communications 44, no. 10 (2024): 1130–1167. 39087354 10.1002/cac2.12597 PMC11483591

[16] A. Fu , B. Yao , T. Dong , and S. Cai , “Emerging Roles of Intratumor Microbiota in Cancer Metastasis,” Trends in Cell Biology 33, no. 7 (2023): 583–593. 36522234 10.1016/j.tcb.2022.11.007

[17] C. Kong , X. Yan , Y. Zhu , et al., “Fusobacterium Nucleatum Promotes the Development of Colorectal Cancer by Activating a Cytochrome P450/Epoxyoctadecenoic Acid Axis via TLR4/Keap1/NRF2 Signaling,” Cancer Research 81, no. 17 (2021): 4485–4498. 34162680 10.1158/0008-5472.CAN-21-0453

[18] D. Mouradov , P. Greenfield , S. Li , et al., “Oncomicrobial Community Profiling Identifies Clinicomolecular and Prognostic Subtypes of Colorectal Cancer,” Gastroenterology 165, no. 1 (2023): 104–120. 36933623 10.1053/j.gastro.2023.03.205

[19] S. Okuda , Y. Shimada , Y. Tajima , et al., “Profiling of Host Genetic Alterations and Intra‐Tumor Microbiomes in Colorectal Cancer,” Computational and Structural Biotechnology Journal 19 (2021): 3330–3338. 34188781 10.1016/j.csbj.2021.05.049 PMC8202188

[20] F. Peng , M. Hu , Z. Su , L. Hu , L. Guo , and K. Yang , “Intratumoral Microbiota as a Target for Advanced Cancer Therapeutics,” Advanced Materials 36, no. 38 (2024): e2405331. 39054925 10.1002/adma.202405331

[21] Z. Shi , Z. Li , and M. Zhang , “Emerging Roles of Intratumor Microbiota in Cancer: Tumorigenesis and Management Strategies,” Journal of Translational Medicine 22, no. 1 (2024): 837. 39261861 10.1186/s12967-024-05640-7 PMC11391643

[22] G. D. Sepich‐Poore , L. Zitvogel , R. Straussman , J. Hasty , J. A. Wargo , and R. Knight , “The Microbiome and Human Cancer,” Science 371, no. 6536 (2021): eabc4552. 33766858 10.1126/science.abc4552 PMC8767999

[23] S. J. Salter , M. J. Cox , E. M. Turek , et al., “Reagent and Laboratory Contamination Can Critically Impact Sequence‐Based Microbiome Analyses,” BMC Biology 12 (2014): 87. 25387460 10.1186/s12915-014-0087-z PMC4228153

[24] R. Eisenhofer , J. J. Minich , C. Marotz , A. Cooper , R. Knight , and L. S. Weyrich , “Contamination in Low Microbial Biomass Microbiome Studies: Issues and Recommendations,” Trends in Microbiology 27, no. 2 (2019): 105–117. 30497919 10.1016/j.tim.2018.11.003

[25] A. Gihawi , Y. Ge , J. Lu , et al., “Major Data Analysis Errors Invalidate Cancer Microbiome Findings,” MBio 14, no. 5 (2023): e0160723. 37811944 10.1128/mbio.01607-23 PMC10653788

[26] B. Wiemann and C. O. Starnes , “Coley's Toxins, Tumor Necrosis Factor and Cancer Research: A Historical Perspective,” Pharmacology & Therapeutics 64, no. 3 (1994): 529–564. 7724661 10.1016/0163-7258(94)90023-x

[27] S. A. Hoption Cann , J. P. van Netten , and C. van Netten , “Dr William Coley and Tumour Regression: A Place in History or in the Future,” Postgraduate Medical Journal 79, no. 938 (2003): 672–680. 14707241 PMC1742910

[28] P. Rous , “A Sarcoma of the Fowl Transmissible by an Agent Separable from the Tumor Cells,” Journal of Experimental Medicine 13, no. 4 (1911): 397–411. 19867421 10.1084/jem.13.4.397 PMC2124874

[29] M. A. Epstein , B. G. Achong , and Y. M. Barr , “Virus Particles in Cultured Lymphoblasts from Burkitt's Lymphoma,” Lancet 1, no. 7335 (1964): 702–703. 14107961 10.1016/s0140-6736(64)91524-7

[30] J. R. Warren and B. Marshall , “Unidentified Curved Bacilli on Gastric Epithelium in Active Chronic Gastritis,” Lancet 1, no. 8336 (1983): 1273–1275. 6134060

[31] W. Fischbach and P. Malfertheiner , “ *Helicobacter pylori* Infection,” Deutsches Arzteblatt International 115, no. 25 (2018): 429–436. 29999489 10.3238/arztebl.2018.0429 PMC6056709

[32] T. W. Battaglia , I. L. Mimpen , J. J. H. Traets , et al., “A Pan‐Cancer Analysis of the Microbiome in Metastatic Cancer,” Cell 187, no. 9 (2024): 2324–2335, e19. 38599211 10.1016/j.cell.2024.03.021

[33] J. L. Galeano Niño , H. Wu , and K. D. LaCourse , “Effect of the Intratumoral Microbiota on Spatial and Cellular Heterogeneity in Cancer,” Nature 611, no. 7937 (2022): 810–817. 36385528 10.1038/s41586-022-05435-0 PMC9684076

[34] S. Saarenpää , O. Shalev , H. Ashkenazy , et al., “Spatial Metatranscriptomics Resolves Host‐Bacteria‐Fungi Interactomes,” Nature Biotechnology 42, no. 9 (2024): 1384–1393. 10.1038/s41587-023-01979-2 PMC11392817 37985875

[35] W. Zhou , Q. Yang , J. Guo , et al., “SMTdb: A Comprehensive Spatial Meta‐Transcriptome Resource in Cancer,” Molecular Biology and Evolution 42, no. 11 (2025): msaf263. 41092484 10.1093/molbev/msaf263 PMC12596269

[36] J. B. Patel , “16S rRNA Gene Sequencing for Bacterial Pathogen Identification in the Clinical Laboratory,” Molecular Diagnosis 6, no. 4 (2001): 313–321. 11774196 10.1054/modi.2001.29158

[37] P. Yarza , P. Yilmaz , E. Pruesse , et al., “Uniting the Classification of Cultured and Uncultured Bacteria and Archaea Using 16S rRNA Gene Sequences,” Nature Reviews Microbiology 12, no. 9 (2014): 635–645. 25118885 10.1038/nrmicro3330

[38] C. Quince , A. W. Walker , J. T. Simpson , N. J. Loman , and N. Segata , “Corrigendum: Shotgun Metagenomics, From Sampling to Analysis,” Nature Biotechnology 35, no. 12 (2017): 1211. 10.1038/nbt1217-1211b 29220029

[39] A. Mukherjee and M. S. Reddy , “Metatranscriptomics: An Approach for Retrieving Novel Eukaryotic Genes From Polluted and Related Environments,” 3 Biotech 10, no. 2 (2020): 71. 10.1007/s13205-020-2057-1 PMC6985312 32030340

[40] Y. Xie , F. Xie , X. Zhou , et al., “Microbiota in Tumors: From Understanding to Application,” Advanced Science 9, no. 21 (2022): e2200470. 35603968 10.1002/advs.202200470 PMC9313476

[41] A. E. Budding , M. E. Grasman , F. Lin , et al., “IS‐pro: High‐Throughput Molecular Fingerprinting of the Intestinal Microbiota,” FASEB Journal: Official Publication of the Federation of American Societies for Experimental Biology 24, no. 11 (2010): 4556–4564. 20643909 10.1096/fj.10-156190

[42] C. J. F. Heymann , J. M. Bard , M. F. Heymann , D. Heymann , and C. Bobin‐Dubigeon , “The Intratumoral Microbiome: Characterization Methods and Functional Impact,” Cancer Letters 522 (2021): 63–79. 34517085 10.1016/j.canlet.2021.09.009

[43] A. D. Kostic , D. Gevers , C. S. Pedamallu , et al., “Genomic Analysis Identifies Association of Fusobacterium With Colorectal Carcinoma,” Genome Research 22, no. 2 (2012): 292–298. 22009990 10.1101/gr.126573.111 PMC3266036

[44] I. Pope , H. Tanner , F. Masia , et al., “Correlative Light‐Electron Microscopy Using Small Gold Nanoparticles as Single Probes,” Light, Science & Applications 12, no. 1 (2023): 80. 10.1038/s41377-023-01115-4 PMC10050153 36977682

[45] Z. Huang , S. Mo , L. Yan , et al., “A Simple Culture Method Enhances the Recovery of Culturable Actinobacteria from Coastal Sediments,” Frontiers in Microbiology 12 (2021): 675048. 34194410 10.3389/fmicb.2021.675048 PMC8236954

[46] W. H. Lewis , G. Tahon , P. Geesink , D. Z. Sousa , and T. J. G. Ettema , “Innovations to Culturing the Uncultured Microbial Majority,” Nature Reviews Microbiology 19, no. 4 (2021): 225–240. 33093661 10.1038/s41579-020-00458-8

[47] E. Gigi , N. Gavert , L. Raijman‐Nagar , et al., “Characterization of the Tumor Microbiome of Brain Metastases and Glioblastoma Reveals Tumor‐Type‐Specific and Location‐Specific Microbial Signatures,” Nature Cancer 6, no. 11 (2025): 1761–1776. 41238775 10.1038/s43018-025-01073-3

[48] X. Qian , H. Y. Zhang , Q. L. Li , et al., “Integrated Microbiome, Metabolome, and Proteome Analysis Identifies a Novel Interplay Among Commensal Bacteria, Metabolites and Candidate Targets in Non‐Small Cell Lung Cancer,” Clinical and Translational Medicine 12, no. 6 (2022): e947. 35735103 10.1002/ctm2.947 PMC9218934

[49] R. A. Alharbi , “Proteomics Approach and Techniques in Identification of Reliable Biomarkers for Diseases,” Saudi Journal of Biological Sciences 27, no. 3 (2020): 968–974. 32127776 10.1016/j.sjbs.2020.01.020 PMC7042613

[50] E. B. Daliri , S. Wei , D. H. Oh , and B. H. Lee , “The Human Microbiome and Metabolomics: Current Concepts and Applications,” Critical Reviews in Food Science and Nutrition 57, no. 16 (2017): 3565–3576. 27767329 10.1080/10408398.2016.1220913

[51] A. Fu , B. Yao , T. Dong , et al., “Tumor‐Resident Intracellular Microbiota Promotes Metastatic Colonization in Breast Cancer,” Cell 185, no. 8 (2022): 1356–1372, e26. 35395179 10.1016/j.cell.2022.02.027

[52] W. Liu , X. Zhang , H. Xu , et al., “Microbial Community Heterogeneity Within Colorectal Neoplasia and Its Correlation with Colorectal Carcinogenesis,” Gastroenterology 160, no. 7 (2021): 2395–2408. 33581124 10.1053/j.gastro.2021.02.020

[53] J. S. Johnson , D. J. Spakowicz , B. Y. Hong , et al., “Evaluation of 16S rRNA Gene Sequencing for Species and Strain‐Level Microbiome Analysis,” Nature Communications 10, no. 1 (2019): 5029. 10.1038/s41467-019-13036-1 PMC6834636 31695033

[54] J. E. Clarridge 3rd , “Impact of 16S rRNA Gene Sequence Analysis for Identification of Bacteria on Clinical Microbiology and Infectious Diseases,” Clinical Microbiology Reviews 17, no. 4 (2004): 840–862. 15489351 10.1128/CMR.17.4.840-862.2004 PMC523561

[55] X. Zhou , S. Kandalai , F. Hossain , and Q. Zheng , “Tumor Microbiome Metabolism: A Game Changer in Cancer Development and Therapy,” Frontiers in Oncology 12 (2022): 933407. 35936744 10.3389/fonc.2022.933407 PMC9351545

[56] N. Zhang , S. Kandalai , X. Zhou , F. Hossain , and Q. Zheng , “Applying Multi‐Omics Toward Tumor Microbiome Research,” iMeta 2, no. 1 (2023): e73. 38868335 10.1002/imt2.73 PMC10989946

[57] S. Zaki , D. M. Blau , J. M. Hughes , et al., “CDC Grand Rounds: Discovering New Diseases via Enhanced Partnership Between Public Health and Pathology Experts,” MMWR Morbidity and Mortality Weekly Report 63, no. 6 (2014): 121–126. 24522095 PMC4584867

[58] X. Chai , J. Wang , H. Li , et al., “Intratumor Microbiome Features Reveal Antitumor Potentials of Intrahepatic Cholangiocarcinoma,” Gut Microbes 15, no. 1 (2023): 2156255. 36563106 10.1080/19490976.2022.2156255 PMC9794006

[59] K. L. Cross , J. H. Campbell , M. Balachandran , et al., “Targeted Isolation and Cultivation of Uncultivated Bacteria by Reverse Genomics,” Nature Biotechnology 37, no. 11 (2019): 1314–1321. 10.1038/s41587-019-0260-6 PMC6858544 31570900

[60] J. Puschhof , C. Pleguezuelos‐Manzano , A. Martinez‐Silgado , et al., “Intestinal Organoid Cocultures With Microbes,” Nature Protocols 16, no. 10 (2021): 4633–4649. 34381208 10.1038/s41596-021-00589-z

[61] R. J. Thompson , H. G. Bouwer , D. A. Portnoy , and F. R. Frankel , “Pathogenicity and Immunogenicity of a Listeria monocytogenes Strain That Requires D‐Alanine for Growth,” Infection and Immunity 66, no. 8 (1998): 3552–3561. 9673233 10.1128/iai.66.8.3552-3561.1998 PMC108386

[62] S. M. Desmarais , M. A. De Pedro , F. Cava , and K. C. Huang , “Peptidoglycan at Its Peaks: How Chromatographic Analyses Can Reveal Bacterial Cell Wall Structure and Assembly,” Molecular Microbiology 89, no. 1 (2013): 1–13. 23679048 10.1111/mmi.12266 PMC3694805

[63] Y. Q. Lu , H. Qiao , X. R. Tan , and N. Liu , “Broadening Oncological Boundaries: The Intratumoral Microbiota,” Trends in Microbiology 32, no. 8 (2024): 807–822. 38310023 10.1016/j.tim.2024.01.007

[64] Y. Ge , J. Lu , D. Puiu , M. Revsine , and S. L. Salzberg , “Comprehensive Analysis of Microbial Content in Whole‐Genome Sequencing Samples From the Cancer Genome Atlas Project,” Science Translational Medicine 17, no. 814 (2025): eads6335. 40901923 10.1126/scitranslmed.ads6335 PMC12821378

[65] A. Gihawi , H. M. Wood , J. Clark , et al., “The Landscape of Microbial Associations in human Cancer,” Science Translational Medicine 17, no. 814 (2025): eads6166. 40901921 10.1126/scitranslmed.ads6166

[66] I. Cho and M. J. Blaser , “The human Microbiome: At the Interface of Health and Disease,” Nature Reviews Genetics 13, no. 4 (2012): 260–270. 10.1038/nrg3182 PMC3418802 22411464

[67] R. V. Purcell , J. Pearson , A. Aitchison , L. Dixon , F. A. Frizelle , and J. I. Keenan , “Colonization With Enterotoxigenic *Bacteroides fragilis* Is Associated With Early‐Stage Colorectal Neoplasia,” PLoS ONE 12, no. 2 (2017): e0171602. 28151975 10.1371/journal.pone.0171602 PMC5289627

[68] S. Yamamoto , H. Kinugasa , M. Hirai , et al., “Heterogeneous Distribution of *Fusobacterium nucleatum* in the Progression of Colorectal Cancer,” Journal of Gastroenterology and Hepatology 36, no. 7 (2021): 1869–1876. 33242360 10.1111/jgh.15361

[69] Z. Lin , W. Rao , Z. Xiang , et al., “Characteristics and Interplay of Esophageal Microbiota in Esophageal Squamous Cell Carcinoma,” BMC Cancer 22, no. 1 (2022): 696. 35739509 10.1186/s12885-022-09771-2 PMC9229141

[70] B. A. Peters , R. B. Hayes , C. Goparaju , C. Reid , H. I. Pass , and J. Ahn , “The Microbiome in Lung Cancer Tissue and Recurrence‐Free Survival,” Cancer Epidemiology, Biomarkers & Prevention 28, no. 4 (2019): 731–740. 10.1158/1055-9965.EPI-18-0966 PMC6449216 30733306

[71] J. Abed , J. E. Emgård , G. Zamir , et al., “Fap2 Mediates *Fusobacterium nucleatum* Colorectal Adenocarcinoma Enrichment by Binding to Tumor‐Expressed Gal‐GalNAc,” Cell Host & Microbe 20, no. 2 (2016): 215–225. 27512904 10.1016/j.chom.2016.07.006 PMC5465824

[72] L. Parhi , T. Alon‐Maimon , and A. Sol , “Breast Cancer Colonization by *Fusobacterium nucleatum* Accelerates Tumor Growth and Metastatic Progression,” Nature Communications 11, no. 1 (2020): 3259. 10.1038/s41467-020-16967-2 PMC7320135 32591509

[73] C. L. Murphy , M. Barrett , P. Pellanda , et al., “Mapping the Colorectal Tumor Microbiota,” Gut Microbes 13, no. 1 (2021): 1–10. 10.1080/19490976.2021.1920657 PMC8158024 34030582

[74] S. Bullman , C. S. Pedamallu , E. Sicinska , et al., “Analysis of Fusobacterium Persistence and Antibiotic Response in Colorectal Cancer,” Science 358, no. 6369 (2017): 1443–1448. 29170280 10.1126/science.aal5240 PMC5823247

[75] N. Agrawal , C. Bettegowda , I. Cheong , et al., “Bacteriolytic Therapy Can Generate a Potent Immune Response Against Experimental Tumors,” Proceedings of the National Academy of Sciences of the United States of America 101, no. 42 (2004): 15172–15177. 15471990 10.1073/pnas.0406242101 PMC523456

[76] R. W. Kasinskas and N. S. Forbes , “Salmonella Typhimurium Lacking Ribose Chemoreceptors Localize in Tumor Quiescence and Induce Apoptosis,” Cancer Research 67, no. 7 (2007): 3201–3209. 17409428 10.1158/0008-5472.CAN-06-2618

[77] N. S. Forbes , “Engineering the Perfect (Bacterial) Cancer Therapy,” Nature Reviews Cancer 10, no. 11 (2010): 785–794. 20944664 10.1038/nrc2934 PMC3756932

[78] X. Zhong , X. He , Y. Wang , et al., “Warburg Effect in Colorectal Cancer: The Emerging Roles in Tumor Microenvironment and Therapeutic Implications,” Journal of Hematology & Oncology 15, no. 1 (2022): 160. 36319992 10.1186/s13045-022-01358-5 PMC9628128

[79] K. Yazawa , M. Fujimori , T. Nakamura , et al., “Bifidobacterium Longum as a Delivery System for Gene Therapy of Chemically Induced Rat Mammary Tumors,” Breast Cancer Research and Treatment 66, no. 2 (2001): 165–170. 11437103 10.1023/a:1010644217648

[80] S. Pushalkar , M. Hundeyin , D. Daley , et al., “The Pancreatic Cancer Microbiome Promotes Oncogenesis by Induction of Innate and Adaptive Immune Suppression,” Cancer Discovery 8, no. 4 (2018): 403–416. 29567829 10.1158/2159-8290.CD-17-1134 PMC6225783

[81] B. Ghaddar , A. Biswas , C. Harris , et al., “Tumor Microbiome Links Cellular Programs and Immunity in Pancreatic Cancer,” Cancer Cell 40, no. 10 (2022): 1240–1253, e5. 36220074 10.1016/j.ccell.2022.09.009 PMC9556978

[82] L. T. Geller , M. Barzily‐Rokni , T. Danino , et al., “Potential Role of Intratumor Bacteria in Mediating Tumor Resistance to the Chemotherapeutic Drug Gemcitabine,” Science 357, no. 6356 (2017): 1156–1160. 28912244 10.1126/science.aah5043 PMC5727343

[83] S. J. Taylor , M. G. Winter , C. C. Gillis , et al., “Colonocyte‐derived Lactate Promotes *E. coli* Fitness in the Context of Inflammation‐Associated Gut Microbiota Dysbiosis,” Microbiome 10, no. 1 (2022): 200. 36434690 10.1186/s40168-022-01389-7 PMC9701030

[84] R. Holani , H. Bar‐Yoseph , Z. Krekhno , et al., “Bile Acid‐Induced Metabolic Changes in the Colon Promote Enterobacteriaceae Expansion and Associate With Dysbiosis in Crohn's Disease,” Science Signaling 17, no. 867 (2024): eadl1786. 39689182 10.1126/scisignal.adl1786

[85] C. Clairmont , K. C. Lee , J. Pike , et al., “Biodistribution and Genetic Stability of the Novel Antitumor Agent VNP20009, a Genetically Modified Strain of Salmonella Typhimurium,” Journal of Infectious Diseases 181, no. 6 (2000): 1996–2002. 10837181 10.1086/315497

[86] N. L. Silver , J. Dai , T. D. Kerr , et al., “Intratumoral Bacteria Are Immunosuppressive and Promote Immunotherapy Resistance in Head and Neck Squamous Cell Carcinoma,” Nature Cancer 7, no. 1 (2026): 80–97. 41482523 10.1038/s43018-025-01067-1 PMC12823012

[87] A. B. Dohlman , D. Arguijo Mendoza , S. Ding , et al., “The Cancer Microbiome Atlas: A Pan‐Cancer Comparative Analysis to Distinguish Tissue‐Resident Microbiota From Contaminants,” Cell Host & Microbe 29, no. 2 (2021): 281–298.e5. 33382980 10.1016/j.chom.2020.12.001 PMC7878430

[88] K. Mima , R. Nishihara , Z. R. Qian , et al., “ *Fusobacterium nucleatum* in Colorectal Carcinoma Tissue and Patient Prognosis,” Gut 65, no. 12 (2016): 1973–1980. 26311717 10.1136/gutjnl-2015-310101 PMC4769120

[89] E. Riquelme , Y. Zhang , L. Zhang , et al., “Tumor Microbiome Diversity and Composition Influence Pancreatic Cancer Outcomes,” Cell 178, no. 4 (2019): 795–806.e12. 31398337 10.1016/j.cell.2019.07.008 PMC7288240

[90] M. R. Rubinstein , X. Wang , W. Liu , Y. Hao , G. Cai , and Y. W. Han , “ *Fusobacterium nucleatum* Promotes Colorectal Carcinogenesis by Modulating E‐Cadherin/β‐Catenin Signaling via Its FadA Adhesin,” Cell Host & Microbe 14, no. 2 (2013): 195–206. 23954158 10.1016/j.chom.2013.07.012 PMC3770529

[91] T. Yu , F. Guo , Y. Yu , et al., “ *Fusobacterium nucleatum* Promotes Chemoresistance to Colorectal Cancer by Modulating Autophagy,” Cell 170, no. 3 (2017): 548–563.e16. 28753429 10.1016/j.cell.2017.07.008 PMC5767127

[92] S. Zhang , J. Huang , Z. Jiang , H. Tong , X. Ma , and Y. Liu , “Tumor Microbiome: Roles in Tumor Initiation, Progression, and Therapy,” Molecular Biomedicine 6, no. 1 (2025): 9. 39921821 10.1186/s43556-025-00248-9 PMC11807048

[93] C. Pleguezuelos‐Manzano , J. Puschhof , A. Rosendahl Huber , et al., “Mutational Signature in Colorectal Cancer Caused by Genotoxic Pks(+) *E. coli* ,” Nature 580, no. 7802 (2020): 269–273. 32106218 10.1038/s41586-020-2080-8 PMC8142898

[94] J. P. Nougayrède , S. Homburg , F. Taieb , et al., “ *Escherichia coli* Induces DNA Double‐strand Breaks in Eukaryotic Cells,” Science 313, no. 5788 (2006): 848–851. 16902142 10.1126/science.1127059

[95] J. C. Arthur , E. Perez‐Chanona , M. Mühlbauer , et al., “Intestinal Inflammation Targets Cancer‐Inducing Activity of the Microbiota,” Science 338, no. 6103 (2012): 120–123. 22903521 10.1126/science.1224820 PMC3645302

[96] Z. R. Li , J. Li , W. Cai , et al., “Macrocyclic Colibactin Induces DNA Double‐Strand Breaks via Copper‐Mediated Oxidative Cleavage,” Nature Chemistry 11, no. 10 (2019): 880–889. 10.1038/s41557-019-0317-7 PMC6761029 31527851

[97] F. Geng , Y. Zhang , Z. Lu , S. Zhang , and Y. Pan , “ *Fusobacterium nucleatum* Caused DNA Damage and Promoted Cell Proliferation by the Ku70/p53 Pathway in Oral Cancer Cells,” DNA and Cell Biology 39, no. 1 (2020): 144–151. 31765243 10.1089/dna.2019.5064 PMC6978777

[98] A. Boleij , E. M. Hechenbleikner , A. C. Goodwin , et al., “The *Bacteroides fragilis* Toxin Gene Is Prevalent in the Colon Mucosa of Colorectal Cancer Patients,” Clinical Infectious Diseases 60, no. 2 (2015): 208–215. 25305284 10.1093/cid/ciu787 PMC4351371

[99] Z. He , R. Z. Gharaibeh , R. C. Newsome , et al., “ *Campylobacter jejuni* Promotes Colorectal Tumorigenesis Through the Action of Cytolethal Distending Toxin,” Gut 68, no. 2 (2019): 289–300. 30377189 10.1136/gutjnl-2018-317200 PMC6352414

[100] A. C. Goodwin , C. E. Destefano Shields , and S. Wu , “Polyamine Catabolism Contributes to Enterotoxigenic *Bacteroides fragilis*‐Induced Colon Tumorigenesis,” Proceedings of the National Academy of Sciences of the United States of America 108, no. 37 (2011): 15354–15359. 21876161 10.1073/pnas.1010203108 PMC3174648

[101] C. C. Wong and J. Yu , “Gut Microbiota in Colorectal Cancer Development and Therapy,” Nature Reviews Clinical Oncology 20, no. 7 (2023): 429–452. 10.1038/s41571-023-00766-x 37169888

[102] C. Xue , J. Jia , X. Gu , et al., “Intratumoral Bacteria Interact With Metabolites and Genetic Alterations in Hepatocellular Carcinoma,” Signal Transduction and Targeted Therapy 7, no. 1 (2022): 335. 36167691 10.1038/s41392-022-01159-9 PMC9515207

[103] H. E. Park , J. H. Kim , N. Y. Cho , H. S. Lee , and G. H. Kang , “Intratumoral *Fusobacterium nucleatum* Abundance Correlates With Macrophage Infiltration and CDKN2A Methylation in Microsatellite‐Unstable Colorectal Carcinoma,” Virchows Archiv: An International Journal of Pathology 471, no. 3 (2017): 329–336. 28597080 10.1007/s00428-017-2171-6

[104] P. H. Park , K. Keith , G. Calendo , et al., “Association Between Gut Microbiota and CpG Island Methylator Phenotype in Colorectal Cancer,” Gut Microbes 16, no. 1 (2024): 2363012. 38860458 10.1080/19490976.2024.2363012 PMC11174071

[105] A. A. Vega , E. A. Marshall , A. J. C. Noonan , et al., “Methionine‐Producing Tumor Micro(be) Environment Fuels Growth of Solid Tumors,” Cellular Oncology 46, no. 6 (2023): 1659–1673. 37318751 10.1007/s13402-023-00832-7 PMC10697899

[106] S. Chen , L. Zhang , M. Li , et al., “ *Fusobacterium nucleatum* Reduces METTL3‐Mediated M(6)A Modification and Contributes to Colorectal Cancer Metastasis,” Nature Communications 13, no. 1 (2022): 1248. 10.1038/s41467-022-28913-5 PMC8913623 35273176

[107] R. Lu , S. Wu , Y. G. Zhang , et al., “Enteric Bacterial Protein AvrA Promotes Colonic Tumorigenesis and Activates Colonic Beta‐Catenin Signaling Pathway,” Oncogenesis 3, no. 6 (2014): e105. 24911876 10.1038/oncsis.2014.20 PMC4150214

[108] R. Lu , X. Liu , S. Wu , et al., “Consistent Activation of the β‐Catenin Pathway by Salmonella Type—Three Secretion Effector Protein AvrA in Chronically Infected Intestine,” American Journal of Physiology Gastrointestinal and Liver Physiology 303, no. 10 (2012): G1113–G1125. 22982337 10.1152/ajpgi.00453.2011 PMC3517655

[109] G. Hoxhaj and B. D. Manning , “The PI3K‐AKT Network at the Interface of Oncogenic Signalling and Cancer Metabolism,” Nature Reviews Cancer 20, no. 2 (2020): 74–88. 31686003 10.1038/s41568-019-0216-7 PMC7314312

[110] F. Rascio , F. Spadaccino , M. T. Rocchetti , et al., “The Pathogenic Role of PI3K/AKT Pathway in Cancer Onset and Drug Resistance: An Updated Review,” Cancers 13, no. 16 (2021): 3949. 34439105 10.3390/cancers13163949 PMC8394096

[111] M. Martini , M. C. De Santis , L. Braccini , F. Gulluni , and E. Hirsch , “PI3K/AKT Signaling Pathway and Cancer: An Updated Review,” Annals of Medicine 46, no. 6 (2014): 372–383. 24897931 10.3109/07853890.2014.912836

[112] J. J. Tsay , B. G. Wu , M. H. Badri , et al., “Airway Microbiota Is Associated With Upregulation of the PI3K Pathway in Lung Cancer,” American Journal of Respiratory and Critical Care Medicine 198, no. 9 (2018): 1188–1198. 29864375 10.1164/rccm.201710-2118OC PMC6221574

[113] I. Chattopadhyay , M. Verma , and M. Panda , “Role of Oral Microbiome Signatures in Diagnosis and Prognosis of Oral Cancer,” Technology in Cancer Research & Treatment 18 (2019): 1533033819867354. 31370775 10.1177/1533033819867354 PMC6676258

[114] A. Martin‐Vega and M. H. Cobb , “ERK1/2‐MAPK Signaling: Metabolic, Organellar, and Cytoskeletal Interactions,” Current Opinion in Cell Biology 95 (2025): 102526. 40344863 10.1016/j.ceb.2025.102526

[115] D. A. Fruman , H. Chiu , B. D. Hopkins , S. Bagrodia , L. C. Cantley , and R. T. Abraham , “The PI3K Pathway in Human Disease,” Cell 170, no. 4 (2017): 605–635. 28802037 10.1016/j.cell.2017.07.029 PMC5726441

[116] J. Gnanasekaran , A. Binder Gallimidi , and E. Saba , “Intracellular *Porphyromonas gingivalis* Promotes the Tumorigenic Behavior of Pancreatic Carcinoma Cells,” Cancers 12, no. 8 (2020): 2331. 32824786 10.3390/cancers12082331 PMC7465784

[117] W. Mu , Y. Jia , X. Chen , H. Li , Z. Wang , and B. Cheng , “Intracellular *Porphyromonas gingivalis* Promotes the Proliferation of Colorectal Cancer Cells via the MAPK/ERK Signaling Pathway,” Frontiers in Cellular and Infection Microbiology 10 (2020): 584798. 33425779 10.3389/fcimb.2020.584798 PMC7785964

[118] G. N. Barber , “STING: Infection, Inflammation and Cancer,” Nature Reviews Immunology 15, no. 12 (2015): 760–770. 10.1038/nri3921 PMC5004891 26603901

[119] Y. Shi , W. Zheng , K. Yang , et al., “Intratumoral Accumulation of Gut Microbiota Facilitates CD47‐Based Immunotherapy via STING Signaling,” Journal of Experimental Medicine 217, no. 5 (2020): e20192282. 32142585 10.1084/jem.20192282 PMC7201921

[120] H. Zhao , L. Wu , G. Yan , et al., “Inflammation and Tumor Progression: Signaling Pathways and Targeted Intervention,” Signal Transduction and Targeted Therapy 6, no. 1 (2021): 263. 34248142 10.1038/s41392-021-00658-5 PMC8273155

[121] T. Duan , Y. Du , C. Xing , H. Y. Wang , and R. F. Wang , “Toll‐Like Receptor Signaling and Its Role in Cell‐Mediated Immunity,” Frontiers in Immunology 13 (2022): 812774. 35309296 10.3389/fimmu.2022.812774 PMC8927970

[122] Y. Yang , W. Weng , J. Peng , et al., “ *Fusobacterium nucleatum* Increases Proliferation of Colorectal Cancer Cells and Tumor Development in Mice by Activating Toll‐Like Receptor 4 Signaling to Nuclear Factor‐κB, and Up‐Regulating Expression of MicroRNA‐21,” Gastroenterology 152, no. 4 (2017): 851–866.e24. 27876571 10.1053/j.gastro.2016.11.018 PMC5555435

[123] S. Wu , K. C. Lim , J. Huang , R. F. Saidi , and C. L. Sears , “ *Bacteroides fragilis* Enterotoxin Cleaves the Zonula Adherens Protein, E‐Cadherin,” Proceedings of the National Academy of Sciences of the United States of America 95, no. 25 (1998): 14979–14984. 9844001 10.1073/pnas.95.25.14979 PMC24561

[124] L. Chung , E. Thiele Orberg , and A. L. Geis , “ *Bacteroides fragilis* Toxin Coordinates a Pro‐Carcinogenic Inflammatory Cascade via Targeting of Colonic Epithelial Cells,” Cell Host & Microbe 23, no. 2 (2018): 203–214, e5. 29398651 10.1016/j.chom.2018.01.007 PMC5954996

[125] A. Rivas‐Domínguez , N. Pastor , L. Martínez‐López , J. Colón‐Pérez , B. Bermúdez , and M. L. Orta , “The Role of DNA Damage Response in Dysbiosis‐Induced Colorectal Cancer,” Cells 10, no. 8 (2021): 1934. 34440703 10.3390/cells10081934 PMC8391204

[126] J. Katz , M. D. Onate , K. M. Pauley , I. Bhattacharyya , and S. Cha , “Presence of *Porphyromonas gingivalis* in Gingival Squamous Cell Carcinoma,” International Journal of Oral Science 3, no. 4 (2011): 209–215. 22010579 10.4248/IJOS11075 PMC3469978

[127] M. Castaneda , P. den Hollander , N. A. Kuburich , J. M. Rosen , and S. A. Mani , “Mechanisms of Cancer Metastasis,” Seminars in Cancer Biology 87 (2022): 17–31. 36354098 10.1016/j.semcancer.2022.10.006

[128] X. Guan , “Cancer Metastases: Challenges and Opportunities,” Acta Pharmaceutica Sinica B 5, no. 5 (2015): 402–418. 26579471 10.1016/j.apsb.2015.07.005 PMC4629446

[129] Y. Huang , W. Hong , and X. Wei , “The Molecular Mechanisms and Therapeutic Strategies of EMT in Tumor Progression and Metastasis,” Journal of Hematology & Oncology 15, no. 1 (2022): 129. 36076302 10.1186/s13045-022-01347-8 PMC9461252

[130] D. Hanahan and R. A. Weinberg , “Hallmarks of Cancer: The Next Generation,” Cell 144, no. 5 (2011): 646–674. 21376230 10.1016/j.cell.2011.02.013

[131] Y. Wu , S. Guo , F. Chen , et al., “Fn‐Dps, a Novel Virulence Factor of *Fusobacterium nucleatum*, Disrupts Erythrocytes and Promotes Metastasis in Colorectal Cancer,” PLoS Pathogens 19, no. 1 (2023): e1011096. 36693067 10.1371/journal.ppat.1011096 PMC9873182

[132] M. Hayashi , N. Ikenaga , K. Nakata , et al., “Intratumor *Fusobacterium nucleatum* Promotes the Progression of Pancreatic Cancer via the CXCL1‐CXCR2 Axis,” Cancer Science 114, no. 9 (2023): 3666–3678. 37438965 10.1111/cas.15901 PMC10475786

[133] S. Zhang , C. Li , J. Liu , et al., “Fusobacterium nucleatum Promotes Epithelial‐Mesenchymal Transition Through Regulation of the lncRNA MIR4435‐2HG/miR‐296‐5p/Akt2/SNAI1 Signaling Pathway,” FEBS Journal 287, no. 18 (2020): 4032–4047. 31997506 10.1111/febs.15233 PMC7540502

[134] G. Chen , C. Gao , S. Jiang , et al., “Fusobacterium nucleatum Outer Membrane Vesicles Activate Autophagy to Promote Oral Cancer Metastasis,” Journal of Advanced Research 56 (2024): 167–179. 37059221 10.1016/j.jare.2023.04.002 PMC10834801

[135] E. Bessède , C. Staedel , L. A. Acuña Amador , et al., “ *Helicobacter pylori* Generates Cells With Cancer Stem Cell Properties via Epithelial‐Mesenchymal Transition‐Like Changes,” Oncogene 33, no. 32 (2014): 4123–4131. 24096479 10.1038/onc.2013.380

[136] R. Roy , J. Yang , and M. A. Moses , “Matrix Metalloproteinases as Novel Biomarkers and Potential Therapeutic Targets in Human Cancer,” Journal of Clinical Oncology: Official Journal of the American Society of Clinical Oncology 27, no. 31 (2009): 5287–5297. 19738110 10.1200/JCO.2009.23.5556 PMC2773480

[137] S. Ou , H. Chen , H. Wang , et al., “Fusobacterium nucleatum Upregulates MMP7 to Promote Metastasis‐Related Characteristics of Colorectal Cancer Cell via Activating MAPK(JNK)‐AP1 Axis,” Journal of Translational Medicine 21, no. 1 (2023): 704. 37814323 10.1186/s12967-023-04527-3 PMC10561506

[138] P. J. Bergin , E. Anders , W. Sicheng , et al., “Increased Production of Matrix Metalloproteinases in *Helicobacter pylori*‐Associated Human Gastritis,” Helicobacter 9, no. 3 (2004): 201–210. 15165255 10.1111/j.1083-4389.2004.00232.x

[139] Y. Ma , H. Chen , H. Li , et al., “Intratumor Microbiome‐Derived Butyrate Promotes Lung Cancer Metastasis,” Cell Reports Medicine 5, no. 4 (2024): 101488. 38565146 10.1016/j.xcrm.2024.101488 PMC11031379

[140] S. Parida , S. Wu , S. Siddharth , et al., “A Procarcinogenic Colon Microbe Promotes Breast Tumorigenesis and Metastatic Progression and Concomitantly Activates Notch and β‐Catenin Axes,” Cancer Discovery 11, no. 5 (2021): 1138–1157. 33408241 10.1158/2159-8290.CD-20-0537

[141] Z. He , J. Yu , J. Gong , et al., “ *Campylobacter Jejuni*‐Derived Cytolethal Distending Toxin Promotes Colorectal Cancer Metastasis,” Cell Host & Microbe 32, no. 12 (2024): 2080–2091. 39626677 10.1016/j.chom.2024.11.006

[142] Q. Q. Liu , C. M. Li , L. N. Fu , et al., “Enterotoxigenic *Bacteroides fragilis* Induces the Stemness in Colorectal Cancer via Upregulating Histone Demethylase JMJD2B,” Gut Microbes 12, no. 1 (2020): 1788900. 32684087 10.1080/19490976.2020.1788900 PMC7524313

[143] S. Guo , F. Chen , L. Li , et al., “Intracellular *Fusobacterium nucleatum* Infection Increases METTL3‐Mediated m6A Methylation to Promote the Metastasis of Esophageal Squamous Cell Carcinoma,” Journal of Advanced Research 61 (2024): 165–178. 37619934 10.1016/j.jare.2023.08.014 PMC11258656

[144] Y. Zhang , L. Zhang , S. Zheng , et al., “ *Fusobacterium nucleatum* Promotes Colorectal Cancer Cells Adhesion to Endothelial Cells and Facilitates Extravasation and Metastasis by Inducing ALPK1/NF‐κB/ICAM1 Axis,” Gut Microbes 14, no. 1 (2022): 2038852. 35220887 10.1080/19490976.2022.2038852 PMC8890384

[145] F. Gao , B. Yu , B. Rao , et al., “The Effect of the Intratumoral Microbiome on Tumor Occurrence, Progression, Prognosis and Treatment,” Frontiers in Immunology 13 (2022): 1051987. 36466871 10.3389/fimmu.2022.1051987 PMC9718533

[146] Y. Cao , Z. Wang , Y. Yan , et al., “Enterotoxigenic *Bacteroides fragilis* Promotes Intestinal Inflammation and Malignancy by Inhibiting Exosome‐Packaged miR‐149‐3p,” Gastroenterology 161, no. 5 (2021): 1552–1566.e12. 34371001 10.1053/j.gastro.2021.08.003

[147] S. Guo , J. Chen , F. Chen , et al., “Exosomes Derived From Fusobacterium Nucleatum‐Infected Colorectal Cancer Cells Facilitate Tumour Metastasis by Selectively Carrying miR‐1246/92b‐3p/27a‐3p and CXCL16,” Gut 70 (2021): 1507–1519. 10.1136/gutjnl-2020-321187 33172926

[148] R. Domenis , A. Cifù , D. Marinò , et al., “Toll‐Like Receptor‐4 Activation Boosts the Immunosuppressive Properties of Tumor Cells‐Derived Exosomes,” Scientific Reports 9, no. 1 (2019): 8457. 31186484 10.1038/s41598-019-44949-y PMC6560033

[149] Y. Murota and C. Jobin , “Bacteria Break Barrier to Promote Metastasis,” Cancer Cell 39, no. 5 (2021): 598–600. 33891891 10.1016/j.ccell.2021.03.009 PMC8982444

[150] A. Bertocchi , S. Carloni , P. S. Ravenda , et al., “Gut Vascular Barrier Impairment Leads to Intestinal Bacteria Dissemination and Colorectal Cancer Metastasis to Liver,” Cancer Cell 39, no. 5 (2021): 708–724.e11. 33798472 10.1016/j.ccell.2021.03.004

[151] M. Singhal and H. G. Augustin , “Beyond Angiogenesis: Exploiting Angiocrine Factors to Restrict Tumor Progression and Metastasis,” Cancer Research 80, no. 4 (2020): 659–662. 31831463 10.1158/0008-5472.CAN-19-3351

[152] A. Schirbel , S. Kessler , F. Rieder , et al., “Pro‐Angiogenic Activity of TLRs and NLRs: A Novel Link Between Gut Microbiota and Intestinal Angiogenesis,” Gastroenterology 144, no. 3 (2013): 613–623.e9. 23149220 10.1053/j.gastro.2012.11.005 PMC3578104

[153] X. Song , Y. An , and D. Chen , “Microbial Metabolite Deoxycholic Acid Promotes Vasculogenic Mimicry Formation in Intestinal Carcinogenesis,” Cancer Science 113, no. 2 (2022): 459–477. 34811848 10.1111/cas.15208 PMC8819290

[154] R. V. Purcell , J. Permain , and J. I. Keenan , “Enterotoxigenic *Bacteroides fragilis* Activates IL‐8 Expression Through Stat3 in Colorectal Cancer Cells,” Gut Pathogens 14, no. 1 (2022): 16. 35468857 10.1186/s13099-022-00489-x PMC9036718

[155] J. Xu , Y. Tian , B. Zhao , et al., “Gut Microbiome Influences Efficacy of Endostatin Combined With PD‐1 Blockade Against Colorectal Cancer,” Molecular Biomedicine 5, no. 1 (2024): 37. 39251538 10.1186/s43556-024-00200-3 PMC11383918

[156] T. Zhang , Y. Li , E. Zhai , et al., “Intratumoral *Fusobacterium nucleatum* Recruits Tumor‐Associated Neutrophils to Promote Gastric Cancer Progression and Immune Evasion,” Cancer Research 85, no. 10 (2025): 1819–1841. 39992708 10.1158/0008-5472.CAN-24-2580 PMC12079103

[157] Y. Lu , L. Xu , W. Chen , et al., “Intrahepatic Microbial Heterogeneity in Multifocal Hepatocellular Carcinoma and Its Association With Host Genomic and Transcriptomic Alterations,” Cancer Discovery 15, no. 8 (2025): 1630–1648. 40287964 10.1158/2159-8290.CD-24-1259 PMC12319405

[158] B. M. Szczerba , F. Castro‐Giner , M. Vetter , et al., “Neutrophils Escort Circulating Tumour Cells to Enable Cell Cycle Progression,” Nature 566, no. 7745 (2019): 553–557. 30728496 10.1038/s41586-019-0915-y

[159] C. Hu , L. Long , J. Lou , et al., “CTC‐Neutrophil Interaction: A Key Driver and Therapeutic Target of Cancer Metastasis,” Biomedicine & Pharmacotherapy 180 (2024): 117474. 39316968 10.1016/j.biopha.2024.117474

[160] P. Shen , P. Cheng , Y. Li , et al., “Unveiling the Covert Interaction Between Gut Microbiota and Neutrophils to Drive Colorectal Cancer Metastasis,” European Journal of Pharmacology 962 (2024): 176217. 38036200 10.1016/j.ejphar.2023.176217

[161] L. Yuan , L. Pan , Y. Wang , et al., “Characterization of the Landscape of the Intratumoral Microbiota Reveals That *Streptococcus anginosus* Increases the Risk of Gastric Cancer Initiation and Progression,” Cell Discovery 10, no. 1 (2024): 117. 39587089 10.1038/s41421-024-00746-0 PMC11589709

[162] C. Gur , Y. Ibrahim , B. Isaacson , et al., “Binding of the Fap2 Protein of *Fusobacterium nucleatum* to Human Inhibitory Receptor TIGIT Protects Tumors From Immune Cell Attack,” Immunity 42, no. 2 (2015): 344–355. 25680274 10.1016/j.immuni.2015.01.010 PMC4361732

[163] B. Udayasuryan , R. N. Ahmad , T. T. D. Nguyen , et al., “ *Fusobacterium nucleatum* Induces Proliferation and Migration in Pancreatic Cancer Cells Through Host Autocrine and Paracrine Signaling,” Science Signaling 15, no. 756 (2022): eabn4948. 36256708 10.1126/scisignal.abn4948 PMC9732933

[164] R. Peng , S. Liu , W. You , et al., “Gastric Microbiome Alterations Are Associated With Decreased CD8+ Tissue‐Resident Memory T Cells in the Tumor Microenvironment of Gastric Cancer,” Cancer Immunology Research 10, no. 10 (2022): 1224–1240. 35881964 10.1158/2326-6066.CIR-22-0107

[165] Z. C. Guo , S. L. Jing , X. Y. Jia , et al., “ *Porphyromonas gingivalis* Promotes the Progression of Oral Squamous Cell Carcinoma by Stimulating the Release of Neutrophil Extracellular Traps in the Tumor Immune Microenvironment,” Inflammation Research: Official Journal of the European Histamine Research Society 73, no. 5 (2024): 693–705. 10.1007/s00011-023-01822-z 38150024

[166] H. Wu , X. Leng , Q. Liu , et al., “Intratumoral Microbiota Composition Regulates Chemoimmunotherapy Response in Esophageal Squamous Cell Carcinoma,” Cancer Research 83, no. 18 (2023): 3131–3144. 37433041 10.1158/0008-5472.CAN-22-2593

[167] G. Zhu , H. Su , C. H. Johnson , S. A. Khan , H. Kluger , and L. Lu , “Intratumour Microbiome Associated With the Infiltration of Cytotoxic CD8+ T Cells and Patient Survival in Cutaneous Melanoma,” European Journal of Cancer 151 (2021): 25–34. 33962358 10.1016/j.ejca.2021.03.053 PMC8184628

[168] M. Xie , K. Yuan , Y. Zhang , et al., “Tumor‐Resident Probiotic *Clostridium Butyricum* Improves aPD‐1 Efficacy in Colorectal Cancer Models by Inhibiting IL‐6‐Mediated Immunosuppression,” Cancer Cell 43, no. 10 (2025): 1885–1901.e10. 40780216 10.1016/j.ccell.2025.07.012

[169] X. Wang , Y. Fang , W. Liang , et al., “ *Fusobacterium nucleatum* Facilitates Anti‐PD‐1 Therapy in Microsatellite Stable Colorectal Cancer,” Cancer Cell 42, no. 10 (2024): 1729–1746, e8. 39303724 10.1016/j.ccell.2024.08.019

[170] J. Chen , Y. Gao , Y. Chen , et al., “Identification and Validation of Intratumoral Microbiome Associated With Sensitization to Immune Checkpoint Inhibitors,” Cell Reports Medicine 6, no. 9 (2025): 102306. 40865517 10.1016/j.xcrm.2025.102306 PMC12490247

[171] S. S. Jiang , Y. L. Xie , X. Y. Xiao , et al., “ *Fusobacterium nucleatum*‐Derived Succinic Acid Induces Tumor Resistance to Immunotherapy in Colorectal Cancer,” Cell Host & Microbe 31, no. 5 (2023): 781–797.e9. 37130518 10.1016/j.chom.2023.04.010

[172] J. Gu , X. Xu , X. Li , et al., “Tumor‐Resident Microbiota Contributes to Colorectal Cancer Liver Metastasis by Lactylation and Immune Modulation,” Oncogene 43, no. 31 (2024): 2389–2404. 38890429 10.1038/s41388-024-03080-7 PMC11281901

[173] M. J. Bender , A. C. McPherson , C. M. Phelps , et al., “Dietary Tryptophan Metabolite Released by Intratumoral *Lactobacillus reuteri* Facilitates Immune Checkpoint Inhibitor Treatment,” Cell 186, no. 9 (2023): 1846–1862, e26. 37028428 10.1016/j.cell.2023.03.011 PMC10148916

[174] J. Zhu , S. Qin , R. Gu , S. Ji , G. Wu , and K. Gu , “Amuc_1434 From Akkermansia Muciniphila Enhances CD8+ T Cell‐Mediated Anti‐Tumor Immunity by Suppressing PD‐L1 in Colorectal Cancer,” FASEB Journal: Official Publication of the Federation of American Societies for Experimental Biology 39, no. 8 (2025): e70540. 40231387 10.1096/fj.202403295RR

[175] Z. Zhu , J. Cai , W. Hou , et al., “Microbiome and Spatially Resolved Metabolomics Analysis Reveal the Anticancer Role of Gut *Akkermansia muciniphila* by Crosstalk With Intratumoral Microbiota and Reprogramming Tumoral Metabolism in Mice,” Gut Microbes 15, no. 1 (2023): 2166700. 36740846 10.1080/19490976.2023.2166700 PMC9904296

[176] J. Ma , L. Huang , D. Hu , S. Zeng , Y. Han , and H. Shen , “The Role of the Tumor Microbe Microenvironment in the Tumor Immune Microenvironment: Bystander, Activator, or Inhibitor?,” Journal of Experimental & Clinical Cancer Research: CR 40, no. 1 (2021): 327. 34656142 10.1186/s13046-021-02128-w PMC8520212

[177] N. Singh , A. Gurav , S. Sivaprakasam , et al., “Activation of Gpr109a, Receptor for Niacin and the Commensal Metabolite Butyrate, Suppresses Colonic Inflammation and Carcinogenesis,” Immunity 40, no. 1 (2014): 128–139. 24412617 10.1016/j.immuni.2013.12.007 PMC4305274

[178] J. C. Drobner , B. J. Lichtbroun , E. A. Singer , and S. Ghodoussipour , “Examining the Role of Microbiota‐Centered Interventions in Cancer Therapeutics: Applications for Urothelial Carcinoma,” Technology in Cancer Research & Treatment 22 (2023): 15330338231164196. 36938621 10.1177/15330338231164196 PMC10028658

[179] X. Kang , C. Liu , Y. Ding , et al., “ *Roseburia intestinalis* Generated Butyrate Boosts Anti‐PD‐1 Efficacy in Colorectal Cancer by Activating Cytotoxic CD8(+) T Cells,” Gut 72, no. 11 (2023): 2112–2122. 37491158 10.1136/gutjnl-2023-330291 PMC10579466

[180] C. Lu , Y. Liu , N. M. Ali , B. Zhang , and X. Cui , “The Role of Innate Immune Cells in the Tumor Microenvironment and Research Progress in Anti‐tumor Therapy,” Frontiers in Immunology 13 (2022): 1039260. 36741415 10.3389/fimmu.2022.1039260 PMC9893925

[181] Q. Li , C. Ding , T. Meng , et al., “Butyrate Suppresses Motility of Colorectal Cancer Cells via Deactivating Akt/ERK Signaling in Histone Deacetylase Dependent Manner,” Journal of Pharmacological Sciences 135, no. 4 (2017): 148–155. 29233468 10.1016/j.jphs.2017.11.004

[182] L. M. Sipe , M. Chaib , A. K. Pingili , J. F. Pierre , and L. Makowski , “Microbiome, Bile Acids, and Obesity: How Microbially Modified Metabolites Shape Anti‐Tumor Immunity,” Immunological Reviews 295, no. 1 (2020): 220–239. 32320071 10.1111/imr.12856 PMC7841960

[183] S. Xia , D. Jia , and L. Wang , “Microbiota: A Dawn for Cancer Metastasis Therapy,” Trends in Molecular Medicine (2025): 458–471. 41062341 10.1016/j.molmed.2025.09.008

[184] C. Li , X. Xing , M. Li , et al., “Bile Acids Produced by Gut Microbiota Activate TGR5 to Promote Colorectal Liver Metastasis Progression by Inducing MDSCs Infiltration in Liver,” International Immunopharmacology 158 (2025): 114829. 40367692 10.1016/j.intimp.2025.114829

[185] T. Wang , J. N. R. Gnanaprakasam , X. Chen , et al., “Inosine Is an Alternative Carbon Source for CD8(+)‐T‐Cell Function Under Glucose Restriction,” Nature Metabolism 2, no. 7 (2020): 635–647. 10.1038/s42255-020-0219-4 PMC7371628 32694789

[186] L. F. Mager , R. Burkhard , N. Pett , et al., “Microbiome‐Derived Inosine Modulates Response to Checkpoint Inhibitor Immunotherapy,” Science 369, no. 6510 (2020): 1481–1489. 32792462 10.1126/science.abc3421

[187] W. Li , W. Shangguan , W. Huang , et al., “Gut *Parabacteroides distasonis*‐Derived Indole‐3‐Acetic Acid Promotes Phospholipid Remodeling and Enhances Ferroptosis Sensitivity via the AhR‐FASN Axis in Bladder Cancer,” Advanced Science 12, no. 34 (2025): e04688. 40557796 10.1002/advs.202504688 PMC12442663

[188] K. Hezaveh , R. S. Shinde , A. Klötgen , et al., “Tryptophan‐Derived Microbial Metabolites Activate the Aryl Hydrocarbon Receptor in Tumor‐Associated Macrophages to Suppress Anti‐Tumor Immunity,” Immunity 55, no. 2 (2022): 324–340, e8. 35139353 10.1016/j.immuni.2022.01.006 PMC8888129

[189] Q. Zhang , Q. Zhao , T. Li , et al., “ *Lactobacillus plantarum*‐Derived Indole‐3‐lactic Acid Ameliorates Colorectal Tumorigenesis via Epigenetic Regulation of CD8(+) T Cell Immunity,” Cell Metabolism 35, no. 6 (2023): 943–960.e9. 37192617 10.1016/j.cmet.2023.04.015

[190] L. E. Colbert , M. B. El Alam , R. Wang , et al., “Tumor‐Resident *Lactobacillus iners* Confer Chemoradiation Resistance Through Lactate‐Induced Metabolic Rewiring,” Cancer Cell 41, no. 11 (2023): 1945–1962.e11. 37863066 10.1016/j.ccell.2023.09.012 PMC10841640

[191] H. Yu , Y. Du , Y. He , et al., “Lactate Production by Tumor‐Resident Staphylococcus Promotes Metastatic Colonization in Lung Adenocarcinoma,” Cell Host & Microbe 33, no. 7 (2025): 1089–1105, e7. 40639336 10.1016/j.chom.2025.06.013

[192] K. Vinasco , H. M. Mitchell , N. O. Kaakoush , and N. Castaño‐Rodríguez , “Microbial Carcinogenesis: Lactic Acid Bacteria in Gastric Cancer,” Biochimica et Biophysica Acta Reviews on Cancer 1872, no. 2 (2019): 188309. 31394110 10.1016/j.bbcan.2019.07.004

[193] X. Pan , F. Ye , P. Ning , et al., “Structures of G‐Protein Coupled Receptor HCAR1 in Complex With Gi1 Protein Reveal the Mechanistic Basis for Ligand Recognition and Agonist Selectivity,” PLoS Biology 23, no. 4 (2025): e3003126. 40233099 10.1371/journal.pbio.3003126 PMC12040280

[194] Y. S. Lee , T. Y. Kim , Y. Kim , et al., “Microbiota‐Derived Lactate Accelerates Intestinal Stem‐Cell‐Mediated Epithelial Development,” Cell Host & Microbe 24, no. 6 (2018): 833–846. 30543778 10.1016/j.chom.2018.11.002

[195] H. Wang , X. Rong , G. Zhao , et al., “The Microbial Metabolite Trimethylamine N‐oxide Promotes Antitumor Immunity in Triple‐Negative Breast Cancer,” Cell Metabolism 34, no. 4 (2022): 581–594, e8. 35278352 10.1016/j.cmet.2022.02.010

[196] K. C. Lam , R. E. Araya , A. Huang , et al., “Microbiota Triggers STING‐type I IFN‐Dependent Monocyte Reprogramming of the Tumor Microenvironment,” Cell 184, no. 21 (2021): 5338–5356, e21. 34624222 10.1016/j.cell.2021.09.019 PMC8650838

[197] G. Dalmasso , A. Cougnoux , T. Faïs , et al., “Colibactin‐Producing *Escherichia coli* Enhance Resistance to Chemotherapeutic Drugs by Promoting Epithelial to Mesenchymal Transition and Cancer Stem Cell Emergence,” Gut Microbes 16, no. 1 (2024): 2310215. 38374654 10.1080/19490976.2024.2310215 PMC10880512

[198] W. Ma , L. Zhang , W. Chen , et al., “Microbiota Enterotoxigenic *Bacteroides fragilis*‐Secreted BFT‐1 Promotes Breast Cancer Cell Stemness and Chemoresistance Through Its Functional Receptor NOD1,” Protein & Cell 15, no. 6 (2024): 419–440. 38437016 10.1093/procel/pwae005 PMC11131025

[199] S. Wu , P. J. Morin , D. Maouyo , and C. L. Sears , “ *Bacteroides fragilis* Enterotoxin Induces c‐Myc Expression and Cellular Proliferation,” Gastroenterology 124, no. 2 (2003): 392–400. 12557145 10.1053/gast.2003.50047

[200] Q. Tan , X. Ma , B. Yang , et al., “Periodontitis Pathogen *Porphyromonas gingivalis* Promotes Pancreatic Tumorigenesis via Neutrophil Elastase From Tumor‐Associated Neutrophils,” Gut Microbes 14, no. 1 (2022): 2073785. 35549648 10.1080/19490976.2022.2073785 PMC9116393

[201] J. Ren , X. Han , H. Lohner , et al., “ *P. gingivalis* Infection Upregulates PD‐L1 Expression on Dendritic Cells, Suppresses CD8+ T‐Cell Responses, and Aggravates Oral Cancer,” Cancer Immunology Research 11, no. 3 (2023): 290–305. 36633576 10.1158/2326-6066.CIR-22-0541 PMC9975670

[202] N. H. Ha , B. H. Woo , D. J. Kim , et al., “Prolonged and Repetitive Exposure to *Porphyromonas gingivalis* Increases Aggressiveness of Oral Cancer Cells by Promoting Acquisition of Cancer Stem Cell Properties,” Tumour Biology: The Journal of the International Society for Oncodevelopmental Biology and Medicine 36, no. 12 (2015): 9947–9960. 26178482 10.1007/s13277-015-3764-9

[203] A. Javaheri , T. Kruse , K. Moonens , et al., “ *Helicobacter pylori* Adhesin HopQ Engages in a Virulence‐Enhancing Interaction With Human CEACAMs,” Nature Microbiology 2 (2016): 16189. 10.1038/nmicrobiol.2016.189 27748768

[204] C. Gur , N. Maalouf , M. Gerhard , et al., “The *Helicobacter pylori* HopQ Outermembrane Protein Inhibits Immune Cell Activities,” Oncoimmunology 8, no. 4 (2019): e1553487. 30906650 10.1080/2162402X.2018.1553487 PMC6422397

[205] R. Luo , Y. Yao , Z. Chen , and X. Sun , “An Examination of the LPS‐TLR4 Immune Response Through the Analysis of Molecular Structures and Protein‐Protein Interactions,” Cell Communication and Signaling: CCS 23, no. 1 (2025): 142. 40102851 10.1186/s12964-025-02149-4 PMC11921546

[206] C. L. Dixon , A. Wu , and G. D. Fairn , “Multifaceted Roles and Regulation of Nucleotide‐Binding Oligomerization Domain Containing Proteins,” Frontiers in Immunology 14 (2023): 1242659. 37869013 10.3389/fimmu.2023.1242659 PMC10585062

[207] J. Karta , M. Meyers , F. Rodriguez , et al., “ *Fusobacterium nucleatum* Interacts With Cancer‐Associated Fibroblasts to Promote Colorectal Cancer,” EMBO Journal 44, no. 19 (2025): 5375–5393. 40846900 10.1038/s44318-025-00542-w PMC12488894

[208] X. He , J. Guo , Y. Bai , H. Sun , and J. Yang , “Salmonella‐Based Therapeutic Strategies: Improving Tumor Microenvironment and Bringing New Hope for Cancer Immunotherapy,” Medical Oncology 42, no. 1 (2024): 27. 39666238 10.1007/s12032-024-02578-0

[209] H. Wang , B. Lin , Z. Wang , et al., “ *Fusobacterium nucleatum* Increases CTGF Expression Through TLR2‐YAP Signaling Axis in Cancer‐Associated Fibroblasts, Thereby Promoting Colorectal Cancer Progression,” Cancer Cell International 25, no. 1 (2025): 381. 41152950 10.1186/s12935-025-04023-2 PMC12570683

[210] W. Cheng , Y. Liao , Y. Xie , et al., “ *Helicobacter pylori*‐Induced Fibroblast‐Derived Serpin E1 Promotes Gastric Cancer Growth and Peritoneal Dissemination Through p38 MAPK/VEGFA‐mediated Angiogenesis,” Cancer Cell International 23, no. 1 (2023): 326. 38104099 10.1186/s12935-023-03177-1 PMC10725580

[211] J. Li , X. Li , Z. Zhang , et al., “ *Helicobacter pylori* Promotes Gastric Fibroblast Proliferation and Migration by Expulsing Exosomal miR‐124‐3p,” Microbes and Infection 26, no. 1–2 (2024): 105236. 37813158 10.1016/j.micinf.2023.105236

[212] X. Long , C. C. Wong , L. Tong , et al., “Peptostreptococcus Anaerobius Promotes Colorectal Carcinogenesis and Modulates Tumour Immunity,” Nature Microbiology 4, no. 12 (2019): 2319–2330. 10.1038/s41564-019-0541-3 31501538

[213] S. Ruggiero , R. Cosgarea , J. Potempa , B. Potempa , S. Eick , and M. Chiquet , “Cleavage of Extracellular Matrix in Periodontitis: Gingipains Differentially Affect Cell Adhesion Activities of Fibronectin and Tenascin‐C,” Biochimica et Biophysica Acta 1832, no. 4 (2013): 517–526. 23313574 10.1016/j.bbadis.2013.01.003 PMC4188551

[214] X. Zhou , L. Chen , W. Lin , W. Zheng , H. Zhang , and F. Zhou , “Diagnostic and Prognostic Potential of the Intra‐Tumoral Microbiota Profile in HPV‐Independent Endocervical Adenocarcinoma,” Frontiers in Cellular and Infection Microbiology 14 (2024): 1440017. 39220287 10.3389/fcimb.2024.1440017 PMC11362085

[215] W. Wang , C. Qian , T. Wang , et al., “A Combination of Faecal and Intratumour Microbial Community Profiling Reveals Novel Diagnostic and Prognostic Biomarkers for Pancreatic Tumours,” Clinical and Translational Medicine 14, no. 6 (2024): e1726. 38822473 10.1002/ctm2.1726 PMC11142927

[216] X. Fan , A. V. Alekseyenko , J. Wu , et al., “Human Oral Microbiome and Prospective Risk for Pancreatic Cancer: A Population‐Based Nested Case‐Control Study,” Gut 67, no. 1 (2018): 120–127. 27742762 10.1136/gutjnl-2016-312580 PMC5607064

[217] L. Sun , X. Ke , A. Guan , et al., “Intratumoural Microbiome Can Predict the Prognosis of Hepatocellular Carcinoma After Surgery,” Clinical and Translational Medicine 13, no. 7 (2023): e1331. 37462602 10.1002/ctm2.1331 PMC10353526

[218] M. Hilmi , M. Kamal , S. Vacher , et al., “Intratumoral Microbiome Is Driven by Metastatic Site and Associated With Immune Histopathological Parameters: An Ancillary Study of the SHIVA Clinical Trial,” European Journal of Cancer 183 (2023): 152–161. 36868056 10.1016/j.ejca.2023.01.024

[219] H. Qiao , X. R. Tan , H. Li , et al., “Association of Intratumoral Microbiota with Prognosis in Patients With Nasopharyngeal Carcinoma From 2 Hospitals in China,” JAMA Oncology 8, no. 9 (2022): 1301–1309. 35834269 10.1001/jamaoncol.2022.2810 PMC9284409

[220] X.‐R. Tan , H. Qiao , Y.‐Q. Li , et al., “Tissue‐Resident Microbiota Signature in Nasopharyngeal Carcinoma,” Microbiome 13, no. 1 (2025): 125. 40382629 10.1186/s40168-025-02114-w PMC12085846

[221] D. Qu , Y. Wang , Q. Xia , J. Chang , X. Jiang , and H. Zhang , “Intratumoral Microbiome of Human Primary Liver Cancer,” Hepatology Communications 6, no. 7 (2022): 1741–1752. 35191218 10.1002/hep4.1908 PMC9234634

[222] S. Y. Kwon , H. Thi‐Thu Ngo , J. Son , Y. Hong , and J. J. Min , “Exploiting Bacteria for Cancer Immunotherapy,” Nature Reviews Clinical Oncology 21, no. 8 (2024): 569–589. 10.1038/s41571-024-00908-9 38840029

[223] Y. Goto , S. Iwata , M. Miyahara , and E. Miyako , “Discovery of Intratumoral Oncolytic Bacteria Toward Targeted Anticancer Theranostics,” Advanced Science 10, no. 20 (2023): e2301679. 37150857 10.1002/advs.202301679 PMC10369285

[224] S. Yan , T. Liu , H. Zhao , et al., “Colorectal Cancer‐Specific Microbiome in Peripheral Circulation and Cancer Tissues,” Frontiers in Microbiology 15 (2024): 1422536. 39234556 10.3389/fmicb.2024.1422536 PMC11371800

[225] H. J. An , M. A. Partha , H. Lee , et al., “Tumor‐Associated Microbiome Features of Metastatic Colorectal Cancer and Clinical Implications,” Frontiers in Oncology 13 (2023): 1310054. 38304032 10.3389/fonc.2023.1310054 PMC10833227

[226] G. Wang , H. Wang , X. Ji , et al., “Intratumoral Microbiome Is Associated With Gastric Cancer Prognosis and Therapy Efficacy,” Gut Microbes 16, no. 1 (2024): 2369336. 38944840 10.1080/19490976.2024.2369336 PMC11216101

[227] K. Yamamura , D. Izumi , R. Kandimalla , et al., “Intratumoral Fusobacterium Nucleatum Levels Predict Therapeutic Response to Neoadjuvant Chemotherapy in Esophageal Squamous Cell Carcinoma,” Clinical Cancer Research: An Official Journal of the American Association for Cancer Research 25, no. 20 (2019): 6170–6179. 31358543 10.1158/1078-0432.CCR-19-0318 PMC6801075

[228] S. T. Huang , J. Chen , and L. Y. Lian , “Intratumoral Levels and Prognostic Significance of *Fusobacterium nucleatum* in Cervical Carcinoma,” Aging 12, no. 22 (2020): 23337–23350. 33197886 10.18632/aging.104188 PMC7746363

[229] A. Pribluda , C. C. de la Cruz , and E. L. Jackson , “Intratumoral Heterogeneity: From Diversity Comes Resistance,” Clinical Cancer Research: An Official Journal of the American Association for Cancer Research 21, no. 13 (2015): 2916–2923. 25838394 10.1158/1078-0432.CCR-14-1213

[230] K. Khalaf , D. Hana , J. T. Chou , C. Singh , A. Mackiewicz , and M. Kaczmarek , “Aspects of the Tumor Microenvironment Involved in Immune Resistance and Drug Resistance,” Frontiers in Immunology 12 (2021): 656364. 34122412 10.3389/fimmu.2021.656364 PMC8190405

[231] A. Sevcikova , N. Izoldova , V. Stevurkova , et al., “The Impact of the Microbiome on Resistance to Cancer Treatment With Chemotherapeutic Agents and Immunotherapy,” International Journal of Molecular Sciences 23, no. 1 (2022): e83744. 10.3390/ijms23010488 PMC8745082 35008915

[232] C. Xuan , J. M. Shamonki , A. Chung , et al., “Microbial Dysbiosis Is Associated With human Breast Cancer,” PLoS ONE 9, no. 1 (2014): e83744. 24421902 10.1371/journal.pone.0083744 PMC3885448

[233] D. S. Michaud , J. Izard , C. S. Wilhelm‐Benartzi , et al., “Plasma Antibodies to Oral Bacteria and Risk of Pancreatic Cancer in a Large European Prospective Cohort Study,” Gut 62, no. 12 (2013): 1764–1770. 22990306 10.1136/gutjnl-2012-303006 PMC3815505

[234] C. Panebianco , A. Andriulli , and V. Pazienza , “Pharmacomicrobiomics: Exploiting the Drug‐microbiota Interactions in Anticancer Therapies,” Microbiome 6, no. 1 (2018): 92. 29789015 10.1186/s40168-018-0483-7 PMC5964925

[235] P. Lehouritis , J. Cummins , M. Stanton , et al., “Local Bacteria Affect the Efficacy of Chemotherapeutic Drugs,” Scientific Reports 5 (2015): 14554. 26416623 10.1038/srep14554 PMC4586607

[236] N. Wang , L. Zhang , X. X. Leng , et al., “ *Fusobacterium nucleatum* Induces Chemoresistance in Colorectal Cancer by Inhibiting Pyroptosis via the Hippo Pathway,” Gut Microbes 16, no. 1 (2024): 2333790. 38533566 10.1080/19490976.2024.2333790 PMC10978024

[237] B. Li , Z. Wei , Z. Wang , et al., “ *Fusobacterium nucleatum* Induces Oxaliplatin Resistance by Inhibiting Ferroptosis Through E‐Cadherin/β‐Catenin/GPX4 Axis in Colorectal Cancer,” Free Radical Biology & Medicine 220 (2024): 125–138. 38657754 10.1016/j.freeradbiomed.2024.04.226

[238] R. Daillère , M. Vétizou , N. Waldschmitt , et al., “Enterococcus Hirae and Barnesiella Intestinihominis Facilitate Cyclophosphamide‐Induced Therapeutic Immunomodulatory Effects,” Immunity 45, no. 4 (2016): 931–943. 27717798 10.1016/j.immuni.2016.09.009

[239] S. Viaud , F. Saccheri , G. Mignot , et al., “The Intestinal Microbiota Modulates the Anticancer Immune Effects of Cyclophosphamide,” Science 342, no. 6161 (2013): 971–976. 24264990 10.1126/science.1240537 PMC4048947

[240] J. Liu , C. Liu , and J. Yue , “Radiotherapy and the Gut Microbiome: Facts and Fiction,” Radiation Oncology 16, no. 1 (2021): 9. 33436010 10.1186/s13014-020-01735-9 PMC7805150

[241] G. Serna , F. Ruiz‐Pace , J. Hernando , et al., “ *Fusobacterium nucleatum* Persistence and Risk of Recurrence After Preoperative Treatment in Locally Advanced Rectal Cancer,” Annals of Oncology: Official Journal of the European Society for Medical Oncology 31, no. 10 (2020): 1366–1375. 32569727 10.1016/j.annonc.2020.06.003 PMC7542577

[242] K. Yang , Y. Hou , Y. Zhang , et al., “Suppression of Local Type I Interferon by Gut Microbiota‐Derived Butyrate Impairs Antitumor Effects of Ionizing Radiation,” Journal of Experimental Medicine 218, no. 3 (2021): e20201915. 33496784 10.1084/jem.20201915 PMC7844434

[243] X. Huang , C. Chen , W. Xie , et al., “Metagenomic Analysis of Intratumoral Microbiome Linking to Response to Neoadjuvant Chemoradiotherapy in Rectal Cancer,” International Journal of Radiation and Oncology in Biology and Physics 117, no. 5 (2023): 1255–1269. 10.1016/j.ijrobp.2023.06.2515 37433373

[244] J. Noguti , A. A. Chan , B. Bandera , et al., “Both the Intratumoral Immune and Microbial Microenvironment Are Linked to Recurrence in Human Colon Cancer: Results From a Prospective, Multicenter Nodal Ultrastaging Trial,” Oncotarget 9, no. 34 (2018): 23564–23576. 29805756 10.18632/oncotarget.25276 PMC5955112

[245] C. Neuzillet , M. Marchais , S. Vacher , et al., “Prognostic Value of Intratumoral *Fusobacterium nucleatum* and Association With Immune‐Related Gene Expression in Oral Squamous Cell Carcinoma Patients,” Scientific Reports 11, no. 1 (2021): 7870. 33846399 10.1038/s41598-021-86816-9 PMC8041800

[246] S. L. Shiao , K. M. Kershaw , J. J. Limon , et al., “Commensal Bacteria and Fungi Differentially Regulate Tumor Responses to Radiation Therapy,” Cancer Cell 39, no. 9 (2021): 1202–1213, e6. 34329585 10.1016/j.ccell.2021.07.002 PMC8830498

[247] H. Guo , W. C. Chou , Y. Lai , et al., “Multi‐Omics Analyses of Radiation Survivors Identify Radioprotective Microbes and Metabolites,” Science 370, no. 6516 (2020): eaay9097. 33122357 10.1126/science.aay9097 PMC7898465

[248] A. D. Waldman , J. M. Fritz , and M. J. Lenardo , “A Guide to Cancer Immunotherapy: From T Cell Basic Science to Clinical Practice,” Nature Reviews Immunology 20, no. 11 (2020): 651–668. 10.1038/s41577-020-0306-5 PMC7238960 32433532

[249] M. Vétizou , J. M. Pitt , R. Daillère , et al., “Anticancer Immunotherapy by CTLA‐4 Blockade Relies on the Gut Microbiota,” Science 350, no. 6264 (2015): 1079–1084. 26541610 10.1126/science.aad1329 PMC4721659

[250] A. Sivan , L. Corrales , N. Hubert , et al., “Commensal Bifidobacterium Promotes Antitumor Immunity and Facilitates Anti‐PD‐L1 Efficacy,” Science 350, no. 6264 (2015): 1084–1089. 26541606 10.1126/science.aac4255 PMC4873287

[251] B. Routy , E. Le Chatelier , and L. Derosa , “Gut Microbiome Influences Efficacy of PD‐1‐Based Immunotherapy Against Epithelial Tumors,” Science 359, no. 6371 (2018): 91–97. 29097494 10.1126/science.aan3706

[252] D. Davar , A. K. Dzutsev , J. A. McCulloch , et al., “Fecal Microbiota Transplant Overcomes Resistance to Anti‐PD‐1 Therapy in Melanoma Patients,” Science 371, no. 6529 (2021): 595–602. 33542131 10.1126/science.abf3363 PMC8097968

[253] E. N. Baruch , I. Youngster , G. Ben‐Betzalel , et al., “Fecal Microbiota Transplant Promotes Response in Immunotherapy‐Refractory Melanoma Patients,” Science 371, no. 6529 (2021): 602–609. 33303685 10.1126/science.abb5920

[254] N. Dizman , L. Meza , P. Bergerot , et al., “Nivolumab Plus Ipilimumab With or Without Live Bacterial Supplementation in Metastatic Renal Cell Carcinoma: A Randomized Phase 1 Trial,” Nature Medicine 28, no. 4 (2022): 704–712. 10.1038/s41591-022-01694-6 PMC9018425 35228755

[255] Z. Liu , X. Zhang , H. Zhang , et al., “Multi‐Omics Analysis Reveals Intratumor Microbes as Immunomodulators in Colorectal Cancer,” Microbiology Spectrum 11, no. 2 (2023): e0503822. 36786568 10.1128/spectrum.05038-22 PMC10100960

[256] Y. Li , S. Xing , F. Chen , et al., “Intracellular *Fusobacterium nucleatum* Infection Attenuates Antitumor Immunity in Esophageal Squamous Cell Carcinoma,” Nature Communications 14, no. 1 (2023): 5788. 10.1038/s41467-023-40987-3 PMC10507087 37723150

[257] F. Chen , J. Yang , Y. Guo , D. Su , Y. Sheng , and Y. Wu , “Integrating Bulk and Single‐Cell RNA Sequencing Data Reveals the Relationship Between Intratumor Microbiome Signature and Host Metabolic Heterogeneity in Breast Cancer,” Frontiers in Immunology 14 (2023): 1140995. 36999009 10.3389/fimmu.2023.1140995 PMC10049788

[258] A. Panda , J. M. Mehnert , K. M. Hirshfield , et al., “Immune Activation and Benefit from Avelumab in EBV‐Positive Gastric Cancer,” Journal of the National Cancer Institute 110, no. 3 (2018): 316–320. 29155997 10.1093/jnci/djx213 PMC6658862

[259] Y. Huang , N. Zhu , X. Zheng , et al., “Intratumor Microbiome Analysis Identifies Positive Association Between Megasphaera and Survival of Chinese Patients With Pancreatic Ductal Adenocarcinomas,” Frontiers in Immunology 13 (2022): 785422. 35145519 10.3389/fimmu.2022.785422 PMC8821101

[260] S. Chu , Z. Cheng , Z. Yin , et al., “Airway Fusobacterium Is Associated With Poor Response to Immunotherapy in Lung Cancer,” OncoTargets and Therapy 15 (2022): 201–213. 35250279 10.2147/OTT.S348382 PMC8896836

[261] L. Derosa , B. Routy , A. M. Thomas , et al., “Intestinal Akkermansia Muciniphila Predicts Clinical Response to PD‐1 Blockade in Patients With Advanced Non‐Small‐Cell Lung Cancer,” Nature Medicine 28, no. 2 (2022): 315–324. 10.1038/s41591-021-01655-5 PMC9330544 35115705

[262] J. S. Park , F. S. Gazzaniga , M. Wu , et al., “Targeting PD‐L2‐RGMb Overcomes Microbiome‐related Immunotherapy Resistance,” Nature 617, no. 7960 (2023): 377–385. 37138075 10.1038/s41586-023-06026-3 PMC10219577

[263] L. Chen , J. Shen , Z. Kang , et al., “ *Fusobacterium nucleatum*‐Mimicking Nanovehicles to Overcome Chemoresistance for Breast Cancer Treatment by Eliminating Tumor‐Colonizing Bacteria,” Chemistry 10, no. 6 (2024): 1783–1803.

[264] S. Geng , P. Guo , X. Li , et al., “Biomimetic Nanovehicle‐Enabled Targeted Depletion of Intratumoral *Fusobacterium nucleatum* Synergizes With PD‐L1 Blockade Against Breast Cancer,” ACS Nano 18, no. 12 (2024): 8971–8987. 38497600 10.1021/acsnano.3c12687

[265] J. R. Whittle , J. D. Lickliter , H. K. Gan , et al., “First in Human Nanotechnology Doxorubicin Delivery System to Target Epidermal Growth Factor Receptors in Recurrent Glioblastoma,” Journal of Clinical Neuroscience: Official Journal of the Neurosurgical Society of Australasia 22, no. 12 (2015): 1889–1894. 26279503 10.1016/j.jocn.2015.06.005

[266] T. M. Savage , R. L. Vincent , S. S. Rae , et al., “Chemokines Expressed by Engineered Bacteria Recruit and Orchestrate Antitumor Immunity,” Science Advances 9, no. 10 (2023): eadc9436. 36888717 10.1126/sciadv.adc9436 PMC9995032

[267] Y. Qiao , M. Luo , Y. Wang , et al., “Development of a Bacteria‐Nanosapper for the Active Delivery of ZIF‐8 Particles Containing Therapeutic Genes for Cancer Immune Therapy,” Acta Pharmaceutica Sinica B 14, no. 12 (2024): 5418–5434. 39807327 10.1016/j.apsb.2024.07.020 PMC11725029

[268] Z. Chang , X. Guo , X. Li , et al., “Bacterial Immunotherapy Leveraging IL‐10R Hysteresis for Both Phagocytosis Evasion and Tumor Immunity Revitalization,” Cell 188, no. 7 (2025): 1842–1857, e20. 40037354 10.1016/j.cell.2025.02.002

[269] J. J. Luke , S. A. Piha‐Paul , T. Medina , et al., “Phase I Study of SYNB1891, an Engineered *E. coli* Nissle Strain Expressing STING Agonist, With and Without Atezolizumab in Advanced Malignancies,” Clinical Cancer Research: An Official Journal of the American Association for Cancer Research 29, no. 13 (2023): 2435–2444. 37227176 10.1158/1078-0432.CCR-23-0118 PMC11225568

[270] J. F. Toso , V. J. Gill , P. Hwu , et al., “Phase I Study of the Intravenous Administration of Attenuated Salmonella Typhimurium to Patients With Metastatic Melanoma,” Journal of Clinical Oncology: Official Journal of the American Society of Clinical Oncology 20, no. 1 (2002): 142–152. 11773163 10.1200/JCO.2002.20.1.142 PMC2064865

[271] L. Guo , J. Ding , and W. Zhou , “Converting Bacteria Into Autologous Tumor Vaccine via Surface Biomineralization of Calcium Carbonate for Enhanced Immunotherapy,” Acta Pharmaceutica Sinica B 13, no. 12 (2023): 5074–5090. 38045045 10.1016/j.apsb.2023.08.028 PMC10692385

[272] Z. Kang , L. Chen , P. Li , et al., “A Polyvalent Vaccine for Selectively Killing Tumor‐Associated Bacteria to Prevent Cancer Metastasis,” Science Advances 11, no. 11 (2025): eadt0341. 40085697 10.1126/sciadv.adt0341 PMC11908479

[273] F. H. Schmitz‐Winnenthal , N. Hohmann , A. G. Niethammer , et al., “Anti‐Angiogenic Activity of VXM01, an Oral T‐Cell Vaccine Against VEGF Receptor 2, in Patients With Advanced Pancreatic Cancer: A Randomized, Placebo‐Controlled, Phase 1 Trial,” Oncoimmunology 4, no. 4 (2015): e1001217. 26137397 10.1080/2162402X.2014.1001217 PMC4485742

[274] D. T. Le , A. Wang‐Gillam , V. Picozzi , et al., “Safety and Survival With GVAX Pancreas Prime and Listeria Monocytogenes‐Expressing Mesothelin (CRS‐207) Boost Vaccines for Metastatic Pancreatic Cancer,” Journal of Clinical Oncology: Official Journal of the American Society of Clinical Oncology 33, no. 12 (2015): 1325–1333. 25584002 10.1200/JCO.2014.57.4244 PMC4397277

[275] T. Tsujikawa , T. Crocenzi , J. N. Durham , et al., “Evaluation of Cyclophosphamide/GVAX Pancreas Followed by Listeria‐Mesothelin (CRS‐207) With or Without Nivolumab in Patients With Pancreatic Cancer,” Clinical Cancer Research: An Official Journal of the American Association for Cancer Research 26, no. 14 (2020): 3578–3588. 32273276 10.1158/1078-0432.CCR-19-3978 PMC7727397

[276] F. Janku , H. H. Zhang , A. Pezeshki , et al., “Intratumoral Injection of Clostridium Novyi‐NT Spores in Patients With Treatment‐Refractory Advanced Solid Tumors,” Clinical Cancer Research: An Official Journal of the American Association for Cancer Research 27, no. 1 (2021): 96–106. 33046513 10.1158/1078-0432.CCR-20-2065

[277] S. Chintalapati , S. Iwata , M. Miyahara , and E. Miyako , “Tumor‐Isolated *Cutibacterium acnes* as an Effective Tumor Suppressive Living Drug,” Biomedicine & Pharmacotherapy 170 (2024): 116041. 38113626 10.1016/j.biopha.2023.116041

[278] M. Wang , B. Rousseau , K. Qiu , et al., “Killing Tumor‐Associated Bacteria With a Liposomal Antibiotic Generates Neoantigens That Induce Anti‐Tumor Immune Responses,” Nature Biotechnology 42, no. 8 (2024): 1263–1274. 10.1038/s41587-023-01957-8 PMC12892252 37749267

[279] D. W. Zheng , X. Dong , P. Pan , et al., “Phage‐Guided Modulation of the Gut Microbiota of Mouse Models of Colorectal Cancer Augments Their Responses to Chemotherapy,” Nature Biomedical Engineering 3, no. 9 (2019): 717–728. 10.1038/s41551-019-0423-2 31332342

[280] H. Guo , “Interactions Between the Tumor Microbiota and Breast Cancer,” Frontiers in Cellular and Infection Microbiology 14 (2024): 1499203. 39926112 10.3389/fcimb.2024.1499203 PMC11802574

[281] S. Matsumoto , T. Hara , M. Nagaoka , et al., “A Component of Polysaccharide Peptidoglycan Complex on Lactobacillus Induced an Improvement of Murine Model of Inflammatory Bowel Disease and Colitis‐Associated Cancer,” Immunology 128 (2009): e170–e180. 19740306 10.1111/j.1365-2567.2008.02942.x PMC2753921

[282] X. Li , H. Wang , X. Du , et al., “Lactobacilli Inhibit Cervical Cancer Cell Migration in Vitro and Reduce Tumor Burden in Vivo Through Upregulation of E‐Cadherin,” Oncology Reports 38, no. 3 (2017): 1561–1568. 28713905 10.3892/or.2017.5791

[283] H. Ebrahimi , N. Dizman , L. Meza , et al., “Cabozantinib and Nivolumab With or Without Live Bacterial Supplementation in Metastatic Renal Cell Carcinoma: A Randomized Phase 1 Trial,” Nature Medicine 30, no. 9 (2024): 2576–2585. 10.1038/s41591-024-03086-4 PMC11405272 38942995

[284] R. L. Vincent , C. R. Gurbatri , F. Li , et al., “Probiotic‐Guided CAR‐T Cells for Solid Tumor Targeting,” Science 382, no. 6667 (2023): 211–218. 37824640 10.1126/science.add7034 PMC10915968

[285] N. Aggarwal , A. M. E. Breedon , C. M. Davis , I. Y. Hwang , and M. W. Chang , “Engineering Probiotics for Therapeutic Applications: Recent Examples and Translational Outlook,” Current Opinion in Biotechnology 65 (2020): 171–179. 32304955 10.1016/j.copbio.2020.02.016

[286] W. Li , X. Ma , and X. Zhao , “Intratumoral Microorganisms and Artificial Antitumor Bacteria,” Fundamental Research 5, no. 5 (2025): 2045–2048. 41613448 10.1016/j.fmre.2023.06.002 PMC12848186

[287] Z. Y. Han , Z. J. Fu , Y. Z. Wang , et al., “Probiotics Functionalized With a Gallium‐Polyphenol Network Modulate the Intratumor Microbiota and Promote Anti‐Tumor Immune Responses in Pancreatic Cancer,” Nature Communications 15, no. 1 (2024): 7096. 10.1038/s41467-024-51534-z PMC11330462 39154092

[288] R. J. Knippel , J. L. Drewes , and C. L. Sears , “The Cancer Microbiome: Recent Highlights and Knowledge Gaps,” Cancer Discovery 11, no. 10 (2021): 2378–2395. 34400408 10.1158/2159-8290.CD-21-0324 PMC8487941

[289] M. Kabwe , S. Dashper , G. Bachrach , and J. Tucci , “Bacteriophage Manipulation of the Microbiome Associated With Tumour Microenvironments—Can This Improve Cancer Therapeutic Response?,” FEMS Microbiology Reviews 45, no. 5 (2021): fuab017. 33765142 10.1093/femsre/fuab017

[290] J. Li , C. Zhang , X. Huang , et al., “ *Candida albicans* Synergizes With Fusobacteriumnucleatum in Colorectal Cancer Progression via the Flo9‐RadD Interaction,” Cancer Cell (2026): 1063–1079.e8. 41759520 10.1016/j.ccell.2026.02.001

[291] B. Aykut , S. Pushalkar , R. Chen , et al., “The Fungal Mycobiome Promotes Pancreatic Oncogenesis via Activation of MBL,” Nature 574, no. 7777 (2019): 264–267. 31578522 10.1038/s41586-019-1608-2 PMC6858566

[292] N. N. Liu , C. X. Yi , L. Q. Wei , et al., “The Intratumor Mycobiome Promotes Lung Cancer Progression via Myeloid‐Derived Suppressor Cells,” Cancer Cell 42, no. 2 (2024): 318–322. 38350423 10.1016/j.ccell.2024.01.005

[293] J. J. Roetman , M. K. I. Apostolova , and M. Philip , “Viral and Cellular Oncogenes Promote Immune Evasion,” Oncogene 41, no. 7 (2022): 921–929. 35022539 10.1038/s41388-021-02145-1 PMC8851748

[294] S. Nakao , Y. Arai , M. Tasaki , et al., “Intratumoral Expression of IL‐7 and IL‐12 Using an Oncolytic Virus Increases Systemic Sensitivity to Immune Checkpoint Blockade,” Science Translational Medicine 12, no. 526 (2020): eaax7992. 31941828 10.1126/scitranslmed.aax7992

[295] T. T. Nguyen , D. H. Shin , S. Sohoni , et al., “Reshaping the Tumor Microenvironment With Oncolytic Viruses, Positive Regulation of the Immune Synapse, and Blockade of the Immunosuppressive Oncometabolic Circuitry,” Journal for ImmunoTherapy of Cancer 10, no. 7 (2022): e004935. 35902132 10.1136/jitc-2022-004935 PMC9341188

