# Supplementary material for: Intratumoral Microbiota in Tumor: Current Understandings and Future Perspectives
Source: MedComm (2020). 2026 Jun 23;7(7):e70825. doi: 10.1002/mco2.70825 (PMC13287974; doi:10.1002/mco2.70825)
Supplement: Supplementary file 1 — Supplementary Table 1. Intratumoral bacterial landscape across human cancers. [file MCO2-7-e70825-s001.docx]

**Intratumoral Microbiota in Tumor: Current Understandings and Future Perspectives**

Jiawei Chen^1#^, Yupan Bai^1#^, Lu Shen^1^, Jiacheng Ying^2^, Jie Xu^1^*, Jiayin Tang^2^*, Yujie Bao^1^*

^1^Department of Infectious Diseases, Shanghai Ninth People’s Hospital, Shanghai Jiao Tong University School of Medicine, Shanghai, 200011, China

^2^Department of Gastrointestinal Surgery, Renji Hospital, Shanghai Jiao Tong University School of Medicine, Shanghai, 200127, China

#These authors contributed equally:

Jiawei Chen, Email: jiaweichen@sjtu.edu.cn

Yupan Bai, Email: 116160@sh9hospital.org.cn

*Correspondence:

Yujie Bao, Department of Infectious Diseases, Shanghai Ninth People’s Hospital, Shanghai Jiao Tong University School of Medicine, Shanghai, 200011, China

Email: yujie.bao@shsmu.edu.cn

ORCID: 0000-0002-0674-4225

Jiayin Tang, Department of Gastrointestinal Surgery, Renji Hospital, Shanghai Jiao Tong University School of Medicine, Shanghai, 200127, China

Email: jiayintang@shsmu.edu.cn

ORCID: 0000-0002-2510-6448

Jie Xu, Department of Infectious Diseases, Shanghai Ninth People’s Hospital, Shanghai Jiao Tong University School of Medicine, Shanghai, 200011, China

Email: xujie@shsmu.edu.cn

ORCID: 0000-0002-9179-0049

**Supplementary table 1. Intratumoral bacterial landscape across human cancers**

| **Cancer types** | **Microbiome Composition and Abundance Change** | **Effect** | **Methods** | **Number of clinical samples** | **Associated mechanisms** | **Ref.** |
| --- | --- | --- | --- | --- | --- | --- |
| **Oral squamous cell carcinoma (OSCC)** | Enriched:  Fn  Decreased:  *Streptococcus pneumoniae* | Reshapes tumor microenvironment via attenuated anticancer secondary metabolite biosynthesis | 16S rRNA | 74 (discovery) + 42 (validation); male buccal mucosa cancer cohorts | / | ^1^ |
|  | Fn | Promotes OSCC cell proliferation and accelerates cell cycle. | 1. Anaerobic culture of Fn;  2. qPCR | N/A | Induces DNA damage and impairs repair mechanisms via Ku70 downregulation; concurrently, suppression of the Ku70/p53/p27 axis drives cell-cycle progression and OSCC proliferation. | ^2^ |
|  | Fn | 1. Promotes OSCC invasion and EMT progression;  2. Induces M2-like TAMs formation;  3. Enhances glycolysis and extracellular lactate deposition. | 1. 16S rRNA;  2. qPCR;  3FISH;  4. Anaerobic culture | 80 paired OSCC tumor and adjacent normal tissues | 1. Fn binds GalNAc and activates AKT/mTOR, inducing autophagy.  2.Autophagy downregulates TBC1D5, increasing GLUT1 membrane localization and glucose uptake.  3.Glycolysis increases lactate accumulation, promoting M2-like TAM polarization. | ^3^ |
|  | Enriched genera:  1. *Prevotella*  2.*Corynebacterium*  3. *Pseudomonas*  Depleted genera:  1. *Actinomyces*  2. *Sutterella*  3. *Stenotrophomonas* | 1.Induces oral microbiome dysbiosis and reduces bacterial diversity;  2.Enriches pathways related to nucleotide metabolism, DNA replication/repair, and protein translation. | 1. 16S rRNA;  2. qPCR | 50 paired OSCC tumors and matched adjacent normal tissues (Indian cohort) | 1. Enriched genera may promote proliferation by modulating metabolic and DNA repair pathways;  2. Dysbiosis and HPV-16 co-association may synergize to drive OSCC progression. | ^4^ |
|  | 1. *Prevotella melaninogenica*  2.Fn  3. *Veillonella parvula* | 1. Induces oral microbiome dysbiosis;  2. Elicits cytokine-driven inflammation. | 16S rRNA | Saliva from 25 OSCC patients and 24 healthy controls | 1. Enriched oncobacteria increase IL-6, IL-8, TNF-α, GM-CSF, and IFN-γ;  2. Cytokine-driven inflammation promotes OSCC proliferation, migration/invasion, and EMT. | ^5^ |
|  | Fn | 1. Associated with improved OS, RFS, and MFS;  2. Correlates with reduced lymph node invasion and metastatic relapse. | 1. 16S rRNA;  2. qPCR;  3.IH | Cohort 1: 122 HNSCC (61 OSCC + 61 non-OSCC)；Cohort 2: 90 OSCC；Merged: 151 OSCC (Cohorts 1+2) | 1. Fn is inversely associated with CD163⁺ M2 macrophages and TLR4 expression;  2. Fn is linked to higher TNFSF9 and IL-1β, suggesting immune microenvironment remodeling toward improved prognosis. | ^6^ |
|  | 1.*Clostridium perfringens*;  2.*Peptostreptococcus*;  3. *Prevotella*;  4.Fn | 1.Induces nuclear translocation of CLDN4;  2. Promotes EMT, stemness, proliferation, and invasion in OSCC cells. | 1. IHC;  2. RT-PCR | 57 OSCC tissue specimens | 1. *C. perfringens*derived enterotoxin (CPE) forms YAP1-ZO-2-CLDN4 complex;  2. The complex suppresses LATS1-mediated YAP1 phosphorylation, enhancing nuclear YAP1 and CCND1/CTGF expression to drive OSCC malignancy. | ^7^ |
|  | *Prevotella intermedia* | 1. Promotes OSCC proliferation, invasion, and tumor growth;  2. Increases pro-inflammatory cytokines and immunosuppressive signaling;  3. Aggravates muscle and perineural invasion. | 1.FISH;  2.16S rRNA;  3.TEM and SEM | 15 OSCC tissues + 10 normal oral tissues | 1. Activates IL-17 signaling, increasing IL-17A, IL-6, TNF-α, and PD-L1;  2. Suppresses GABAergic signaling and tumor suppressors (GABBR2, P63, FASL);  3. Promotes M2 macrophage and Treg infiltration, dampening antitumor immunity. | ^8^ |
|  | *Porphyromonas gingivalis* | 1. Induces NET formation;  2. Promotes OSCC proliferation, migration/invasion, and metastasis;  3. Associates with advanced stage and poor prognosis. | 1. Anaerobic culture;  2. 16S rRNA;  3. IHC;  4. ELISA | 180 OSCC tissues (I–II: 92; III–IV: 88) + 10 normal oral mucosa + PB from 56 OSCC patients | 1. *P. gingivalis* induces NETs formation;  2. NETs remodel the immune microenvironment and enhance OSCC malignancy;  3. DNase I inhibition of NETs attenuates these tumor-promoting effects. | ^9^ |
|  | *Enriched:*  *1. p_Fusobacteria*  *2. c_Fusobacteriia*  *3. o_Fusobacteriales*  *Depleted:*  *1. p_Firmicutes*  *2. p_Actinobacteria* | 1. Potential diagnostic biomarkers for OSCC (AUC 0.686–0.772);  2. Higher enriched taxa and lower *p_Firmicutes* associate with worse prognosis. | NA | 132 TCGA samples (112 OSCC tumors + 20 solid tissue normals) | 1. g_Fn activates TLR2–IL-6/STAT3 signaling, promoting proliferation, migration, and EM;  2. Dysbiosis may drive inflammation-associated oral epithelial malignant transformation. | ^10^ |
| **Glioma**  **Glioma** | *Proteobacteria, Firmicutes, Actinobacteria, Bacteroidetes* | No results posted | 1.16S rRNA;  2.FISH;  3.IHC;  4.TEM;  5.CLEM | 40 GBM samples | NA | ^11^ |
|  | Gram-negative bacteria | NA | 16S rRNA | 3 human glioma tissue specimens | 1. Bacteria localize near nuclear membranes or in intercellular spaces;  2. Exhibit irregular morphologies, possibly cell wall-deficient. | ^12^ |
|  | *Proteobacteria;*  *Actinobacteria;*  *Firmicutes* | 1. Brain metastases show higher diversity; *Paracoccus* enrichment associates with shorter survival;  2. GBM shows lower diversity; bacterial conditioned media modulates TMZ response;  3. Posterior brain metastases show higher richness with enrichment of B1/B7 vitamin biosynthesis pathways. | 1. 16S rDNA;  2. IF;  3. FISH;  4. TEM;  5. Culturomics;  6. Fluorescent D-alanine labeling | 322 brain tumors（156 GBM；166 brain metastases：84 breast-derived，75 lung-derived） | 1. Enriched methylphosphonate degradation may support bacterial phosphorus acquisition in a phosphorus-limited microenvironment;  2. Bacterial conditioned media alters GBM cell sensitivity to temozolomide. | ^13^ |
| **Esophageal cancer** | Enriched:  *Firmicutes*  Depleted: *Proteobacteria* | 1. Distinguishes ESCA subtypes (ESCC vs EAD);  2. Microbial profiles correlate with tumor stage;  3. Potential prognostic biomarkers in ESCA. | NA | 82 esophageal tissues (40 ESCC, 20 EAD, 22 adjacent normal) + TCGA clinical data | 1. Fusobacteria may promote a pro-inflammatory microenvironment that accelerates tumor progression;  2. Dysbiosis characterized by increased Firmicutes and decreased Proteobacteria may contribute to ESCA tumorigenesis;  3. Specific taxa may aid ESCA subtype classification and prognostic stratification. | ^14^ |
|  | *Campylobacter*;  Fn;  *Prevotella* | 1. Associates with an altered immune microenvironment;  2. Linked to reduced megakaryocyte–erythroid progenitors(MEP);  3.Campylobacter/Fusobacterium-positive tumors show increased platelets | 1.16S rRNA;  2.WGS | 3 cohorts: NCI-MD 154 (65 tumors, 89 non-tumors); TCGA RNA-seq 172 (161 tumors, 11 non-tumors); TCGA WGS 123 (62 tumors, 61 non-tumors). | 1. Bacterial LPS engages TLR4 or FcγRIIA signaling pathways to activate platelets, subsequently triggering the release of pro-inflammatory cytokines;  2. MEP-to-platelet differentiation may promote tumor proliferation, invasion, and metastasis;  3. Co-enriched taxa may form biofilms, amplifying pathogenicity and immune dysregulation. | ^15^ |
|  | Fn | 1. Higher Fn abundance associates with advanced pT and clinical stage;  2. Together with high TMB, Fn predicts ESCC metastasis;  3. Potential prognostic biomarker for ESCC progression. | 16S rRNA;  qPCR;  WES;  FISH | 152 ESCC tissues from 111 patients (41 paired tumor/non-tumor; 70 tumor-only); qPCR n=98; WES 20 pairs; FISH n=20. | 1. Fn may modulate EGF-related signaling and reduce apoptosis;  2. Fn-positive ESCC shows distinct gene mutation patterns;  3. TP53 mutations are frequent in Fn-positive tumors, suggesting increased genomic instability. | ^16^ |
|  | Fn | 1. Enriched in PD-1/PD-L1 nonresponders;  2. Reduces anti-PD-L1 efficacy in ESCC models;  3. Suppresses T-cell proliferation and IFN-γ/TNF-α secretion;  4. Promotes immune escape and metastasis. | qPCR;  IF | 12 paired ESCC tumor/normal tissues, 98 ESCC sera, 19 ESCC paraffin sections | 1. Fn-Dps enters the nucleus, binds ATF3, and upregulates PD-L1 transcription in ESCC cells;  2. Fn invades T cells, induces apoptosis, and impairs effector function;  3. PD-L1 upregulation facilitates immune escape from T-cell attack. | ^17^ |
|  | Fn | 1.Induces resistance to 5-FU, cisplatin, and docetaxel in ESCC;  2. Promotes ESCC proliferation;  3. Suppresses chemotherapy-induced apoptosis;  4. Associates with high ATG7 expression in clinical specimens. | 1. qPCR  2. TEM and LSCM  3. Culture of Fn | cohort A 120 resected ESCC; cohort B 30 pretreatment biopsies; cohort C 284 ESCC specimens. | 1. Fn increases ATG7, LC3B-II, and Beclin-1 and induces autophagosome formation;  2. Autophagy mediates Fn-driven chemoresistance and is reversed by ATG7 knockdown or chloroquine;  3. Fn invades and persists intracellularly in ESCC cells. | ^18^ |
|  | *Streptococcus* | 1. Predicts favorable response to neoadjuvant chemoimmunotherapy in ESCC;  2. Correlates with prolonged disease-free survival;  3. Enhances anti-PD-1 treatment efficacy in preclinical models. | 1. 16S rRNA;  2. qRT-PCR;  3. FISH;  4. Bacterial culture;  5. TEM | 1. Clinical samples: 40 ESCC tissues (25 NACI-treated, 15 upfront surgery) + paired adjacent normal tissues + fecal samples; | 1. *Streptococcus* associates with increased CD8⁺ and GrzB⁺ T-cell infiltration and reduced CD4⁺ Tregs;  2. Linked to an activated TIME with more CD8⁺ effector memory T cells;  3. Responder FMT enriches intratumoral Streptococcus and enhances CD8⁺ T-cell recruitment–mediated antitumor immunity. | ^19^ |
|  | *Lactobacillus* | 1. Higher Lactobacillus abundance and Shannon index associate with worse OS in ESCC;  2. Linked to tumor immune microenvironment remodeling;  3. Independent prognostic factor alongside pTNM stage. | 1. 16S rDNA;  2. qPCR;  3. FISH;  4. IHC | 98 ESCC surgical specimens (73 male, 25 female); 25 paired tumor/non-tumorous tissues | 1. Lactobacillus and higher Shannon index associate with increased PD-L1 on epithelial cells and TAMs;  2. They inversely associate with NK-cell infiltration, consistent with an immunosuppressive TME;  3. Lactobacillus may increase intratumoral diversity and jointly shape the TME. | ^20^ |
|  | Fn | 1. Promotes ESCC proliferation and metastasis in vitro and in vivo;  2. Induces METTL3 upregulation across multiple cancer cell types;  3. Associates with worse prognosis in ESCC. | 1. qPCR;  2. IF;  3.IHC | 22 paired ESCC tumor/normal tissues; additional tumor tissues (CRC, BRCA, HCC, n=3 each) | 1. Intracellular Fn increases METTL3 transcription;  2. METTL3 catalyzes m6A modification of c-Myc mRNA (3′UTR);  3.YTHDF1 binds m6A-marked c-Myc mRNA and enhances its stability. | ^21^ |
| **Colorectal Cancer** | Pks+ *Escherichia coli*;  ETBF;  Fn | pks⁺ *E. coli* associates with specific APC mutations in early-onset CRC, particularly in men;  ETBF associates with MMR-deficient tumors and KRAS mutations;  *F. nucleatum* is enriched in right-sided MSI/BRAF-mutant CRC and links to disease progression. | 1. qPCR 2. IHC | 1697 CRC tumor DNA samples (1666 individuals) from 3 cohorts (ACCFR, MCCS, ANGELS); | 1. pks⁺ E. coli produces colibactin, inducing DNA damage (APC mutation);  2. ETBF promotes inflammation-driven tumorigenesis;  3. F. nucleatum exploits MMRd TME, modulates β-catenin/Wnt signaling, and inhibits anti-tumor immunity; | ^22^ |
|  | Colibactin-producing *Escherichia coli*(CoPEC) | 1. CoPEC associates with right-sided CRC relapse, poor survival (stage III–IV), oxaliplatin resistance;  2. Linked to an immunosuppressive TME with reduced CD8⁺ T cells and IFN-γ;  3. Promotes glycerophospholipid accumulation and lipid droplet formation. | 1. 16S rRNA；  2. PCR/qPCR；  3. FISH;  4. Spatial metabolomics；  5. RNAscope | 101 CRC tissues (76 right-sided, 11 left-sided) from 2 cohorts | 1. Colibactin induces ROS and rewires lipid metabolism (Lands cycle; LPCAT2 and DGKG upregulation);  2. Lipid overload supports tumor cell survival and chemoresistance;  3. Reduces tumor immunogenicity with decreased CD8⁺ T-cell infiltration;  4. Triacsin C (acyl-CoA synthetase inhibitor) restores chemosensitivity. | ^23^ |
|  | *Helicobacter pylori* | 1. Promotes intestinal tumorigenesis in Apc-mutant mice (tumor number/size increased);  2. Induces a pro-inflammatory TME with reduced Tregs and enhanced Th17 responses;  3. Impairs the intestinal barrier with goblet cell loss and reduced mucus. | 1. 16S rRNA;  2. Bacterial culture;  3. IHC;  4.qPCR | 154 human colon biopsies | 1. Deregulates immunity: Treg reduction, Treg-to-Foxp3+IL-17A+ cell reprogramming;  2. Activates pro-carcinogenic signaling (STAT3, NF-κB);  3. Induces mucus-degrading microbiota, disrupting intestinal barrier;  4. Synergizes with Apc mutations to accelerate tumor growth. | ^24^ |
|  | *Bacteroides* (predominant), *Parabacteroides* (RSCC), *Bifidobacterium/Roseburia* (LSCC) | 1.Tumor microbiome correlates with ileal microbiome;  2.Surgery/bowel preparation/antibiotics induce dysbiosis (Enterococcus enrichment). | 1. 16S rRNA;  2. DNA extraction | 41 treatment-naive colon cancer patients (24 RSCC, 17 LSCC) | 1. Tumors shape a consistent cancer-associated microbiome across intestinal sites;  2. Grade 3 tumors associate with biofilm-linked taxa (Fn*, Parvimonas*);  3. The ileal microbiome may modulate the tumor microenvironment;  4. Perioperative interventions disrupt gut microbiota homeostasis. | ^25^ |
| **Liver cancer** | *Patescibacteria;*  *Proteobacteria;*  *Bacteroidota* | 1. Higher alpha/beta diversity in peritumor and HCC tissues than normal liver;  2. Specific taxa associate with cirrhosis, HBV status, and tumor volume | 1. 16S rRNA;  2. FISH;  3. qPCR | 155 liver tissues (28 normal, 60 peritumor, 60 HCC); validation: qPCR n=12; culture 12 paired peritumor/HCC; HCC: 49 HBV⁺/21 HBV⁻; cirrhosis n=40 | 1. Bacteria transported to liver via RBCs (intracellular localization);  2. Palmitate activates ROS and glucose uptake, promoting tumor growth;  3. Modulates liver inflammation and immunity via gut-liver axis. | ^26^ |
|  | *Proteobacteria;*  *Actinobacteria;*  *Bacteroidetes* | 1. Microbial profiles differ by tumor vs paratumor, PLC subtype, and prognosis group;  2.Higher Pseudomonas abundance associates with long-term survival. | 1. 16S rRNA;  2. DNA extraction | 28 FFPE tissues from PLC patients | 1. *Pseudomonas* may exert antitumor effects via exopolysaccharides and immunotoxins;  2. Dysbiosis may contribute to worse prognosis;  3. Subtype-specific microbiota may reflect distinct pathogenesis and tumor behavior. | ^27^ |
|  | *Burkholderiales;*  *Pseudomonadales;*  *Xanthomonadales* | 1. *P. fungorum* inhibits tumor proliferation/migration and reduces xenograft tumor growth;  2. *P. fungorum* abundance inversely associates with CA19-9 levels;  3. Microbes localize in malignant and immune cells;  4. Tumors show higher alpha diversity than paracancerous tissues. | 1. 16S rRNA;  2. FISH;  3. Bacterial culture;  4.TEM;  5. qPCR | 94 ICC tissues (42 paired tumor/paracancerous, 3 unpaired tumor, 7 unpaired paracancerous) from 52 patients | 1. *P. fungorum* modulates alanine, aspartate, and glutamate metabolism;  2. Bacterial metabolites inhibit tumor growth;  3. Intracellular bacteria interact directly with tumor/immune cells. | ^28^ |
|  | Enriched taxa: *Enterobacteriaceae;*  Fn*; Neisseria*  Depleted taxa: *Pseudomonas;*  *Dietzia;*  *Faecalibacterium* | 1. Higher alpha/beta diversity in HCC than adjacent tissues;  2. Microbial profiles associate with HBV status and Child–Pugh score;  3. Fatty acid/lipid biosynthesis pathways are enriched. | 1. 16S rRNA;  2. DNA extraction;  3. FISH | 99 tissues (HCC and adjacent nontumor tissues) from HCC patients | 1. Enriched fatty acid/lipid biosynthesis may support tumor proliferation;  2. Pro-tumor taxa (e.g., *Enterobacteriacea*e) may drive inflammation and DNA damage;  3. Gut-to-liver bacterial translocation via portal hypertension may seed intratumoral microbiota. | ^29^ |
|  | 1. HBV-HCC biomarkers: *Cutibacterium, Dolosigranulum, Prevotella, Nocardioides*;  2. Viral-HCC enriched taxa: *Bacteroidales, Parabacteroides, Peptoniphilus, Ruminococcus, Lachnoclostridium;*  3. NBNC-HCC biomarker: *Chryseobacterium* | 1. HBV-HCC shows distinct microbiota with lower α-diversity and separated β-diversity versus HCV-HCC/NBNC-HCC;  2. In HBV-HCC, microbial profiles associate with increased CD8⁺ T cells and M-MDSCs/PMN-MDSCs. | 1. 16S rRNA;  2. DNA extraction | 34 FFPE tissues (5 HBV-HCC, 5 HCV-HCC, 9 NBNC-HCC, 8 Viral-Background, 7 NBNC-Background) | 1. HBV infection may create a niche that favors specific intratumoral microbiota;  2. Microbiota may recruit MDSCs via chemokine signaling (e.g., TLR4–CXCL1–CXCR2), dampening antitumor immunity;  3. HBV–microbiota interplay may jointly shape the TME. | ^30^ |
|  | *Proteobacteria;*  *Actinobacteria;*  *Firmicutes* | 1.Hepatotype independently predicts prognosis (Hepatotype B: longer OS/RFS);  2. *Akkermansia* and *Methylobacterium* associate with favorable prognosis. | 1. 16S rRNA;  2. DNA extraction;  3. FISH | 91 HCC patients (78 paired tumor/adjacent tissues, 13 only tumor tissues; 18 multiple lesions from 8 patients) | 1. The gut–liver axis may enable bacterial translocation to the liverr;  2. Tumors show reduced microbial network complexity, consistent with stress-associated dysbiosis;  3. *Akkermansia* and *Methylobacterium* may shape a favorable TME;  4. *Proteobacteria* and *Actinobacteria* may promote poor prognosis via inflammation. | ^31^ |
| **Pancreatic cancer** | *Acinetobacter;*  *Pseudomonas;*  *Sphingopyxis* | 1. Basal-like subtype shows a distinct microbiome (higher richness, lower Shannon index);  2. Higher abundance of key taxa associates with worse prognosis;  3. Microbial genes are enriched for metabolic, pathogenic, and pro-inflammatory functions;  4. May contribute to aggressive phenotypes via inflammation and host signaling activation. | 1. DNA/RNA extraction;  2.qpcr;  3.FISH;  4.IHC | 62 resectable PDAC tumor tissues | 1. Induces persistent inflammation via LPS and bacterial glycosphingolipids;  2. Activates oncogenic pathways (Kras, MAPK, Wnt/β-Catenin);  3. Impairs bile acid metabolism;  4. Host genetic variation shapes microbial composition. | ^32^ |
|  | *Ruminococcaceae;*  *Staphylococcaceae;*  *Bacillaceae* | 1. Bacterial colonization is more frequent in pancreatic head tumors and Whipple procedure patients;  2. Biliary stenting and gemcitabine/paclitaxel therapy associate with increased Enterobacteriaceae abundance. | 1. 16S rRNA;  2. DNA extraction;  3.qPCR | 54 matched samples (27 tumor, 27 adjacent normal tissues) from 27 PDAC patients | 1. Biliary stenting may facilitate bacterial translocation from the biliary tract to the pancreas;  2. Gemcitabine/paclitaxel therapy may promote *Enterobacteriaceae* expansion under an immunosuppressive state;  3. *Enterobacteriaceae* may contribute to chemoresistance. | ^33^ |
|  | *Porphyromonas gingivalis* | 1. *P. gingivalis* accelerates pancreatic tumor growth and proliferation;  2. Induces neutrophils-dominated pro-inflammatory TME;  3. Reduces CD8^+^ cytotoxic T cells, suppressing antitumor immunity; | 1. 16S rRNA;  2. DNA extraction;  3. FISH;  4.qPCR;  5.IHC | 20 paired tumor/adjacent tissues; 21 saliva samples; 26 FFPE paired samples from pancreatic cancer patients | 1. *P. gingivalis* may translocate from the oral cavity to the pancreas via an oral–gut–pancreas route;  2. increases CXCL1/CXCL2/CXCR2 signaling to recruit TANs;  3. TAN-derived neutrophil elastase is elevated, promoting tumorigenesis;  4. CXCR2 or neutrophil elastase blockade attenuates tumor promotion and restores antitumor immunity. | ^34^ |
|  | 1. Risk-associated taxa: *Streptococcus, Leptotrichia;*  2. Protective taxa: *Veillonella, Neisseria*;  3. Symptom-related taxa: *Porphyromonas*, Fn | 1. Saliva microbiome distinguishes PDAC from healthy individuals;  2. Higher Streptococcus/Leptotrichia correlates with increased PDAC risk;  3. Higher Veillonella/Neisseria correlates with reduced PDAC risk. | 1. 16S rRNA;  2. DNA extraction | 110 saliva samples (41 PDAC patients, 69 healthy controls) | 1. Oral dysbiosis may contribute to PDAC via oral-to-pancreas translocation;  2. Regional dietary patterns (e.g., high-fat/spicy diet) may shape microbial profiles;  3. Symptom-associated taxa may reflect microbiota–host interactions in the TME. | ^35^ |
|  | Fn | 1. Fn associates with larger tumors, lymph node metastasis, and worse prognosis in PDAC;  2. Promotes PDAC cell migration and invasion;  3. Linked to an immunosuppressive TME with increased MDSCs and reduced CD8⁺ T cells;  4. CXCL1–CXCR2 blockade attenuates Fn-driven tumor growth. | 1. qRT-PCR;  2. DNA extraction;  3.IHC | 84 fresh-frozen PDAC tissues; 41 normal pancreatic tissues | 1. Fn stimulates CXCL1/IL-8 secretion from cancer cells;  2. Autocrine signaling: CXCL1 binds CXCR2 on cancer cells, promoting migration/invasion;  3. Paracrine signaling: CXCL1 recruits MDSCs, suppressing CD8+ T cell antitumor activity;  4. Blockade of CXCL1-CXCR2 axis or MDSC depletion reverses tumor progression. | ^36^ |
|  | *Porphyromonas gingivalis* | 1. Promotes pancreatic cancer cell proliferation and in vivo tumor growth;  2. Intracellular survival is enhanced by hypoxia. | 1. RT-qPCR;  2.FISH | NA | 1. P. gingivalis survives intracellularly in PDAC cells, with hypoxia enhancing persistence;  2. Activates Akt signaling pathway and upregulates cyclin D1, heparanase (pro-proliferation factors);  3. Live bacteria are required for pro-tumor effects (heat-killed P. gingivalis has no effect). | ^37^ |
|  | *Klebsiella pneumoniae* | 1. Increased biliary pathogen diversity correlates with reduced PFS;  2. K. pneumoniae associates with larger tumors and abrogates adjuvant gemcitabine benefit; | Microbiome culture | 211 patients with borderline resectable/locally advanced PDAC | 1. *K. pneumoniae* expresses cytidine deaminase, inactivating gemcitabine and driving chemoresistance;  2. Quinolones can inhibit *K. pneumoniae* and show direct antitumor activity;  3. Preoperative biliary stenting may shift microbiota and favor *K. pneumoniae* colonization. | ^38^ |
|  | *Proteobacteria, Firmicutes* | 1. Higher Bacteroides, Lactobacillus, and Peptoniphilus associate with worse prognosis;  2. These taxa correlate with reduced CD4⁺, CD8⁺, and CD45RO⁺ TILs. | 1. qPCR;  2. ISH;  3. 16S rRNA;  4. IHC | 162 resected PDAC FFPE (52 bacteria-positive by qPCR/ISH) + 26 adjacent non-tumor; controls: 15 FFPE contamination blanks. | 1. Hypoxic PDAC TME facilitates colonization of anaerobic bacteria;  2. Prognostic bacteria suppress anti-tumor immunity by reducing effector TIL infiltration. | ^39^ |
|  | Fn | 1. Induces secretion of GM-CSF, CXCL1, IL-8, MIP-3α from PDAC and normal pancreatic epithelial cells;  2. Promotes PDAC cell proliferation and migration via autocrine/paracrine signaling | 1. SEM;  2. Confocal microscopy | NA | 1. Fn invades PDAC cells via Fap2-Gal/GalNAc binding;  2. Secreted cytokines (especially GM-CSF) drive PDAC cell proliferation;  3. Cytokine paracrine/autocrine signaling enhances PDAC cell migration. | ^40^ |
|  | *Clostridium butyricum* (butyrate-producing); | 1. Butyrate-producing bacteria correlate with better PDAC prognosis;  2. C. butyricum/butyrate inhibits PDAC cell proliferation and tumor growth;  3. Enhances PDAC susceptibility to ferroptosis. | 1.qPCR;  2.IHC | 1. Clinical data: TCGA (PAAD) database (long/short-term survivors). | 1. Butyrate downregulates SOD2, inducing intracellular oxidative stress;  2. Promotes fatty acid uptake (upregulates CD36) and inhibits lipolysis (AMPK-pHSL axis), leading to lipid accumulation;  3. Increases lipid peroxidation, enhancing ferroptosis susceptibility;  4. Synergizes with ferroptosis inducer RSL3 to inhibit tumor growth. | ^41^ |
|  | *Bifidobacterium*;  Fn;  *Enterococcus* | 1. PDAC patients have decreased duodenal microbial alpha diversity vs. normal pancreas/pancreatic cysts;  2. Higher bacterial/fungal DNA levels in PDAC duodenal fluid;  3. Duodenal and pancreatic tissue microbiomes show broad similarities (supports retrograde migration). | 1. 16S rRNA and 18S rRNA;  2. DNA extraction | 308 duodenal fluids (74 PDAC, 98 cysts, 134 normal) + 12 paired pancreatic cancer/adjacent non-cancer/duodenal tissues | 1. Intratumoral bacteria in PDAC may originate from duodenal retrograde migration;  2. PPI use induces duodenal dysbiosis (enriches oral bacteria) potentially linked to PDAC risk;  3. Specific bacterial genera correlate with PDAC progression and short survival. | ^42^ |
| **Lung cancer** | *Proteobacteria;*  *Firmicutes;*  *Actinobacteria* | 1. Bacterial metabolic pathways adapt to lung microenvironment (e.g., smoke metabolite degradation);  23. Correlates with patient smoking status. | 1.16S rRNA;  2.qPCR;  3.IHC;  4.FISH;  5.Culturomics | 245 lung tumors + 231 adjacent normals (multi-center); NSCLC subgroup: 100 current smokers + 43 never-smokers; controls: 811 negative controls | 1. Intratumoral bacteria are predominantly intracellular in lung cancer and immune cells;  2. May adopt cell wall–deficient L-form states;  3. Encode pathways for degradation of smoke-derived chemicals, suggesting adaptation to the lung microenvironment. | ^11^ |
|  | *Enriched:* *Modestobacter*  *Depleted:*  *Propionibacterium* | 1. Tumors show lower α-diversity (Shannon/Simpson) than adjacent tissues;  2. Propionibacterium and selected OTUs show diagnostic potential (AUC 78–79.8%). | 16S rRNA | 55 lung cancer patients; 103 tissues (48 tumors + 55 adjacent non-malignant, ≥5 cm from tumor) | 1. Reduced *P. acnes* may weaken Th1/Th17 cytokine–driven antitumor immunity, facilitating immune escape;  2. The TME may drive dysbiosis with strengthened taxa–taxa interactions;  3. Lung microbiota appears mainly airway-derived (nasal) rather than oral/gut-derived. | ^43^ |
|  | *Pasteurella;*  *Pseudomonas;*  *Chryseobacterium* | 1. Pasteurella inhibits lung tumor growth in vivo;  2. Pasteurella correlates with increased CD3+/CD8+ T cell infiltration and decreased M2 macrophages;  3. Coriobacteriaceae correlates with tumor burden and reduced CD8+ T cells. | 16S rRNA | Bronchoalveolar fluid (BAL) from 4 NSCLC patients (stage I) and 4 non-cancer controls | 1. Pasteurella triggers cytotoxic immune response by recruiting CD8+ TILs;  2. Reduces immunosuppressive M2 macrophage infiltration;  3. BAL from NSCLC patients reshapes lung microbiota, favoring Pasteurella colonization to inhibit tumor growth. | ^44^ |
|  | *1.* LUAD-associated:*Escherichia coli;*  *2.* LUSC-associated: *Pseudomonas* | 1. Age- and gender-specific microbial dysbiosis in LUAD/LUSC;  3. E. coli str. K-12 (LUAD) correlates with poor survival (oncogenic) and genomic alterations;  4. P. putida KT2440 (LUSC) correlates with reduced immune cell infiltration. | NA | TCGA RNA-seq data (497 LUAD + 433 LUSC tumors; 59 LUAD + 49 LUSC adjacent normal tissues); | 1. Microbes correlate with immune cell populations;  2. Modulate immune/cancer pathways (e.g., CD8 T cell/macrophage-related pathways);  3. E. coli str. K-12 associates with deletions/mutations. | ^45^ |
|  | *Escherichia-Shigella;*  *Faecalibacterium;*  *Pseudomonas* | Tumor tissues selectively harbor enteric and potentially pathogenic bacteria | 16S rRNA | 29 NSCLC patients (18 LUAD + 11 SqCC) with paired tumor and adjacent healthy tissues (≥5 cm from tumor) | 1. Tumor microenvironment may support colonization/survival of enteric/pathogenic bacteria;  2. Inter-individual microbiota variation masks broad cancer subtype/tissue-type associated patterns;  3. Phascolarctobacterium enrichment in SqCC tumor suggests subtype-specific microbial adaptation. | ^46^ |
|  | *Proteus* and *Bacteroides* | Proteus and Bacteroides correlate with LN metastasis of LUSC | NA | TCGA-LUSC cohort (461 patients) divided into LN+ (165, T≥1N>0M0) and LN− (296, T≥1N0M0) groups | NA | ^47^ |
|  | *Porphyromonas gingivalis (P. gingivalis)* | 1.Correlates with smoking, alcohol consumption, lymph node metastasis, and advanced clinical stages (III/IV);  2. Predicts poor prognosis. | IHC | 319 lung cancer patients (100 small cell lung cancer + 119 adenocarcinoma + 100 squamous cell carcinoma) with paired tumor and adjacent non-malignant tissues (≥5 cm from tumor) | 1. The TME may facilitate *P. gingivalis* colonization via immune evasion;  2. *P. gingivalis* induces immunosuppression (e.g., T-cell dysfunction and checkpoint upregulation), promoting invasion, proliferation, and metastasis;  3. Smoking and alcohol use may impair immunity and facilitate *P. gingivalis* infection. | ^48^ |
| **Breast cancer** | *Proteobacteria;*  *Firmicutes;*  *Actinobacteria* | Correlates with tumor subtypes and predicted metabolic functions. | 1.16S rRNA;  2.FISH;  3.IHC;  4.TEM;  5.CLEM | 355 breast tumor samples + 256 breast NAT samples (from 3 medical centers) | 1. Intratumoral bacteria are intracellular in cancer and immune cells (macrophages, CD45⁺ cells);  2. ER⁺ tumors are enriched for bacterial genes involved in mycothiol biosynthesis (ROS detoxification) and arsenate detoxification. | ^11^ |
|  | Tumor-enriched: *Pseudomonas; Proteus; Porphyromonas*  Tumor-depleted: *Propionibacterium;*  *Staphylococcus;*  *Streptococcus* | 1.Microbial profiles associate with stage, grade, subtype, and lymphovascular invasion;  2.Propionibacterium/Streptococcus correlate with T-cell activation signatures, whereas *Cloacibacterium/Methylibium* associate with oncogenic immune features. | 1. 16S rRNA;  2. qpcr;  3. IHC | 308 fresh-frozen breast tissues (221 breast cancer tumors + 18 high-risk + 69 healthy controls); paired tumor-adjacent normal tissues available for some patients | 1. Microbiota–immune crosstalk modulates the TME (e.g., T-cell activation and TLR signaling);  2. Loss of putative tumor-suppressive taxa (*Propionibacterium*, *Streptococcus*) may weaken antitumor immunity;  3. Enriched taxa (*Cloacibacterium*, *Lactobacillus*) associate with pro-oncogenic immune features;  4. Subtype-specific microbiota may shape subtype-specific immune programs. | ^49^ |
|  | *Proteobacteria;*  *Firmicutes;*  *Actinobacteria* | Synergize with fungi and immune cells to form survival-associated “mycotypes,” and combined bacterial–fungal biomarkers improve diagnostic performance. | 1. WGS;  2. 16S rRNA | 17,401 samples (35 cancers; 4 cohorts): TCGA 15,512 (tumor/NAT/blood); WIS 1,183 (tumor/NAT/normal) + 295 controls; Hopkins 537 plasma; UCSD 169 plasma + 52 controls. | 1. The TME may permit bacterial–fungal co-colonization;  2. Bacteria-fungi-immune crosstalk may shape immune subtypes (e.g., inflammatory, macrophage-enriched) and tumor progression. | ^50^ |
|  | *S. xylosus;*  *L. animalis;*  *S. cuniculi.* | 1. Promotes lung metastasis without affecting primary tumor growthh;  2. Enhances CTC survival under fluid shear stress;  3. Enriched in CTC clusters and metastatic foci;  4. Induces metastatic colonization in low-metastatic models. | 1. 16S rRNA;  2. qPCR;  3. Bacteria culture and isolation;  4. FISH;  5. TEM | Human samples: 11 breast tumor tissues, 6 adjacent normal breast tissues, 4 lymph node metastases. | 1. Promotes lung metastasis without affecting primary tumor growth;  2. Enhances CTC survival under fluid shear stress;  2. Reduces stress fiber formation to relieve mechanical contractile forces;  3. Enriched in CTC clusters and metastatic foci;  4. Induces metastatic colonization in low-metastatic models. | ^51^ |
|  | *Ralstonia;*  *Staphylococcus* | 1. Microbiota clusters differ between BNH and WNH in tumors and NATs;  2. TPBC tumors show higher Fn and *Streptococcus*. | 16S rRNA | 66 frozen breast tissues (tumor + matched NAT) from New England cohort | NA | ^52^ |
|  | ETBF | 1.Induces mammary epithelial hyperplasia (local ductal/gut colonization);  2. Enhances breast cancer migration, invasion, and stemness;  3. Promotes multifocal tumors and lung/liver metastasis. | 1. 16S rRNA;  2. Bacteria culture and purification;  3. qPCR;  4.IHC;  5.IF | NA | 1. BFT activates β-Catenin pathway (nuclear accumulation, downregulates phospho-β-Catenin);  2. Activates Notch1 pathway (increases NICD nuclear localization);  3. β-Catenin-Notch1 crosstalk mediates oncogenic effects;  4. Induces cytoskeletal remodeling and E-cadherin cleavage. | ^53^ |
|  | *Blautia;*  *Ruminococcus;*  *Faecalibacterium* | Gut microbial cutC gene produces TMA, which converts to TMAO, which enhances anti-PD-1 immunotherapy efficacy in TNBC. | 1. 16S rRNA;  2. FISH;  3. qPCR | 360 TNBC tissues (FUSCCTNBC cohort), 12 plasma samples from immunotherapy-treated TNBC patients | 1. Clostridiales-derived TMA is oxidized to TMAO, which activates PERK-mediated ER stress;  2. Induces caspase 3/GSDME-dependent tumor cell pyroptosis;  3. Releases IL-1β/IL-18 to enhance CD8⁺ T cell-mediated antitumor immunity. | ^54^ |
|  | *Bacteroidetes;*  *Firmicutes;*  *Proteobacteria* | 1. Tumors show lower alpha diversity (Simpson index) than normal tissue;  2. Tumor-associated taxa are enriched for pro-inflammatory and carcinogenesis-related KEGG pathways. | 16S rRNA | 46 breast tissues (10 archived tumor samples, 36 normal tissues from 10 reduction mammoplasty patients) | 1. Tumor-associated taxa may promote carcinogenesis via inflammation (e.g., IL-8, CRP);  2. Tumor microbiota is enriched for base excision repair, Th17 differentiation, and IL-17/PI3K–Akt pathways;  3. Bilateral microbiota differences may contribute to unilateral breast cancer. | ^55^ |
|  | Fn | 1. Colonizes breast tumors via Fap2-Gal-GalNAc interaction;  2. Accelerates primary tumor growth and lung metastasis;  3. Suppresses tumor-infiltrating CD4⁺/CD8⁺ T cells;  4. Induces MMP-9 secretion to promote tumor progression. | 1. 16S rRNA;  2. qPCR and bacterial culture;  3. Multiphoton microscopy | 50 human breast tumor FFPE samples, 21 colorectal tumor controls | 1. Fap2 lectin binds to tumor-displayed Gal-GalNAc for colonization;  2. Reduces T cell infiltration to dampen antitumor immunity;  3. Upregulates MMP-9 to enhance tumor invasion/metastasis;  4. Effects are reversible with metronidazole treatment. | ^56^ |
|  | ETBF | 1. Secretes BFT-1 to enhance breast cancer stem cell (BCSC) stemness (ALDH⁺ population);  2. Induces taxane-based chemotherapy resistance;  3. Correlates with poor response to neoadjuvant chemotherapy in patients. | 1. 16S rRNA;  2. FISH;  3. qPCR;  4. Bacterial culture | Tumor/para-tumor tissues from 9 breast cancer patients (4 complete responders, 5 non-responders to TNC); 68 pre-TNC tumor biopsies | 1. BFT-1 binds NOD1 and stabilizes NOD1 protein;  2. NOD1 engages GAK to phosphorylate NUMB, promoting lysosomal degradation;  3. NOTCH1–HEY1 signaling is activated to enhance BCSC self-renewal. | ^57^ |
|  | Protective: *Acidibacillus;*  *Succinimonas*   Risk : *Lachnoclostridium;*  *Pseudogulbenkiania* | 1. Stratifies two immune subtypes (immune-enriched vs immune-deficient) with distinct prognosis;  2. Correlates with CD8⁺ T cells, NK cells, and macrophages infiltration;  3. Predicts response to PD-1/CTLA-4 blockade and to tamoxifen/docetaxel;  4. Associates with immune pathways and oncogenic signaling programs. | 16S rRNA | 1,018 TCGA-BRCA primary breast cancer samples (with OS >1 month) | 1. Immune-enriched taxa (*Lachnoclostridium*) associate with antigen presentation and NK cytotoxicity pathways;  2. Immune-deficient taxa (*Succinimonas*) associate with TGF-β and Wnt signaling;  3. Microbiota links to TNFRSF4, CD27, and CTLA-4 expression, modulating T-cell infiltration;  4. The prognostic signature may relate to chemosensitivity via drug-response signaling programs. | ^58^ |
| **Ovarian Cancer** | *Proteobacteria*;  *Firmicutes;*  *Actinobacteria* | NA | 1.16S rRNA;  2.FISH;  3.IHC;  4.TEM;  5.Correlative Light and Electron Microscopy | 58 Ovarian tumor and 28 normal samples | Adapts to ovarian tumor microenvironment via specialized metabolic functions; | ^11^ |
|  | Enriched:  *Aquificae;*  *Planctomycetes;*  Decreased: *Crenarchaeota* | 1.Enriched in potentially pathogenic and oxidative stress-tolerant phenotypes;  2.Specific taxa correlate with ovarian cancer diagnosis and stage. | 1.16S rRNA;  2.IHC | 16 ovarian tissue samples (6 cancerous: serous ovarian cancer, 10 noncancerous: uterine myoma/adenomyosis-associated) | 1. Bacteria may originate from female reproductive tract continuum (vagina to ovaries) or blood/abdominal cavity;  2. Enriched pathogenic/oxidative stress-tolerant phenotypes adapt to tumor microenvironment; | ^59^ |
|  | *Pseudomonas* | 1. Distinguishes two immune subtypes (immune-enriched/deficient) with distinct prognoses;  2. Interacts with tumor immunity (M1 macrophages, CD8⁺ T cells). | Metagenomic profiling | 373 TCGA-OV patients (serous ovarian cancer) with RNA-seq, clinical, and survival data; | 1. Protective taxa (Achromobacter deleyi) positively correlate with M1 macrophages (antitumor immunity);  2. Risk taxa (Acinetobacter seifertii) inhibit macrophage migration to impair immune infiltration. | ^60^ |
|  | Cancer-enriched genera: *Bosea; Mesorhizobium;*  *Mycobacterium*  Cancer-depleted genera: *Acidovorax; Acinetobacter; Aeromonas* | NA | 16S rRNA | 25 tissue biopsies from 10 postmenopausal women (2 with high-grade serous ovarian cancer, 8 with benign disease) | 1. Bacterial dysbiosis correlates with epithelial ovarian cancer;  2. Proteobacteria dominance suggests adaptation to URT microenvironment. | ^61^ |
|  | *Acinetobacter* | 1. Acinetobacter serves as a core microbiome biomarker for distinguishing ovarian cancer from benign ovarian tumors;  2. Serum microbe-derived EVs enable non-invasive liquid biopsy for ovarian cancer diagnosis. | 16S rDNA | 242 serum samples (166 ovarian cancer, 76 benign ovarian tumors) | 1. Acinetobacter may promote ovarian cancer via LPS-TLR4 signaling pathway;  2. Microbe-derived EVs mediate host-microbe interactions, contributing to tumorigenesis and immune modulation. | ^62^ |
| **Cervical cancer** | Fn | 1. Enriched in recurrent vs primary cervical cancer and in stage III/IV vs early-stage tumors;  2. Associates with poor differentiation and shorter OS/PFS;  3. Independent prognostic factor for PFS (HR 4.8, P=0.024);  4. Enhances CSC traits (sphere formation, invasion) and upregulates CSC/metastasis genes. | qPCR | 112 cervical SCC (stage IB1/IB2–IIA1/IIA2); 23 paired primary/recurrent tissues; 20 stage III/IV biopsies; controls: 23 matched adjacent non-tumor tissues. | 1. Activates CSC-related pathways (e.g., Wnt/β-catenin, IGF-1R) with increased NANOG, OCT4, and SOX2;  2. Upregulates CXCR4, Ep-CAM, Slug, and Snail1, enhancing invasion;  3. May promote progression/recurrence by reinforcing CSC stemness and remodeling the tumor microenvironment. | ^63^ |
|  | Decreased: *Shuttleworthia;*  *Prevotella;*  *Lactobacillus*  Increased: *Streptococcus;*  *Faecalibacterium; Bifidobacteriaceae* | Microbiota dysbiosis correlates with cervical cancer progression | 16S rRNA | 122 cervical scraping samples | 1. Microbiota dysbiosis disrupts vaginal microecological balance, promoting HPV adhesion/invasion;  2. Inhibited pathways impair immune function and epithelial integrity;  3. Probiotic depletion (e.g., Lactobacillus) reduces vaginal acidification and pathogen resistance. | ^64^ |
| **Bone Cancer** | *Proteobacteria;*  *Firmicutes;*  *Actinobacteria* | 1. Enriched for hydroxyproline-degrading bacteria in bone (collagen-rich niche);  2. Metabolic functions may support microenvironmental adaptation. | 1. 16S rDNA;  2. qPCR;  3. IHC/FISH;  4. CLEM/TEM;  5. Culturomics | 39 bone tumor tissues | 1. Bacteria may adapt to the bone niche by utilizing hydroxyproline from collagen turnover;  2. Intracellular, cell wall–deficient (L-form) states may facilitate immune evasion;  3. Metabolic programs may support survival and adaptation within the tumor niche. | ^11^ |
|  | Enriched: *Alloprevotella;*  *Prevotella;*  *Selenomonas*  Depleted:  *Rothia;*  *Halomonas;*  *Rhodococcus* | 1.Alloprevotella positively correlates with alkaline phosphatase;  2. Altered microbial functions involve glycan degradation, ansamycin biosynthesis, and folate metabolism. | 16S rRNA | 135 oral swabs (45 osteosarcoma patients, 90 healthy controls) | 1. Microbial dysbiosis may modulate bone metabolism;  2. Enriched opportunistic pathogens (e.g., Campylobacter) may promote tumorigenesis;  3. Altered metabolic pathways (glycan degradation, folate metabolism) support tumor niche adaptation. | ^65^ |

Abbreviations: OSCC, oral squamous cell carcinoma; Fn, Fusobacterium nucleatum; EMT, epithelial-mesenchymal transition; TAMs, tumor-associated macrophages; qPCR, quantitative real-time polymerase chain reaction; FISH, fluorescence in situ hybridization; HNSCC, head and neck squamous cell carcinoma; OS, overall survival; RFS, recurrence-free survival; MFS, metastasis-free survival; IHC, immunohistochemistry; RT-PCR, reverse transcription polymerase chain reaction; CPE, Clostridium perfringens enterotoxin; NET, neutrophil extracellular trap; ELISA, enzyme-linked immunosorbent assay; PB, peripheral blood; AUC, area under the curve; TCGA, The Cancer Genome Atlas; GBM, glioblastoma multiforme; TEM, transmission electron microscopy; CLEM, correlative light and electron microscopy; 16S rDNA, 16S ribosomal deoxyribonucleic acid; IF, immunofluorescence; Culturomics, culturomics; TMZ, temozolomide; ESCA, esophageal cancer; ESCC, esophageal squamous cell carcinoma; EAD, esophageal adenocarcinoma; MEP, megakaryocyte–erythroid progenitors; WGS, whole-genome sequencing; LPS, lipopolysaccharide; TLR4, toll-like receptor 4; FcγRIIA, fragment crystallizable gamma receptor IIA; TMB, tumor mutational burden; WES, whole-exome sequencing; IFN-γ, interferon-gamma; TNF-α, tumor necrosis factor-alpha; ATF3, activating transcription factor 3; 5-FU, 5-fluorouracil; LSCM, laser scanning confocal microscopy; ATG7, autophagy-related 7; LC3B-II, microtubule-associated protein 1 light chain 3B-II; NACI, neoadjuvant chemoimmunotherapy; GrzB, granzyme B; Tregs, regulatory T cells; TIME, tumor immune microenvironment; FMT, fecal microbiota transplantation; NK cell, natural killer cell; METTL3, methyltransferase-like 3; m6A, N6-methyladenosine; YTHDF1, YTH N6-methyladenosine RNA binding protein 1; CRC, colorectal cancer; BRCA, breast cancer; HCC, hepatocellular carcinoma; APC, adenomatous polyposis coli; ETBF, enterotoxigenic Bacteroides fragilis; MSI, microsatellite instability; CoPEC, colibactin-producing Escherichia coli; MMRd, mismatch repair-deficient; ROS, reactive oxygen species; LPCAT2, lysophosphatidylcholine acyltransferase 2; DGKG, diacylglycerol kinase gamma; STAT3, signal transducer and activator of transcription 3; NF-κB, nuclear factor-kappa B; RSCC, right-sided colorectal cancer; LSCC, left-sided colorectal cancer; PLC, primary liver cancer; ICC, intrahepatic cholangiocarcinoma; CA19-9, carbohydrate antigen 19-9; HBV, hepatitis B virus; HCV, hepatitis C virus; NBNC-HCC, non-B non-C hepatocellular carcinoma; MDSCs, myeloid-derived suppressor cells; CXCL1, C-X-C motif chemokine ligand 1; CXCR2, C-X-C motif chemokine receptor 2; PDAC, pancreatic ductal adenocarcinoma; MAPK, mitogen-activated protein kinase; TANs, tumor-associated neutrophils; GM-CSF, granulocyte-macrophage colony-stimulating factor; IL-8, interleukin-8; MIP-3α, macrophage inflammatory protein 3 alpha; PFS, progression-free survival; SOD2, superoxide dismutase 2; CD36, cluster of differentiation 36; AMPK, adenosine 5'-monophosphate-activated protein kinase; pHSL, phosphorylated hormone-sensitive lipase; RSL3, RAS-selective lethal 3; PAAD, pancreatic adenocarcinoma; PPI, proton pump inhibitor; SEM, scanning electron microscopy; LUAD, lung adenocarcinoma; LUSC, lung squamous cell carcinoma; NSCLC, non-small cell lung cancer; OTUs, operational taxonomic units; BAL, bronchoalveolar fluid; TILs, tumor-infiltrating lymphocytes; SqCC, squamous cell carcinoma; CTC, circulating tumor cell; LN, lymph node; ER⁺, estrogen receptor positive; TNBC, triple-negative breast cancer; FUSCCTNBC, Fudan University Shanghai Cancer Center triple-negative breast cancer; TMA, trimethylamine; TMAO, trimethylamine N-oxide; PERK, protein kinase R-like endoplasmic reticulum kinase; ER, endoplasmic reticulum; GSDME, gasdermin E; MMP-9, matrix metalloproteinase-9; BCSC, breast cancer stem cell; ALDH⁺, aldehyde dehydrogenase positive; TNC, taxane-based neoadjuvant chemotherapy; NOD1, nucleotide-binding oligomerization domain 1; GAK, cyclin G-associated kinase; NUMB, numb endocytic adaptor protein; HEY1, hes related family bHLH transcription factor 1; CTLA-4, cytotoxic T-lymphocyte-associated protein 4; IGF-1R, insulin-like growth factor 1 receptor; NANOG, Nanog homeobox; OCT4, octamer-binding transcription factor 4; SOX2, SRY-box transcription factor 2; Ep-CAM, epithelial cell adhesion molecule; HR, hazard ratio; HPV, human papillomavirus; RNAscope, RNA in situ hybridization; FFPE, formalin-fixed paraffin-embedded; KEGG, Kyoto Encyclopedia of Genes and Genomes; CRP, C-reactive protein; PI3K–Akt, phosphatidylinositol 3-kinase–protein kinase B; URT, upper reproductive tract; EVs, extracellular vesicles; 16S rRNA, 16S ribosomal ribonucleic acid

1. Su SC, Chang LC, Huang HD, et al. Oral microbial dysbiosis and its performance in predicting oral cancer. *Carcinogenesis*. Feb 11 2021;42(1):127-135. doi:10.1093/carcin/bgaa062

2. Geng F, Zhang Y, Lu Z, Zhang S, Pan Y. Fusobacterium nucleatum Caused DNA Damage and Promoted Cell Proliferation by the Ku70/p53 Pathway in Oral Cancer Cells. *DNA and cell biology*. Jan 2020;39(1):144-151. doi:10.1089/dna.2019.5064

3. Sun J, Tang Q, Yu S, et al. F. nucleatum facilitates oral squamous cell carcinoma progression via GLUT1-driven lactate production. *EBioMedicine*. Feb 2023;88:104444. doi:10.1016/j.ebiom.2023.104444

4. Sarkar P, Malik S, Laha S, et al. Dysbiosis of Oral Microbiota During Oral Squamous Cell Carcinoma Development. *Frontiers in oncology*. 2021;11:614448. doi:10.3389/fonc.2021.614448

5. Rai AK, Panda M, Das AK, et al. Dysbiosis of salivary microbiome and cytokines influence oral squamous cell carcinoma through inflammation. *Archives of microbiology*. Jan 2021;203(1):137-152. doi:10.1007/s00203-020-02011-w

6. Neuzillet C, Marchais M, Vacher S, et al. Prognostic value of intratumoral Fusobacterium nucleatum and association with immune-related gene expression in oral squamous cell carcinoma patients. *Sci Rep*. Apr 12 2021;11(1):7870. doi:10.1038/s41598-021-86816-9

7. Nakashima C, Yamamoto K, Kishi S, et al. Clostridium perfringens enterotoxin induces claudin-4 to activate YAP in oral squamous cell carcinomas. *Oncotarget*. Jan 28 2020;11(4):309-321. doi:10.18632/oncotarget.27424

8. Zhou Y, Qin Y, Ma J, et al. Heat-killed Prevotella intermedia promotes the progression of oral squamous cell carcinoma by inhibiting the expression of tumor suppressors and affecting the tumor microenvironment. *Experimental hematology & oncology*. Mar 21 2024;13(1):33. doi:10.1186/s40164-024-00500-y

9. Guo ZC, Jing SL, Jia XY, et al. Porphyromonas gingivalis promotes the progression of oral squamous cell carcinoma by stimulating the release of neutrophil extracellular traps in the tumor immune microenvironment. *Inflammation research : official journal of the European Histamine Research Society [et al]*. May 2024;73(5):693-705. doi:10.1007/s00011-023-01822-z

10. Li Z, Fu R, Wen X, Wang Q, Huang X, Zhang L. The significant clinical correlation of the intratumor oral microbiome in oral squamous cell carcinoma based on tissue-derived sequencing. *Frontiers in physiology*. 2022;13:1089539. doi:10.3389/fphys.2022.1089539

11. Nejman D, Livyatan I, Fuks G, et al. The human tumor microbiome is composed of tumor type-specific intracellular bacteria. *Science*. 2020;368(6494):973-980. doi:10.1126/science.aay9189

12. Zhao J, He D, Lai HM, et al. Comprehensive histological imaging of native microbiota in human glioma. *Journal of biophotonics*. Apr 2022;15(4):e202100351. doi:10.1002/jbio.202100351

13. Yuan L, Pan L, Wang Y, et al. Characterization of the landscape of the intratumoral microbiota reveals that Streptococcus anginosus increases the risk of gastric cancer initiation and progression. *Cell discovery*. Nov 26 2024;10(1):117. doi:10.1038/s41421-024-00746-0

14. Wang Y, Guo H, Gao X, Wang J. The Intratumor Microbiota Signatures Associate With Subtype, Tumor Stage, and Survival Status of Esophageal Carcinoma. *Frontiers in oncology*. 2021;11:754788. doi:10.3389/fonc.2021.754788

15. Greathouse KL, Stone JK, Vargas AJ, et al. Co-enrichment of cancer-associated bacterial taxa is correlated with immune cell infiltrates in esophageal tumor tissue. *Sci Rep*. Jan 31 2024;14(1):2574. doi:10.1038/s41598-023-48862-3

16. Li Z, Shi C, Zheng J, et al. Fusobacterium nucleatum predicts a high risk of metastasis for esophageal squamous cell carcinoma. *BMC microbiology*. Oct 30 2021;21(1):301. doi:10.1186/s12866-021-02352-6

17. Li Y, Xing S, Chen F, et al. Intracellular Fusobacterium nucleatum infection attenuates antitumor immunity in esophageal squamous cell carcinoma. *Nat Commun*. Sep 18 2023;14(1):5788. doi:10.1038/s41467-023-40987-3

18. Liu Y, Baba Y, Ishimoto T, et al. Fusobacterium nucleatum confers chemoresistance by modulating autophagy in oesophageal squamous cell carcinoma. *Br J Cancer*. Mar 2021;124(5):963-974. doi:10.1038/s41416-020-01198-5

19. Wu H, Leng X, Liu Q, et al. Intratumoral Microbiota Composition Regulates Chemoimmunotherapy Response in Esophageal Squamous Cell Carcinoma. *Cancer Res*. Sep 15 2023;83(18):3131-3144. doi:10.1158/0008-5472.Can-22-2593

20. Zhang S, Zhang S, Ma X, et al. Intratumoral microbiome impacts immune infiltrates in tumor microenvironment and predicts prognosis in esophageal squamous cell carcinoma patients. *Front Cell Infect Microbiol*. 2023;13:1165790. doi:10.3389/fcimb.2023.1165790

21. Guo S, Chen F, Li L, et al. Intracellular Fusobacterium nucleatum infection increases METTL3-mediated m6A methylation to promote the metastasis of esophageal squamous cell carcinoma. *J Adv Res*. Jul 2024;61:165-178. doi:10.1016/j.jare.2023.08.014

22. Joo JE, Chu YL, Georgeson P, et al. Intratumoral presence of the genotoxic gut bacteria pks(+) E. coli, Enterotoxigenic Bacteroides fragilis, and Fusobacterium nucleatum and their association with clinicopathological and molecular features of colorectal cancer. *Br J Cancer*. Mar 2024;130(5):728-740. doi:10.1038/s41416-023-02554-x

23. de Oliveira Alves N, Dalmasso G, Nikitina D, et al. The colibactin-producing Escherichia coli alters the tumor microenvironment to immunosuppressive lipid overload facilitating colorectal cancer progression and chemoresistance. *Gut Microbes*. Jan-Dec 2024;16(1):2320291. doi:10.1080/19490976.2024.2320291

24. Ralser A, Dietl A, Jarosch S, et al. Helicobacter pylori promotes colorectal carcinogenesis by deregulating intestinal immunity and inducing a mucus-degrading microbiota signature. *Gut*. Jul 2023;72(7):1258-1270. doi:10.1136/gutjnl-2022-328075

25. Kneis B, Wirtz S, Weber K, et al. Colon Cancer Microbiome Landscaping: Differences in Right- and Left-Sided Colon Cancer and a Tumor Microbiome-Ileal Microbiome Association. *Int J Mol Sci*. Feb 7 2023;24(4)doi:10.3390/ijms24043265

26. Huang JH, Wang J, Chai XQ, et al. The Intratumoral Bacterial Metataxonomic Signature of Hepatocellular Carcinoma. *Microbiology spectrum*. Oct 26 2022;10(5):e0098322. doi:10.1128/spectrum.00983-22

27. Qu D, Wang Y, Xia Q, Chang J, Jiang X, Zhang H. Intratumoral Microbiome of Human Primary Liver Cancer. *Hepatol Commun*. Jul 2022;6(7):1741-1752. doi:10.1002/hep4.1908

28. Chai X, Wang J, Li H, et al. Intratumor microbiome features reveal antitumor potentials of intrahepatic cholangiocarcinoma. *Gut Microbes*. Jan-Dec 2023;15(1):2156255. doi:10.1080/19490976.2022.2156255

29. He Y, Zhang Q, Yu X, Zhang S, Guo W. Overview of microbial profiles in human hepatocellular carcinoma and adjacent nontumor tissues. *Journal of translational medicine*. Feb 2 2023;21(1):68. doi:10.1186/s12967-023-03938-6

30. Liu Y, Kim ES, Guo H. Hepatitis B virus-related hepatocellular carcinoma exhibits distinct intratumoral microbiota and immune microenvironment signatures. *Journal of medical virology*. Feb 2024;96(2):e29485. doi:10.1002/jmv.29485

31. Sun L, Ke X, Guan A, et al. Intratumoural microbiome can predict the prognosis of hepatocellular carcinoma after surgery. *Clinical and translational medicine*. Jul 2023;13(7):e1331. doi:10.1002/ctm2.1331

32. Guo W, Zhang Y, Guo S, et al. Tumor microbiome contributes to an aggressive phenotype in the basal-like subtype of pancreatic cancer. *Communications biology*. Aug 31 2021;4(1):1019. doi:10.1038/s42003-021-02557-5

33. Nalluri H, Jensen E, Staley C. Role of biliary stent and neoadjuvant chemotherapy in the pancreatic tumor microbiome. *BMC microbiology*. Oct 16 2021;21(1):280. doi:10.1186/s12866-021-02339-3

34. Tan Q, Ma X, Yang B, et al. Periodontitis pathogen Porphyromonas gingivalis promotes pancreatic tumorigenesis via neutrophil elastase from tumor-associated neutrophils. *Gut Microbes*. Jan-Dec 2022;14(1):2073785. doi:10.1080/19490976.2022.2073785

35. Wei AL, Li M, Li GQ, et al. Oral microbiome and pancreatic cancer. *World J Gastroenterol*. Dec 28 2020;26(48):7679-7692. doi:10.3748/wjg.v26.i48.7679

36. Hayashi M, Ikenaga N, Nakata K, et al. Intratumor Fusobacterium nucleatum promotes the progression of pancreatic cancer via the CXCL1-CXCR2 axis. *Cancer science*. Sep 2023;114(9):3666-3678. doi:10.1111/cas.15901

37. Gnanasekaran J, Binder Gallimidi A, Saba E, et al. Intracellular Porphyromonas gingivalis Promotes the Tumorigenic Behavior of Pancreatic Carcinoma Cells. *Cancers*. Aug 18 2020;12(8)doi:10.3390/cancers12082331

38. Weniger M, Hank T, Qadan M, et al. Influence of Klebsiella pneumoniae and quinolone treatment on prognosis in patients with pancreatic cancer. *The British journal of surgery*. Jun 22 2021;108(6):709-716. doi:10.1002/bjs.12003

39. Abe S, Masuda A, Matsumoto T, et al. Impact of intratumoral microbiome on tumor immunity and prognosis in human pancreatic ductal adenocarcinoma. *Journal of gastroenterology*. Mar 2024;59(3):250-262. doi:10.1007/s00535-023-02069-5

40. Udayasuryan B, Ahmad RN, Nguyen TTD, et al. Fusobacterium nucleatum induces proliferation and migration in pancreatic cancer cells through host autocrine and paracrine signaling. *Science signaling*. Oct 18 2022;15(756):eabn4948. doi:10.1126/scisignal.abn4948

41. Yang X, Zhang Z, Shen X, et al. Clostridium butyricum and its metabolite butyrate promote ferroptosis susceptibility in pancreatic ductal adenocarcinoma. *Cellular oncology (Dordrecht, Netherlands)*. Dec 2023;46(6):1645-1658. doi:10.1007/s13402-023-00831-8

42. Kohi S, Macgregor-Das A, Dbouk M, et al. Alterations in the Duodenal Fluid Microbiome of Patients With Pancreatic Cancer. *Clinical gastroenterology and hepatology : the official clinical practice journal of the American Gastroenterological Association*. Feb 2022;20(2):e196-e227. doi:10.1016/j.cgh.2020.11.006

43. Mao Q, Ma W, Wang Z, et al. Differential flora in the microenvironment of lung tumor and paired adjacent normal tissues. *Carcinogenesis*. Aug 12 2020;41(8):1094-1103. doi:10.1093/carcin/bgaa044

44. Zheng L, Xu J, Sai B, et al. Microbiome Related Cytotoxically Active CD8+ TIL Are Inversely Associated With Lung Cancer Development. *Frontiers in oncology*. 2020;10:531131. doi:10.3389/fonc.2020.531131

45. Wong LM, Shende N, Li WT, et al. Comparative Analysis of Age- and Gender-Associated Microbiome in Lung Adenocarcinoma and Lung Squamous Cell Carcinoma. *Cancers*. Jun 2 2020;12(6)doi:10.3390/cancers12061447

46. Dumont-Leblond N, Veillette M, Racine C, Joubert P, Duchaine C. Non-small cell lung cancer microbiota characterization: Prevalence of enteric and potentially pathogenic bacteria in cancer tissues. *PLoS One*. 2021;16(4):e0249832. doi:10.1371/journal.pone.0249832

47. Liang P, Deng H, Zhao Y, et al. Microbiota modulate lung squamous cell carcinoma lymph node metastasis through microbiota-geneset correlation network. *Translational lung cancer research*. Nov 30 2023;12(11):2245-2259. doi:10.21037/tlcr-23-357

48. Liu Y, Yuan X, Chen K, et al. Clinical significance and prognostic value of Porphyromonas gingivalis infection in lung cancer. *Translational oncology*. Jan 2021;14(1):100972. doi:10.1016/j.tranon.2020.100972

49. Tzeng A, Sangwan N, Jia M, et al. Human breast microbiome correlates with prognostic features and immunological signatures in breast cancer. *Genome medicine*. Apr 16 2021;13(1):60. doi:10.1186/s13073-021-00874-2

50. Narunsky-Haziza L, Sepich-Poore GD, Livyatan I, et al. Pan-cancer analyses reveal cancer-type-specific fungal ecologies and bacteriome interactions. *Cell*. Sep 29 2022;185(20):3789-3806.e17. doi:10.1016/j.cell.2022.09.005

51. Fu A, Yao B, Dong T, et al. Tumor-resident intracellular microbiota promotes metastatic colonization in breast cancer. *Cell*. Apr 14 2022;185(8):1356-1372.e26. doi:10.1016/j.cell.2022.02.027

52. Thyagarajan S, Zhang Y, Thapa S, et al. Comparative analysis of racial differences in breast tumor microbiome. *Sci Rep*. Aug 24 2020;10(1):14116. doi:10.1038/s41598-020-71102-x

53. Parida S, Wu S, Siddharth S, et al. A Procarcinogenic Colon Microbe Promotes Breast Tumorigenesis and Metastatic Progression and Concomitantly Activates Notch and β-Catenin Axes. *Cancer discovery*. May 2021;11(5):1138-1157. doi:10.1158/2159-8290.Cd-20-0537

54. Wang H, Rong X, Zhao G, et al. The microbial metabolite trimethylamine N-oxide promotes antitumor immunity in triple-negative breast cancer. *Cell Metab*. Apr 5 2022;34(4):581-594.e8. doi:10.1016/j.cmet.2022.02.010

55. Klann E, Williamson JM, Tagliamonte MS, et al. Microbiota composition in bilateral healthy breast tissue and breast tumors. *Cancer causes & control : CCC*. Nov 2020;31(11):1027-1038. doi:10.1007/s10552-020-01338-5

56. Parhi L, Alon-Maimon T, Sol A, et al. Breast cancer colonization by Fusobacterium nucleatum accelerates tumor growth and metastatic progression. *Nat Commun*. Jun 26 2020;11(1):3259. doi:10.1038/s41467-020-16967-2

57. Ma W, Zhang L, Chen W, et al. Microbiota enterotoxigenic Bacteroides fragilis-secreted BFT-1 promotes breast cancer cell stemness and chemoresistance through its functional receptor NOD1. *Protein & cell*. May 28 2024;15(6):419-440. doi:10.1093/procel/pwae005

58. Li J, Zhang Y, Cai Y, et al. Multi-omics analysis elucidates the relationship between intratumor microbiome and host immune heterogeneity in breast cancer. *Microbiology spectrum*. Apr 2 2024;12(4):e0410423. doi:10.1128/spectrum.04104-23

59. Wang Q, Zhao L, Han L, et al. The differential distribution of bacteria between cancerous and noncancerous ovarian tissues in situ. *Journal of ovarian research*. Jan 18 2020;13(1):8. doi:10.1186/s13048-019-0603-4

60. Sheng D, Yue K, Li H, et al. The Interaction between Intratumoral Microbiome and Immunity Is Related to the Prognosis of Ovarian Cancer. *Microbiology spectrum*. Mar 28 2023;11(2):e0354922. doi:10.1128/spectrum.03549-22

61. Brewster WR, Burkett WC, Ko EM, Bae-Jump V, Nicole McCoy A, Keku TO. An evaluation of the microbiota of the upper reproductive tract of women with and without epithelial ovarian cancer. *Gynecologic oncology reports*. Aug 2022;42:101017. doi:10.1016/j.gore.2022.101017

62. Kim SI, Kang N, Leem S, et al. Metagenomic Analysis of Serum Microbe-Derived Extracellular Vesicles and Diagnostic Models to Differentiate Ovarian Cancer and Benign Ovarian Tumor. *Cancers*. May 21 2020;12(5)doi:10.3390/cancers12051309

63. Huang ST, Chen J, Lian LY, et al. Intratumoral levels and prognostic significance of Fusobacterium nucleatum in cervical carcinoma. *Aging*. Nov 14 2020;12(22):23337-23350. doi:10.18632/aging.104188

64. Liu J, Luo M, Zhang Y, Cao G, Wang S. Association of high-risk human papillomavirus infection duration and cervical lesions with vaginal microbiota composition. *Annals of translational medicine*. Sep 2020;8(18):1161. doi:10.21037/atm-20-5832

65. Chen Y, Li C, Wang X, Zhang CL, Ren ZG, Wang ZQ. Oral microbiota distinguishes patients with osteosarcoma from healthy controls. *Front Cell Infect Microbiol*. 2024;14:1383878. doi:10.3389/fcimb.2024.1383878
